# Distinct gene regulatory programs define the inhibitory effects of liver X receptors and PPARG on cancer cell proliferation

**DOI:** 10.1186/s13073-016-0328-6

**Published:** 2016-07-11

**Authors:** Daniel Savic, Ryne C. Ramaker, Brian S. Roberts, Emma C. Dean, Todd C. Burwell, Sarah K. Meadows, Sara J. Cooper, Michael J. Garabedian, Jason Gertz, Richard M. Myers

**Affiliations:** HudsonAlpha Institute for Biotechnology, Huntsville, AL 35806 USA; Department of Genetics, University of Alabama at Birmingham, Birmingham, AL 35294 USA; Departments of Microbiology and Urology, New York University, New York, NY 10016 USA; Department of Oncological Sciences, Huntsman Cancer Institute, University of Utah, Salt Lake City, UT 84112 USA

**Keywords:** Nuclear receptors, LXR, PPARG, Cell proliferation, Metabolism, Energy homeostasis, Transcription, Chromatin state dynamics, RNA-seq, ChIP-seq

## Abstract

**Background:**

The liver X receptors (LXRs, NR1H2 and NR1H3) and peroxisome proliferator-activated receptor gamma (PPARG, NR1C3) nuclear receptor transcription factors (TFs) are master regulators of energy homeostasis. Intriguingly, recent studies suggest that these metabolic regulators also impact tumor cell proliferation. However, a comprehensive temporal molecular characterization of the LXR and PPARG gene regulatory responses in tumor cells is still lacking.

**Methods:**

To better define the underlying molecular processes governing the genetic control of cellular growth in response to extracellular metabolic signals, we performed a comprehensive, genome-wide characterization of the temporal regulatory cascades mediated by LXR and PPARG signaling in HT29 colorectal cancer cells. For this analysis, we applied a multi-tiered approach that incorporated cellular phenotypic assays, gene expression profiles, chromatin state dynamics, and nuclear receptor binding patterns.

**Results:**

Our results illustrate that the activation of both nuclear receptors inhibited cell proliferation and further decreased glutathione levels, consistent with increased cellular oxidative stress. Despite a common metabolic reprogramming, the gene regulatory network programs initiated by these nuclear receptors were widely distinct. PPARG generated a rapid and short-term response while maintaining a gene activator role. By contrast, LXR signaling was prolonged, with initial, predominantly activating functions that transitioned to repressive gene regulatory activities at late time points.

**Conclusions:**

Through the use of a multi-tiered strategy that integrated various genomic datasets, our data illustrate that distinct gene regulatory programs elicit common phenotypic effects, highlighting the complexity of the genome. These results further provide a detailed molecular map of metabolic reprogramming in cancer cells through LXR and PPARG activation. As ligand-inducible TFs, these nuclear receptors can potentially serve as attractive therapeutic targets for the treatment of various cancers.

**Electronic supplementary material:**

The online version of this article (doi:10.1186/s13073-016-0328-6) contains supplementary material, which is available to authorized users.

## Background

Metazoan systems are exposed to a multitude of extracellular stimuli, including both compounds that are endogenously synthesized and those obtained through diet [1–3]. In response to these environmental signals, cells activate intricate transcriptional programs [1–3]. The careful interplay between extracellular cues and intracellular gene expression responses maintains normal cellular equilibrium and is key for cellular differentiation and development [4–7]. Despite this importance, more work is needed for a thorough, molecular characterization of the temporal gene regulatory responses to diverse external signals.

The liver X receptors (LXRs), also referred to as Nuclear Receptor Subfamily 1, Group H, Member 2 (NR1H2) and Nuclear Receptor Subfamily 1, Group H, Member 3 (NR1H3), and peroxisome proliferator-activated receptor gamma (PPARG), also referred to as Nuclear Receptor Subfamily 1 Group C Member 3 (NR1C3), are ligand-activated nuclear receptor transcription factors (TFs) that respond to oxysterols [8] and fatty acids [9], respectively. LXRs are comprised of two isoforms (alpha and beta; LXRA and LXRB) that have related functions and tissue expression profiles, including a common affinity for oxysterols [8], while PPARG is activated by distinct fatty acids [9]. LXRs and PPARG maintain energy homeostasis by regulating lipid [10, 11] and glucose [12, 13] metabolism. Intriguingly, they are also key modulators of inflammatory responses [14]. These metabolic TFs are also involved in several diseases, including diabetes [12, 15–19], obesity [20–24], atherosclerosis [25–29], and cancer [30–33].

Protective functions have consistently been reported for LXRs in a variety of diverse cancers. For instance, diets rich in plant-derived phytosterols, a putative LXR agonist [34], were shown to decrease risk of breast [35], gastric [36], and lung [37] cancers. Importantly, a protective effect for phytosterols in colorectal cancer has also been demonstrated using cell lines and animal models [38]. Administration of an LXR agonist also suppressed the growth of LNCaP prostate cancer cell tumor xenografts in mice [39] while genetic ablation of LXRB results in gallbladder carcinogenesis [40]. Moreover, specific LXR activation leads to anti-proliferative effects in breast [41], prostate [33], and colorectal [32] cancer cells. Several mechanisms have been proposed for these LXR-mediated effects. For instance, cell cycle inhibition [32, 33], induction of apoptosis [42], and ligand deprivation [43] have all been described. The role of PPARG in tumorigenesis is complex and its effects appear to be tissue-specific. Several studies have suggested protective effects in breast [44], hepatic [45], and lung [31] cancers. A recent study characterized a PPARG anti-proliferative effect in lung cancer that was proposed to be due to the regulation of cellular reactive oxygen species via activation of pyruvate dehydrogenase kinase 4 (PDK4) and beta-oxidation of fatty acids [31]. However, in colorectal cancer, both tumor-suppressive [46] and tumor-facilitating [30, 47] functions have been proposed for PPARG.

To better understand the underlying gene regulatory functions of LXRs and PPARG that lead to metabolic reprogramming in cancer cells, we performed a comprehensive genome-wide analysis of LXR and PPARG activity in HT29 colorectal cancer cells. Despite common physiological effects on energy homeostasis, paradoxically, the genome-wide molecular programs elicited by LXRs and PPARG appear to be temporally distinct. Our results highlight the advantages of integrating multiple levels of transcriptional regulation rather than relying on a single feature. From a broader perspective, this work supports the notion that common phenotypic effects can be mediated through distinct gene regulatory mechanisms.

## Methods

### Cell culture

HT29 colorectal cancer cells were obtained from ATCC and grown under recommended cell culture conditions using McCoy’s 5A media containing 10 % fetal bovine serum (FBS) and 1 % penicillin/streptomycin. GW3965 (Sigma), T0901317 (Santa Cruz), and rosiglitazone (Sigma) powder were diluted in DMSO and added to media at a final concentration of 10 μM. Prior to drug treatments, HT29 cells were grown in phenol-red free McCoy’s 5A media containing 10 % charcoal/dextran-treated FBS and 1 % penicillin/streptomycin.

### Cell phenotype assays

Cell-based phenotypic assays were performed in 96-well cell culture plates. Cell proliferation measurements were determined using the CyQuant assay (Invitrogen) for DNA content and CellTiter-Glo Luminescent Cell Viability Assay (Promega) for ATP levels. Glutathione levels for oxidative stress were determined using the GSH-Glo Glutathione Assay (Promega).

### Metabolomic experiments

For analyses of metabolites, cell cultures were washed with phosphate-buffered saline, molecular biology-grade water and then subsequently pelleted for mass spectrometry. Cell pellets were resuspended in 50 % methanol, shaken at 4 °C for 30 minutes and centrifuged at 12,000 rpm for 10 minutes. Duplicate aliquots of supernatant were dried at 55 °C for 60 minutes using a vacuum concentrator system (Labconco). Derivatization by methoximation and trimethylsilyation was performed as previously described [48]. All derivatized samples were analyzed on a Leco Pegasus 4D system (GCxGC-TOFMS), controlled by the ChromaTof software (Leco, St. Joseph, MI, USA). Peaks were identified by spectral match using the NIST, GOLM, and Fiehn libraries (Leco) and confirmed by running derivatized standards (Sigma). Peaks present in less than two-thirds of samples were excluded from further analysis. Sample replicates were averaged and peak areas were sum normalized prior to comparisons.

### Transfection of LXRA transgene plasmid constructs

Flag-tagged (3-prime 3X-Flag tags separated by glycine spacers) LXRA cDNA plasmid constructs were transfected (Fugene) into HT29 cells. HT29 cells were subsequently selected using G418 (Invitrogen) and expanded prior to GW3965 drug treatment and ChIP-seq experimentation.

### ChIP-seq assays and analysis

ChIP-seq was performed as previously outlined [49]. Antibodies for H3K27ac (Abcam, ab4729), H3K36me3 (Abcam, ab9050), RNA polymerase II (RNAP2; Abcam, ab5408), LXRB (Active Motif, 61177), PPARG (Santa Cruz, sc-7273), and Flag (Sigma, F1804) were used.

### RNA-seq experimentation and analysis

RNA-seq was performed as previously outlined [50] using Nextera transposases (Illumina). Briefly, cells were lysed with Buffer RLT (Qiagen) containing 10 % beta-mercaptoethanol. The Norgen Animal Tissue RNA Purification Kit (Norgen Biotek) was used to isolate RNA from cells. The Dynabead mRNA Purification Kit (Invitrogen) beads were used to isolate mRNA transcripts and cDNA synthesis was performed using Superscript reverse transcriptase (Invitrogen). Nextera transposases (Illumina) were used to fragment cDNA prior to next-generation sequencing. All experimental treatments at all time points were performed in quadruplicate.

### Data analysis

Two-sided Student’s *t*-tests were performed to assign significance for cellular phenotypic assays and metabolite levels. Only reproducible binding sites identified by replicate ChIP-seq experiments were reported and used for downstream analyses. ChIP-seq peaks were identified using MACS peak caller [51], while motif analysis was performed using MEME [52] on the top 500 most enriched binding sites. For normalized ChIP-seq read-depth analyses, the number of reads were tabulated across binding site coordinates and normalized to the total number of aligned reads obtained for each ChIP-seq experiment. For mapping reads, we utilized a 100-bp sequence centered on the peak summit of each binding site. For ranking temporal changes in read depth, we generated a linear model comparing replicate ChIP-seq read enrichment values between two time points. Sites with altered enrichment were subsequently divided by slope and *p* value ranked. The top 50 % of *p* value-ranked sites from each category were used for cofactor motif analyses. For assigning TF motif fold enrichments, we compared the total number of motifs found across a set of binding sites with the number of motifs identified after scrambling binding site sequences. For RNAP2 read depth promoter analyses, sequences within 500 bp of transcription start sites were used as promoters. Connectivity maps were generated using Cytoscape (http://www.cytoscape.org/) and Enrichment map [53]. Kyoto Encyclopedia of Genes and Genomes (KEGG) gene pathway enrichments were generated using Gene Set Enrichment Analysis (GSEA; http://software.broadinstitute.org/gsea/msigdb/annotate.jsp). Wilcoxon rank sum tests were used to assign significance for H3K36me3 enrichment analyses. Differential gene expression was determined using DESeq [54]. High-confidence GW3965 + T0901317 responsive genes were assigned for all targets that passed significance and fold change cutoffs across both drug treatments. For comparison of gene expression with RNAP2 and H3K36me3 occupancy, if a gene harbored multiple promoters and/or transcripts, the promoter and/or gene body coordinate exhibiting the strongest read enrichment was used.

## Results

### LXRs and PPARG generate common phenotypic effects

**Fig. 1:**
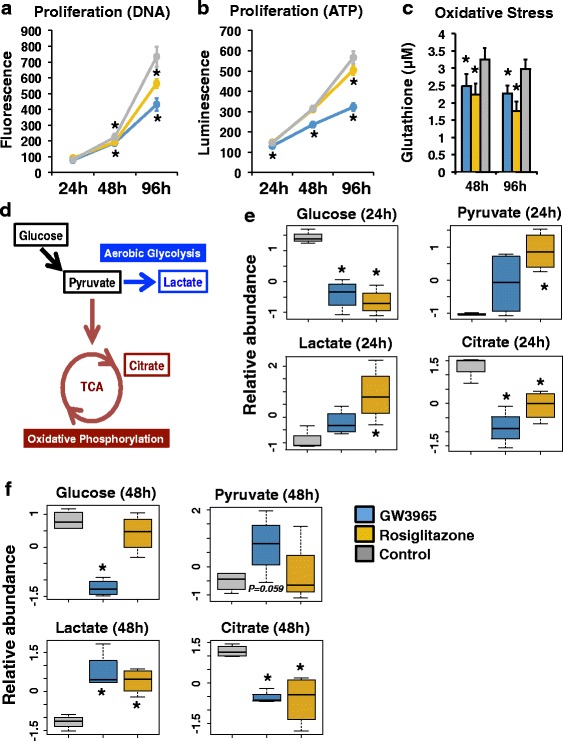
Effects of LXRs and PPARG on cellular phenotypes and metabolites. **a** Proliferation assays using DNA content after 24, 48, and 96 h of drug treatment (24-h n = 10 for each treatment; 48/96-h n = 12 for each treatment). Luminescence values are presented for GW3965 (*blue*, LXR agonist), rosiglitazone (*gold*, PPARG agonist), and DMSO (*gray*, vehicle control) treatment. *p < 0.01. **b** Proliferation assays using ATP levels after 24, 48, and 96 h of drug treatment (24-h n = 15 DMSO, n = 8 rosiglitazone, n = 7 GW3965; 48/96-h n = 18 DMSO, n = 9 rosiglitazone, n = 9 GW3965). Luminescence values are presented for GW3965 (*blue*, LXR agonist), rosiglitazone (*gold*, PPARG agonist), and DMSO (*gray*, vehicle control) treatment. *p < 0.01. **c** Oxidative stress assays after 48 and 96 h of drug treatment (n = 18 DMSO, n = 9 rosiglitazone, n = 9 GW3965). Glutathione concentrations are presented for GW3965 (*blue*, LXR agonist), rosiglitazone (*gold*, PPARG agonist), and DMSO (*gray*, vehicle control) treatment. *p < 0.01. **d** The carbohydrate metabolism pathway illustrating key metabolites. **e** Relative metabolite abundance after 24 h of GW3965 (*blue*, LXR agonist), rosiglitazone (*gold*, PPARG agonist), and DMSO (*gray*, vehicle control) treatment. *p < 0.05. **f** Relative metabolite abundance after 48 h of GW3965 (*blue*, LXR agonist), rosiglitazone (*gold*, PPARG agonist), and DMSO (*gray*, vehicle control) treatment. *p < 0.05. Error bars represent standard deviation

We evaluated the proliferative and metabolic effects of LXRs and PPARG in HT29 colorectal cancer cells with several cellular assays. First, we assayed cellular proliferation by measuring DNA and ATP content in cells after 24, 48, and 96 h of treatment with the LXR agonist GW3965 and the PPARG agonist rosiglitazone (Fig. 1a, b) and by comparing the resulting cellular responses to control culture conditions containing the carrier DMSO. The evaluation of both DNA and ATP confirmed an inhibitory effect on colorectal cancer cell proliferation, supporting conclusions of previous reports [32, 46]. Notably, these anti-proliferative effects became more pronounced over time. In light of the functions of LXRs and PPARG as regulators of energy homeostasis, we also measured glutathione levels in drug-treated cells (Fig. 1c). LXR and PPARG activation significantly decreased glutathione levels, highlighting a potential inhibitory effect on proliferation through increased oxidative stress, a finding that has been reported for PPARG in lung cancer cells [31].

To better characterize cellular metabolic alterations, we next assessed metabolite levels through mass spectrometry of HT29 cell lysate after drug treatment (Fig. 1d–f). We focused our analyses on carbohydrate metabolism and evaluated key metabolites involved in oxidative phosphorylation and aerobic glycolysis (Fig. 1d). We found that LXR and PPARG activation led to significant decreases in glucose and citrate levels, as well as to significant increases in pyruvate and lactate levels after drug treatment (Fig. 1e, f), supporting an increase in cellular aerobic glycolysis at the expense of oxidative phosphorylation. Although both drug treatments generated the same cellular response, the temporal dynamics of these metabolic effects were distinct; rosiglitazone produced more substantial effects at 24 h, while several of the GW3965-mediated changes were evident only after 48 h of drug treatment. Apart from glucose pathway metabolites, we also identified a variety of compounds that were significantly altered in response to drug treatment (Additional file 1: Figure S1). In all these cases, LXR and PPARG activation generated identical alterations, highlighting a common mechanism of metabolic reprogramming.

### Distinct transcriptional profiles from LXR and PPARG signaling

**Fig. 2:**
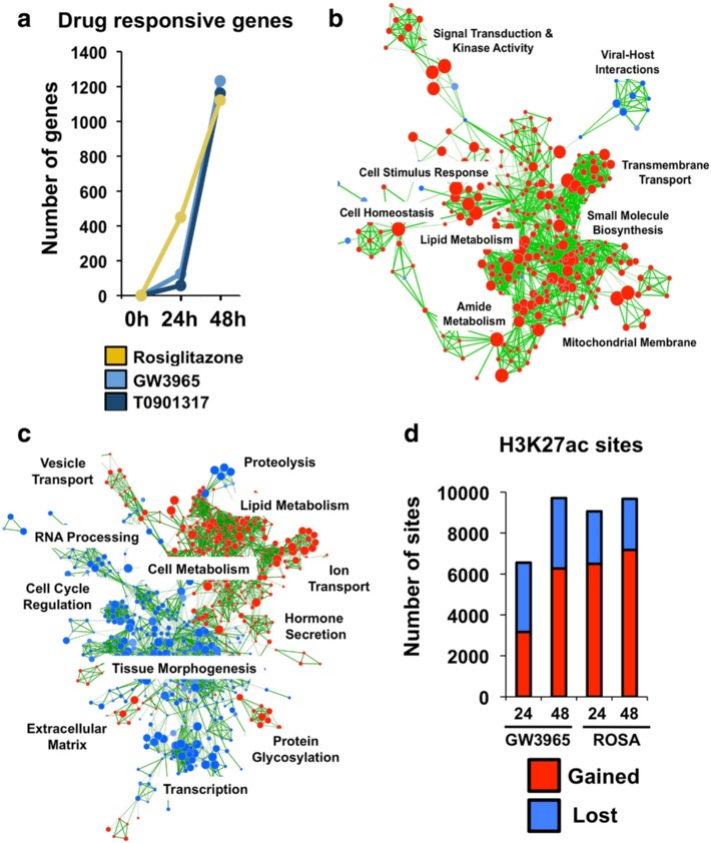
Chromatin dynamics and gene expression profiles from nuclear receptor activation. **a** The number of differentially regulated genes (up- and down-regulated) after 24 and 48 h of drug treatment (adjusted *p* < 0.01 and fold change cutoff ±2). **b** Gene Ontology connectivity map for GW3965 at 24 h for up-regulated (*red*) and down-regulated (*red*) pathways. **c** Gene Ontology connectivity map for GW3965 at 48 h for up-regulated (*red*) and down-regulated (*red*) pathways. **d** The number of H3K27ac sites gained (*red*) and lost (*blue*) after 24 and 48 h of GW3965 and rosiglitazone drug treatment

We next evaluated changes to the cellular transcriptome after GW3965 and rosiglitazone drug treatments. We performed RNA-seq under control culture conditions (DMSO), as well as after 24 and 48 h of drug treatment. For each time point, we calculated the number of differentially regulated genes (adjusted *p* < 0.01, fold change cutoff ±2) in response to each drug (Table 1 and Fig. 2a). The PPARG response generated a near linear curve in the number of responsive genes. By contrast, the GW3965 treatment was delayed at 24 h and the vast majority (80.6 %) of these early genes were up-regulated. To validate this slow response, we performed RNA-seq by using a different LXR agonist, T0901317 (Fig. 2a); the resulting transcriptional response also produced a stalled expression profile. Notably, both GW3965 and T0901317 gene sets were highly correlated (Additional file 1: Figure S2a). By further integrating these gene sets, we generated a list of high-confidence, LXR target genes (GW3965 + T0901317). Supporting our observations, GW3965 + T0901317 gene targets also produced a stalled response (Additional file 1: Figure S2b). The use of a lower gene expression fold change cutoff (±1.5) generated an identical transcriptional pattern for all drug treatments (Additional file 1: Figure S3). Importantly, these gene expression changes are also in agreement with changes in cellular metabolites we described above.

**Table 1:** Total number of differentially regulated genes

	24-h treatment				48-h treatment			
	Fold change ≥1.5	Fold change ≤ −1.5	Fold change ≥2	Fold change ≤ −2	Fold change ≥1.5	Fold change ≤ −1.5	Fold change ≥2	Fold change ≤ −2
Rosiglitazone	421	646	273	177	1019	1396	416	701
GW3965	223	83	100	24	1846	1646	698	535
T0901317	161	10	57	1	1538	1578	589	560
GW3965 + T0901317	119	5	47	0	1040	1141	357	395

The total number of differentially regulated genes (up- and down-regulated) using two distinct fold change cutoffs (*p* < 0.01) from all drug treatments at 24- and 48-h time points is shown. For GW3965 + T0901317 high confidence LXR target gene analyses, all genes exceeded fold change and *p* value cutoffs across both drug treatments

We next compared the gene expression data between rosiglitazone and both GW3965 and T0901317 drug responses. Despite common cellular phenotypes from LXR and PPARG signaling, only a subset of genes was responsive to both agonists (Table 1). Interestingly, many of these common targets were regulated in an opposing manner, underscoring distinct gene regulatory functions of both nuclear receptors at the molecular level. Although decreasing the fold change cutoff (±1.5) led to stronger overlap, the number of genes regulated in an opposing manner similarly increased (Table 2).

**Table 2:** Analysis of overlapping LXR- and PPARG-responsive genes

	Overlap 24 h (%)	Concordant 24 h	Discordant 24 h	Percentage concordant 24 h	Overlap 48 h (%)	Concordant 24 h	Discordant 24 h	Percentage concordant 48 h
GW3965 (fc ±2)	32 (25.8 %)	29	3	90.6 %	203 (18.2 %)	119	84	58.6 %
T0901317 (fc ±2)	9 (15.5 %)	8	1	88.9 %	189 (16.9 %)	70	119	37.0 %
GW3965 (fc ±1.5)	119 (38.9 %)	100	19	84.0 %	790 (35.1 %)	381	409	48.2 %
T0901317 (fc ±1.5)	60 (35.1 %)	41	19	68.3 %	775 (32.1 %)	222	553	28.6 %

The overlap between LXR-responsive genes (from GW3965 and T0901317 treatments) with PPARG-responsive genes (from rosiglitazone treatment) is shown at both 24 and 48 h. The number and percentage of total responsive genes is shown. The number of genes and percentages that show concordant and opposing changes in differential expression across all drug comparisons is also given. Hypergeometric tests of gene overlaps for both drug comparisons and time points were highly significant (*p* < 1e-16)

We performed genetic pathway enrichment analyses based on this list of differentially expressed genes. Gene Ontology enrichment mapping [53] identified wide alterations in pathways affected by GW3965 treatment between 24 and 48 h (Fig. 2b, c). For instance, the 24-h network consisted predominantly of up-regulated pathways involved in lipid metabolism, signal transduction, small molecule biosynthesis, and transmembrane transport. By contrast, at 48 h, we identified a large set of down-regulated pathways involved in cell cycle regulation, transcription, and tissue morphogenesis. This LXR network topology was distinct from the PPARG response (Additional file 1: Figure S4), where 24- and 48-h rosiglitazone inductions produced concordant pathway enrichments predominantly involved in cell metabolism (up-regulated) and membrane transport (down-regulated).

We also performed enrichment analyses using the KEGG pathway dataset from GSEA [55]. At 24 h, the GW3965 and T0901317 drug treatment produced significantly up-regulated pathways involved in lipid and energy metabolism (PPAR signaling, fatty acid metabolism, pyruvate metabolism, etc.), including ABC transporters. Although these patterns were maintained at 48 h, several key pathways and processes, including MAPK, TGF-beta, cell cycle, and Wnt, were significantly down-regulated by both drugs. For rosiglitazone treatment, the up-regulated and down-regulated KEGG pathways were more consistent between 24 and 48 h. A detailed list of all KEGG pathway enrichments can be found in Additional file 2: Table S1. Pathway enrichments for genes commonly induced by both LXR and PPARG activation are also tabulated in Additional file 3: Table S2.

### Chromatin state dynamics support cellular transcriptional effects

We further determined whether the more gradual GW3965 gene expression coincides with slower chromatin state dynamics. We performed replicate ChIP-seq experiments for the histone-3 lysine-27 acetylation (H3K27ac) chromatin modification under control (i.e., DMSO carrier only) culture conditions and after 24- and 48-h drug treatments. Because H3K27ac is a modification that appears at active regulatory elements [56], this temporal characterization provides a genome-wide picture illustrating changes to the *cis*-regulatory architecture upon LXR and PPARG activation. Using data from control culture conditions as a reference, we identified H3K27ac sites that were gained or lost after drug treatment at both time points (Fig. 2d; Additional file 1: Figure S5). We next compared the chromatin responses between drug treatments and identified a substantially larger number of H3K27ac sites that were gained at 24 h in response to rosiglitazone (6500 versus 3162 loci; 51 % more), mirroring transcriptional effects. By 48 h, the difference between both drugs substantially diminished (7179 versus 6273 loci; 13 % more). Interestingly, the degree of H3K27ac site loss was similar between drug treatments (Fig. 2d; Additional file 1: Figure S5). We further identified a substantial amount of overlap in H3K27ac binding events that were gained between drug treatments and their associated genetic pathway enrichments (Additional file 1: Figure S6).

We next compiled all time points for each drug treatment separately to identify loci showing dynamic changes in H3K27ac enrichment across the entire duration of treatment (Additional file 1: Figure S7). We identified all GW3965- and rosiglitazone-induced regulatory sites showing differences in normalized read depth from 0 to 24 h and from 24 to 48 h of drug treatment. The vast majority of dynamic H3K27ac sites exhibited a gradual increase or decrease in H3K27ac occupancy over time, suggesting these analyses are identifying nuclear receptor-mediated regulatory events. Further validating our approach and conclusions above, regulatory sites gaining H3K27ac occupancy throughout the time course were enriched near the promoters of genes up-regulated at 48 h after GW3965 and rosiglitazone drug treatments (Additional file 1: Figure S8). This enrichment was also observed using GW3965 + T0901317 target genes (Additional file 1: Figure S9). We further performed Genomic Regions Enrichment of Annotations Tool (GREAT) pathway analyses [57], which confirmed that sites with temporally increasing H3K27ac occupancy were enriched near relevant genes involved in cell metabolism and/or implicated in colorectal cancer (Additional file 1: Figures S10 and S11). Taken together, these additional data further support the notion that LXR and PPARG direct temporally distinct gene regulatory responses through alterations in chromatin state.

### LXRB and PPARG have temporally opposing binding patterns

**Fig. 3:**
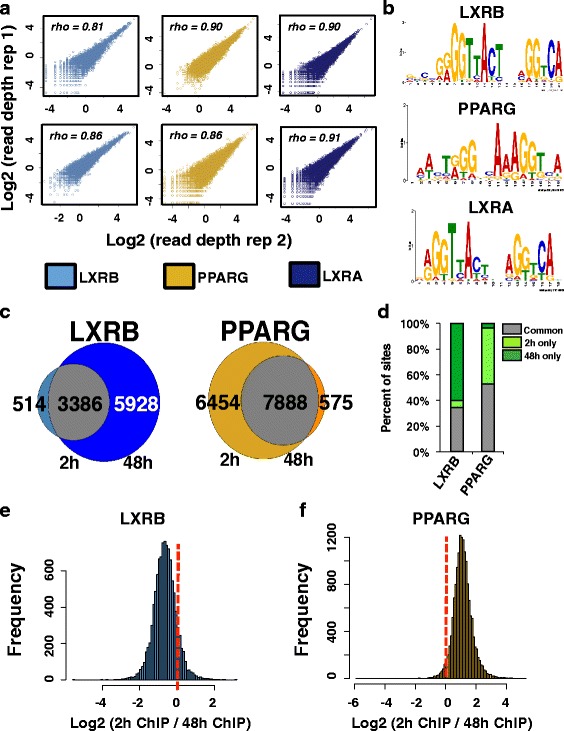
Genome-wide binding profiles of LXRs and PPARG. **a** Normalized sequencing read depth rank correlation (*top left*) at all ChIP-seq binding sites identified across replicate LXRB (*light blue*), PPARG (*gold*), and LXRA (*dark blue*) ChIP-seq experiments after 2 and 48 h of drug treatment. The *top panel* shows 2-h drug treatment replicate ChIP-seq comparisons while the *bottom panel* displays 48-h comparisons. **b** Enriched canonical LXR and PPARG motif identified in corresponding ChIP-seq data. **c** Venn diagram comparisons of binding events between 2 and 48 h of drug treatment. Overlapping sites are shown in *gray*, while sites identified at only 2 or 48 h are shown at the *left* and *right*, respectively. The number of sites in each category is presented for each category. **d** The percentage of all LXRB and PPARG sites that were identified at *2 h only* (*light green*), *48 h only* (*dark green*), and common to both time points (*common*, *gray*). **e** Read depth ratios (*x-axis*) at all LXRB binding sites. Negative values highlight stronger enrichment after 48 h of GW3965 treatment while positive values denote stronger occupancy after 2 h. **f** Read depth ratios (*x-axis*) at all PPARG binding sites. Negative values highlight stronger enrichment after 48 h of rosiglitazone treatment while positive values denote stronger read enrichment after 2 h

To interrogate LXR and PPARG TF occupancy during our drug treatment regime, we performed replicate ChIP-seq experiments for LXRA (NR1H3), LXRB (NR1H2), and PPARG (NR1C3). We used an early (2-h) and a late (48-h) time point for these experiments to further preserve temporal information. These two datasets spanned the entire temporal molecular characterization and allowed for an examination of the immediate effects of drugs on their direct protein targets. We obtained high-quality antibodies for LXRB and PPARG; however, because we could not identify a suitable commercial ChIP-seq grade antibody for LXRA, we engineered HT29 cells to express a C-terminal Flag-tagged LXRA transgene protein and used a Flag antibody for ChIP-seq experiments. Importantly, normalized sequencing read depth at identified LXRA/B or PPARG binding sites was highly concordant across replicate ChIP-seq experiments for both time points (Fig. 3a). Additionally, for all datasets, the canonical LXR and PPARG motifs were significantly enriched, supporting the notion that our cistromes are identifying true nuclear receptor binding events (Fig. 3b). Collectively, we identified 18,653, 3,900 and 14,360 binding sites at 2 h and 17,576, 9,335 and 8463 binding sites at 48 h for LXRA, LXRB, and PPARG, respectively. Moreover, ~80 % of the endogenous LXRB binding sites were identified by the Flag-tagged LXRA protein at both time points, suggesting substantial redundancy between both LXR proteins.

We next integrated the 2-h and 48-h binding information for LXRB and PPARG proteins, as these datasets illustrate temporal occupancies of TFs under endogenous promoter control. Intriguingly, LXRB and PPARG generated opposing profiles (Fig. 3c, d); the number of LXRB binding events increased significantly with time (514 and 5928 binding sites specific to 2 and 48 h, respectively), while PPARG binding exhibited strongest occupancy at 2 h (6454 and 575 binding sites specific to 2 and 48 h, respectively). As these results relied on qualitative metrics, we next applied quantitative approaches to validate these observations. We pooled all LXRB and PPARG binding events for both time points and calculated the normalized sequencing read depth for all ChIP-seq experiments at this complete set of sites. In agreement with our data above, the resulting histograms for all LXRB and PPARG binding sites identified global shifts in distribution of binding enrichment (Fig. 3e, f); LXRB exhibited stronger occupancy at 48 h for the majority of all binding events, while PPARG binding showed the opposing pattern. Taken together, these data highlight temporally distinct, genome-wide occupancy for LXRB and PPARG proteins and further support the more immediate cellular response from rosiglitazone compared with GW3965 treatment.

### AP1 TFs are key cofactors of LXRs and PPARG

**Fig. 4:**
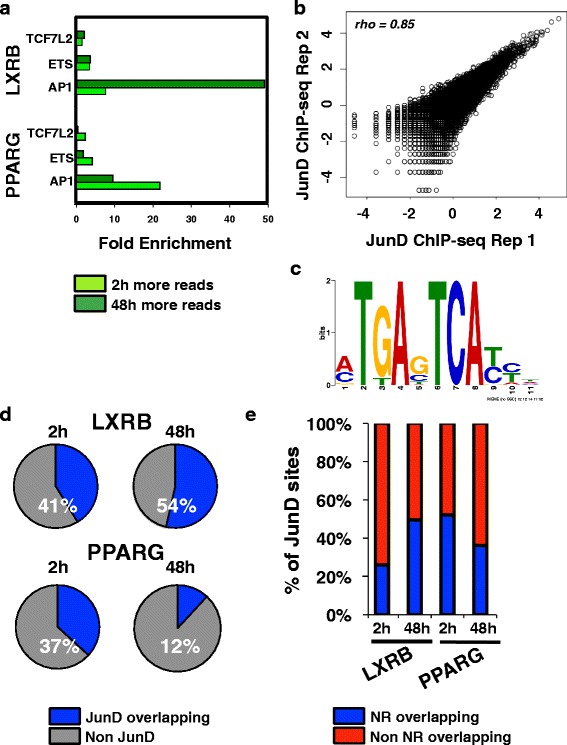
Cofactor enrichment and genome-wide binding patterns. **a** The fold enrichment of various canonical motifs for the top 50 % of significance-ranked LXRB and PPARG binding sites that exhibited stronger occupancy at 2 (*light green*) or 48 (*dark green*) h. **b** Normalized sequencing read depth rank correlation (*top left*) at all binding sites identified across replicate JunD ChIP-seq experiments. **c** Enriched AP1 motif identified at JunD ChIP-seq binding sites. **d** Percentage of LXRB and PPARG sites after 2 and 48 h of stimulation coincident with JunD. **e** Percentage of JunD sites overlapping with LXRB and PPARG nuclear receptor (*NR*) binding events after 2 and 48 h of drug treatment

We analyzed the sequence content of LXRB and PPARG binding sites to identify cooperating TFs that may be necessary for LXR and PPARG function. To elucidate potential temporal changes in cofactor preference, we used the top 50 % of sites ranked by significance that displayed stronger occupancy at 2 or 48 h for this assessment. The analysis of several canonical TF sequence motifs at these sites identified a strong enrichment for the AP1 motif (Fig. 4a). Indeed, temporal changes in AP1 motif enrichment differed substantially and in an opposing manner for both nuclear receptors, with more pronounced enrichment at LXRB and PPARG sites harboring strong 48- and 2-h occupancy, respectively.

We next performed ChIP-seq to analyze the binding of JunD, a known heterodimer of the AP1 TF complex [58], to validate these computational observations. We performed replicate JunD ChIP-seq experiments under control culture conditions (DMSO carrier only). As AP1 complexes act as pioneer factors that sit at regulatory elements to open up chromatin [59], their binding is believed to be precede and be independent of subsequent nuclear receptor binding. Replicate experiments were highly concordant (Fig. 4b). As expected, the canonical AP1 motif was highly enriched in these datasets (Fig. 4c). We next compared JunD binding events to LXRB and PPARG cistromes (Fig. 4d, e). Supporting our motif enrichment analysis, 54 % of LXRB sites overlapped with JunD at 48 h, compared with 41 % at 2 h (Fig. 4d). PPARG generated more pronounced changes, with 37 % of PPARG sites co-occurring with JunD at 2 h and with only 12 % of sites at 48 h (Fig. 4d). We also performed the inverse comparison by assessing the percentage of all JunD binding events that co-occurred with LXRB and PPARG sites and this analysis generated the same results (Fig. 4e); a 1.9-fold stronger concordance was identified at 48 h for LXRB, while a 1.4-fold higher overlap was identified at 2 h for PPARG. Using the LXRA ChIP-seq data, we identified similar enrichments patterns for AP1 motif enrichment and JunD co-occupancy as the endogenous LXRB binding profile (Additional file 1: Figure S12).

### LXRs and PPARG maintain temporally distinct gene regulatory activities

**Fig. 5:**
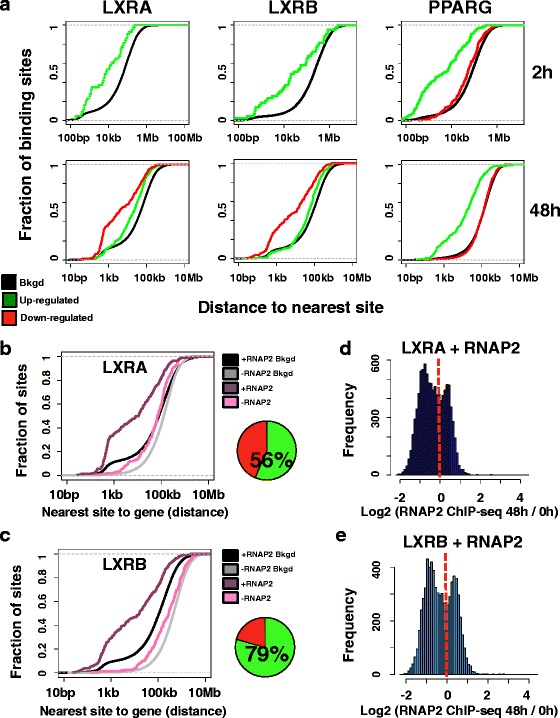
LXRs and PPARG maintain temporally distinct gene regulatory functions. **a** Cumulative distribution functions display the fraction of target gene promoters (*y-axis*) at different distance cutoffs to the nearest LXRA, LXRB, and PPARG binding event (*x-axis*). The nearest binding event to each gene promoter was used. Data for promoters of responsive up-regulated genes (*green*) and repressed (*red*) genes (adjusted *p* < 0.01, fold change cutoff of ±2) are shown. The background distribution using all gene promoters in the genome is also displayed (*Bkgd*, *black*). The *top panel* represents 2-h ChIP-seq data compared with 24-h RNA-seq data while the *bottom panel* compares 48-h ChIP-seq and RNA-seq data. For LXR data, GW3965 + T0901317 responsive genes are shown. Down-regulated curves are absent for 24-h GW3965 + T0901317 datasets as only one target gene was identified. **b** Cumulative distribution functions display the fraction of 48-h GW3965 + T0901317 repressed gene promoters (*y-axis*, adjusted *p* < 0.01, fold change cutoff of ±2) at different distance cutoffs to the nearest LXRA binding event (*x-axis*). LXRA binding events coincident with RNAP2 are in *dark purple* (*+RNAP2*), while LXRA sites devoid of RNAP2 occupancy are denoted in *pink* (*-RNAP2*). The background distribution using all gene promoters in the genome for sites overlapping with RNAP2 (*+RNAP2 Bkgd*, *black*) or for sites devoid of RNAP2 (*-RNAP2 Bkgd*, *gray*) is also graphed. The pie chart depicts the fraction of LXRA sites coincident with RNAP2 (*green*). **c** Cumulative distribution functions display the fraction of 48-h GW3965 + T0901317 repressed gene promoters (*y-axis*, adjusted *p* < 0.01, fold change cutoff of ±2) at different distance cutoffs to the nearest LXRB binding event (*x-axis*). LXRB binding events coincident with RNAP2 are in *dark purple* (*+RNAP2*), while LXRB sites devoid of RNAP2 occupancy are denoted in *pink* (*-RNAP2*). The background distribution using all gene promoters in the genome for sites overlapping with RNAP2 (*+RNAP2 Bkgd*, *black*) or for sites devoid of RNAP2 (*-RNAP2 Bkgd*, *gray*) is also graphed. The pie chart depicts the fraction of LXRB sites coincident with RNAP2 (*green*). **d** Read depth ratios (*x-axis*) of RNAP2 enrichment at LXRA binding sites (*LXRA + RNAP2*). Negative values highlight stronger enrichment under control culture conditions (DMSO; 0 h) while positive values denote stronger enrichment after 48 h of GW3965 treatment. **e** Read depth ratios (*x-axis*) of RNAP2 enrichment at LXRB binding sites (*LXRB + RNAP2*). Negative values highlight stronger enrichment under control culture conditions (DMSO; 0 h) while positive values denote stronger enrichment after 48 h of GW3965 treatment

We integrated our drug treatment gene expression datasets with our nuclear receptor ChIP-seq results to investigate whether there was direct activation and repression through PPARG, LXRA, and LXRB. To identify putative direct target genes of the assayed TFs, we determined the distance of the nearest binding site to promoters of genes and subsequently generated cumulative distribution functions comparing the fraction of genes with binding events at different distance cutoffs (Fig. 5a). For assessing distance enrichments, we used all the genes in the genome to obtain a background distribution. We further split drug-responsive genes (adjusted *p* < 0.01, fold change cutoff ±2) into activated and repressed targets to assign putative regulatory functions. To infer early regulatory functions, we compared 24-h RNA-seq data with 2-h ChIP-seq data and used the 48-h datasets to assess late activities (Fig. 5a). Notably, both LXRA and LXRB generated concordant profiles that were distinct from those of PPARG. During the early drug response, LXRA and LXRB binding events are situated near up-regulated GW3965 + T0901317 target genes. However, during the late response, LXR occupancy occurs near repressed genes. This pattern is in contrast to the PPARG profile, where strong enrichments are observed only for up-regulated target genes. Again, the same LXR enrichment transition was observed when we used a lower fold change cutoff (±1.5), and we further ruled out the possibility that this enrichment was driven by a negative feedback loop on genes significantly activated at 24 h by removing these genes from the analysis (Additional file 1: Figures S13 and S14). We also obtained concordant enrichment transitions for LXRs using only GW3965 gene expression data (Additional file 1: Figures S15–S17).

Diverse genomic and functional studies support the role of RNAP2 as a marker of active, promoter-distal enhancer elements [60, 61]. To determine if these gene promoter enrichments were driven by nearby, functional nuclear receptor binding events, we performed replicate RNA polymerase II (RNAP2) ChIP-seq experiments under control culture conditions (DMSO) and after 24 and 48 h of GW3965 and rosiglitazone treatment. We first compared RNAP2 binding events to the LXR ChIP-seq datasets and divided LXR sites into those that were co-occupied by RNAP2 and sites that were devoid of RNAP2 occupancy. By integrating these distinct loci with GW3965 + T0901317 target genes that were repressed at 48 h, we identified RNAP2 co-occupied LXR sites as the principle drivers of the enrichment with down-regulated gene promoters, pointing to the functionality of these binding events (Additional file 1: Figure S18). We obtained comparable results when we used only GW3965-responsive genes (Fig. 5b, c) and lower fold change cutoffs (Additional file 1: Figure S19). Moreover, the majority of LXRA (56 %) and LXRB (79 %) binding events at 48 h were coincident with RNAP2. Identical analyses using 2-h rosiglitazone PPARG binding results and 24-h rosiglitazone RNAP2 occupancy identified a similar enrichment with RNAP2 co-occupancy at genes up-regulated by rosiglitazone at 24 h (Additional file 1: Figure S20).

To validate the functional likelihood of these binding events further, we compared our nuclear receptor ChIP-seq data with H3K27ac binding data. For all analyses, and using GW3965 + T0901317 repressed genes at 48 h, as well as 24-h rosiglitazone-activated genes, we confirmed that PPARG, LXRA, and LXRB binding sites overlapping with H3K27ac exhibited more pronounced enrichment near these drug responsive genes compared with sites devoid of H3K27ac occupancy (Additional file 1: Figures S21–S23). This observation held true using both GW3965 and high-confidence GW3965 + T0901317 gene sets for LXR comparisons and by controlling for confounding effects from negative feedback loops. Similar enrichments were observed for 24-h up-regulated genes for both LXR nuclear receptors and 48-h up-regulated genes for PPARG (Additional file 1: Figure S24). Notably, LXRA and LXRB binding events coincident with the JunD binding were also preferentially enriched near repressed genes (Additional file 1: Figure S25).

Finally, we performed quantitative analyses at LXRA and LXRB sites that co-occurred with RNAP2 at 48 h to ascertain temporal changes in RNAP2 occupancy, which may further provide mechanistic insight (Fig. 5d, e). Intriguingly, an analysis of normalized RNAP2 read depth at both LXRA and LXRB sites revealed a bimodal distribution, with a subset of sites exhibiting stronger RNAP2 enrichment prior to drug treatment and a fraction of binding events displaying greater occupancy after 48 h of GW3965 treatment. Related assessments of H3K27ac temporal patterns generated more stable distributions (Additional file 1: Figure S26), limiting this bimodal pattern to RNAP2 occupancy. Collectively, these data point to gene-activating roles for PPARG, while LXR proteins seem to harbor more dynamic regulatory activities characterized by early activating effects and late repressing functions.

### LXR repression is consistent with RNAP2 promoter-proximal pausing

**Fig. 6:**
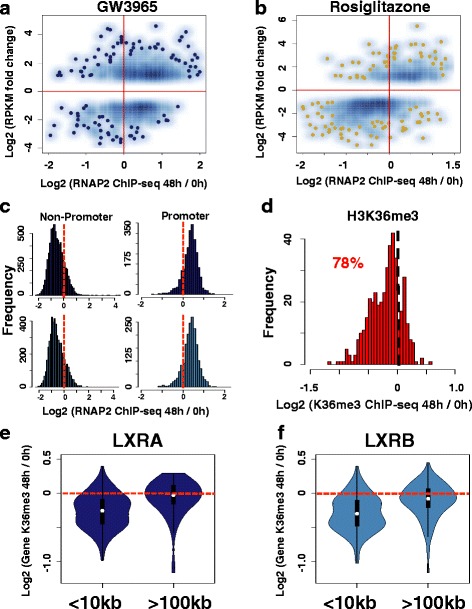
LXR repression is consistent with RNAP2 promoter-proximal pausing. **a** Smooth scatter plot comparing RNA-derived gene expression levels after 48-h GW3965 treatment (*y-axis*, reads per kilobase of transcript per million mapped reads (*RPKM*)) with sequencing read depth ratios of ChIP-derived RNAP2 promoter occupancy (*x-axis*). Read depth at promoters assesses the enrichment after 48 h of GW3965 (*48 h*) normalized to control culture conditions (*0 h*). Data are presented for all GW3965 responsive genes (adjusted *p* < 0.01, fold change cutoff of ±2). **b** Smooth scatter plot comparing RNA-derived gene expression levels after 48 h of rosiglitazone stimulation (*y-axis*, *RPKM*) with sequencing read depth ratios of ChIP-derived RNAP2 promoter occupancy (*x-axis*). Read depth at promoters assesses the enrichment after 48 h of rosiglitazone (*48 h*) normalized to control culture conditions (*0 h*). Data are presented for all rosiglitazone-responsive genes (adjusted *p* < 0.01, fold change cutoff of ±2). **c** Histogram tabulating the read depth ratios (*x-axis*) of RNAP2 enrichment sites co-occupied by LXRA and RNAP2 (*top panel*) and sites co-occupied by LXRB and RNAP2 (*bottom panel*). Co-occupied sites are divided based on promoter location (*Non-Promoter* and *Promoter*). Negative values highlight stronger enrichment under control culture conditions (*0 h*) while positive values denote stronger enrichment after 48 hours of GW3965 treatment. **d** Histogram tabulating the read depth ratios (*x-axis*) of H3K36me3 enrichment in gene bodies for genes down-regulated after 48 h of GW3965 treatment. Negative values highlight stronger enrichment at baseline (*0 h*) while positive values denote stronger enrichment after 48 h of GW3965 treatment. **e** Violin plot of changes in H3K36me3 read depth ratio in gene bodies of repressed genes after 48 h of GW3965 treatment. Plots are displayed for genes that harbor LXRA binding events within 10 kb (<10 kb) from promoters, as well as plots for genes with no evidence of nearby LXRA occupancy (>100 kb). **f** Violin plot of changes in H3K36me3 read depth ratio in gene bodies of repressed genes after 48 h of GW3965 treatment. Plots are displayed for genes that harbor LXRB binding events within 10 kb (<10 kb) of promoters, as well as plots for genes with no evidence of nearby LXRB occupancy (>100 kb)

To infer a potential mechanism for the late, LXR-mediated gene repression described above, we compared RNAP2 binding profiles to RNA-seq results (adjusted *p* < 0.01, fold change cutoff ±2). We first used our RNAP2 ChIP-seq data to determine the predictive value of RNAP2 normalized read depth at promoters as a measurement for global gene expression (Additional file 1: Figure S27). For all drug treatments, we obtained sufficient correlations (rho > 0.72). Having shown the utility of promoter RNAP2 occupancy, we next assessed RNAP2 read depth at promoters of differentially regulated genes (Fig. 6a, b; Additional file 1: Figures S28–S31). During the early response (Additional file 1: Figure S28), both datasets were well correlated; the vast majority of up-regulated genes exhibited stronger RNAP2 promoter enrichment compared with control culture conditions, while down-regulated genes had reduced RNAP2 read depth at their promoters. However, pronounced differences were observed between GW3965 and rosiglitazone treatments at late time points (Fig. 6a, b). Although 81 % of activated GW3965 genes displayed stronger RNAP2 promoter enrichment, paradoxically, only 34 % of repressed genes showed a decrease in RNAP2 promoter occupancy. This effect was not observed for rosiglitazone, where 81 % of up-regulated and 85 % of down-regulated genes maintained correlated changes with RNAP2 promoter occupancy. We further validated this deviation when we used lower fold change cutoffs (Additional file 1: Figures S29 and S30), while swapping binding sites and gene expression datasets between GW3965 and rosiglitazone treatments generated random, overlapping distributions between up- and down-regulated genes (Additional file 1: Figure S31).

We next divided the LXR sites overlapping with RNAP2 sites (LXR + RNAP2) into promoter binding events and LXR + RNAP2 binding events distal to promoters (±500 bp) to determine if promoter association can explain the observed bimodal distribution of RNAP2 read enrichment (Fig. 5d, e). Notably, by separating LXR + RNAP2 co-occupied sites based on overlap with promoter regions (Fig. 6c), we were able to explain the temporal RNAP2 occupancy distribution; overlapping sites at promoters exhibited stronger RNAP2 enrichment at 48 h, while LXR binding events outside of promoters displayed diminished RNAP2 occupancy at 48 h. The University of California Santa Cruz (UCSC) Genome browser images (https://genome.ucsc.edu/) of raw ChIP-seq signal at several loci spanning 48-h, GW3965-repressed genes are provided in Additional file 1: Figures S32–S35.

The observation of a late, stronger RNAP2 promoter occupancy at LXR responsive genes is difficult to reconcile with the decrease in RNA expression during the late drug response. These effects may be mediated by post-transcriptional regulation, for example, by enhancing transcript degradation or, alternatively, by RNAP2 promoter-proximal pausing, with increased enrichment stemming from longer RNAP2 promoter residency times. To potentially distinguish between these two possibilities, we performed ChIP-seq experiments using an antibody targeting histone-3 lysine-36 trimethylation (H3K36me3), a histone modification found in gene bodies that is correlated with expression [62, 63]. We ran H3K36me3 ChIP-seq experiments under control culture conditions (DMSO) and after 48 h of GW3965 treatment. We confirmed the predictive value of H3K36me3 gene body occupancy for measuring global gene expression (rho > 0.77; Additional file 1: Figure S36) and subsequently compared H3K36me3 gene body read depth with expression of GW3965-responsive genes (Fig. 6d; Additional file 1: Figures S37–S39). For 78 % of repressed genes at 48 h, H3K36me3 displayed a concordant decrease in gene body occupancy (Fig. 6d; Additional file 1: Figure S37). This pattern was further maintained using lower fold change cutoffs (Additional file 1: Figures S38 and S39). To investigate whether H3K36me3 changes were directly related to LXRs, we evaluated dynamic H3K36me3 occupancy at all 48-h repressed genes that were enriched for nearby LXR binding events (<10 kb from promoter) and for genes that were devoid of nearby LXR binding (>100 kb from promoters). Intriguingly, genes with nearby LXRA and LXRB binding exhibited a significantly stronger decrease (Wilcoxon; LXRA *p* = 1.265e-8, LXRB *p* = 1.394e-9) in H3K36me3 gene body occupancy (Fig. 6e, f). Incorporating GW3965-responsive genes with lower fold change cutoffs led to similarly significant patterns (Wilcoxon; LXRA and LXRB *p* < 2.2e-16) for both proteins (Additional file 1: Figure S40). Taken together, these data are consistent with a RNAP2 promoter-pausing mechanism mediated by LXRA and LXRB nuclear receptors.

## Discussion

Although gene regulatory networks govern genome function and underlie diverse physiological and developmental processes, our overall understanding of how these transcriptional programs are spatiotemporally regulated remains rudimentary. Here we sought to understand the gene regulatory networks that control tumor cell metabolism and proliferation in a temporal manner by generating a thorough characterization of the gene regulatory effects of LXR and PPARG signaling. Collectively, our work illustrates how two distinct gene regulatory cascades lead to a common phenotypic outcome and further provides a better molecular understanding of how extracellular metabolic cues impact tumor cell physiology.

Multiple studies have suggested a role for LXR and PPARG metabolic nuclear receptors in tumor cell biology [64–67]. Our identification of a pronounced inhibitory effect on tumor cell proliferation and decreased glutathione levels that is consistent with increased oxidative stress agrees with previous reports [31, 32]. A recent study suggested increased reactive oxygen species via PDK4 expression and beta-oxidation of fatty acids as the likely anti-proliferative mechanism in lung cancer cells [31]. Indeed, rosiglitazone treatment in our study also led to a significant up-regulation of *PDK4* expression (fold change = 6, adjusted *p* value 2.09e-151). Our metabolomic interrogation further identified an increase in aerobic glycolysis, demonstrating a role for this regulatory network in the Warburg effect. Although this observation is contrary to the observed increase in oxidative stress, the assessment of metabolite concentrations cannot distinguish cause from effect and, therefore, the increase in aerobic glycolysis may reflect an indirect cellular response to oxidative stress rather than a direct effect stemming from LXR and PPARG signaling.

Through an investigation that interrogated multiple tiers of transcriptional regulation and genome organization, we demonstrated that these overlapping cell phenotypes are mediated though widely distinct signaling cascades at the molecular level. The genome-wide cellular response to rosiglitazone was rapid and supported by quick changes to metabolite levels, linear transcriptional profiles, abrupt chromatin alterations, and a short-lived burst of PPARG binding. By contrast, the more gradual changes to cellular metabolites, a stalled transcriptional response, gradual increases in the chromatin landscape, and dramatic increase in genome-wide LXRB occupancy with time highlight a more sustained GW3965 response.

Despite these differing molecular networks, both sets of nuclear receptors were strongly associated with AP1 transcriptional regulators, with PPARG displaying an early preference for AP1 motifs and co-occupancy, while LXRs exhibited a more pronounced late association with AP1 motifs and proteins. In light of the consistent association with upregulated genes, PPARG appears to behave as a transcriptional activator. This is consistent with the canonical model of type II nuclear receptor function, wherein ligand binding leads to the displacement of co-repressor complexes from nuclear receptors and subsequent recruitment of co-activator proteins [68]. The regulatory functions of LXRA and LXRB were more complex; our genomic data points to both activating and repressive activities at early and late time points, respectively. Taken together, our results further point to LXR-mediated genetic repression through RNAP2 promoter-proximal pausing. Unlike the canonical model for PPARG activity, our results suggest the intriguing possibility that these transcriptional profiles reflect a LXR transrepression of the AP1 signaling program through protein–protein interactions at AP1 binding sites that lead to RNAP2 promoter-proximal pausing. Similar mechanisms have been reported for LXRs in macrophages on inflammatory Toll-like receptor genes [69, 70], including transrepression of the AP1 machinery [71]. Further experimentation will be required to fully define the molecular mechanism of this putative LXR-mediated repression.

## Conclusions

By relying on a unique, multi-tiered approach, our detailed results provide a molecular understanding of how extracellular nutrients impact cancer cell physiology at the genome level. This comprehensive analysis illustrates the complexity of genome function and structure by elucidating how common phenotypic outcomes are genetically encoded through diverse transcriptional programs. Our results finally allude to the tantalizing possibility that tumor cell growth can be altered, or even fully inhibited, through metabolic reprogramming.

## Abbreviations

FBS, fetal bovine serum; KEGG, Kyoto Encyclopedia of Genes and Genomes; LXR, liver X receptor; LXRA, liver X receptor alpha; LXRB, liver X receptor beta; PPARG, peroxisome proliferator-activated receptor gamma; RNAP2, RNA polymerase II; TF, transcription factor.

## Additional file

Additional file 1: Figures S1–S40. All supplemental figures with captions. (PDF 23001 kb) Additional file 2: Table S1. (PDF 275 kb) Additional file 3: Table S2. (PDF 71 kb)

## References

[1] Macneil LT, Walhout AJ (2011). Gene regulatory networks and the role of robustness and stochasticity in the control of gene expression. Genome Res.

[2] Herschman HR (1989). Extracellular signals, transcriptional responses and cellular specificity. Trends Biochem Sci.

[3] Lopez-Maury L, Marguerat S, Bahler J (2008). Tuning gene expression to changing environments: from rapid responses to evolutionary adaptation. Nat Rev Genet.

[4] Bulger M, Groudine M (2011). Functional and mechanistic diversity of distal transcription enhancers. Cell.

[5] Sakabe NJ, Savic D, Nobrega MA (2012). Transcriptional enhancers in development and disease. Genome Biol.

[6] Levine M (2010). Transcriptional enhancers in animal development and evolution. Curr Biol.

[7] Tjian R, Maniatis T (1994). Transcriptional activation: a complex puzzle with few easy pieces. Cell.

[8] Calkin AC, Tontonoz P (2012). Transcriptional integration of metabolism by the nuclear sterol-activated receptors LXR and FXR. Nat Rev Mol Cell Biol.

[9] Berger J, Moller DE (2002). The mechanisms of action of PPARs. Annu Rev Med..

[10] Joseph SB, Laffitte BA, Patel PH, Watson MA, Matsukuma KE, Walczak R, Collins JL, Osborne TF, Tontonoz P. Direct and indirect mechanisms for regulation of fatty acid synthase gene expression by liver X receptors. J Biol Chem. 2002;277(13):11019–25. 10.1074/jbc.M111041200 11790787

[11] Li AC, Glass CK (2004). PPAR- and LXR-dependent pathways controlling lipid metabolism and the development of atherosclerosis. J Lipid Res.

[12] Lehmann JM, Moore LB, Smith-Oliver TA, Wilkison WO, Willson TM, Kliewer SA (1995). An antidiabetic thiazolidinedione is a high affinity ligand for peroxisome proliferator-activated receptor gamma (PPAR gamma). J Biol Chem.

[13] Mitro N, Mak PA, Vargas L, Godio C, Hampton E, Molteni V, Kreusch A, Saez E. The nuclear receptor LXR is a glucose sensor. Nature. 2007;445(7124):219–23. 10.1038/nature05449 17187055

[14] Hong C, Tontonoz P (2008). Coordination of inflammation and metabolism by PPAR and LXR nuclear receptors. Curr Opin Genet Dev.

[15] Jaziri R, Lobbens S, Aubert R, Pean F, Lahmidi S, Vaxillaire M, Porchay I, Bellili N, Tichet J, Balkau B et al. The PPARG Pro12Ala polymorphism is associated with a decreased risk of developing hyperglycemia over 6 years and combines with the effect of the APM1 G-11391A single nucleotide polymorphism: the Data From an Epidemiological Study on the Insulin Resistance Syndrome (DESIR) study. Diabetes. 2006;55(4):1157–62. 10.2337/diabetes.55.04.06.db05-0676 16567542

[16] Rangwala SM, Lazar MA (2004). Peroxisome proliferator-activated receptor gamma in diabetes and metabolism. Trends Pharmacol Sci.

[17] Cao G, Liang Y, Broderick CL, Oldham BA, Beyer TP, Schmidt RJ, Zhang Y, Stayrook KR, Suen C, Otto KA et al. Antidiabetic action of a liver x receptor agonist mediated by inhibition of hepatic gluconeogenesis. J Biol Chem. 2003;278(2):1131–6. 10.1074/jbc.M210208200 12414791

[18] Steffensen KR, Gustafsson JA (2004). Putative metabolic effects of the liver X receptor (LXR). Diabetes..

[19] Laffitte BA, Chao LC, Li J, Walczak R, Hummasti S, Joseph SB, Castrillo A, Wilpitz DC, Mangelsdorf DJ, Collins JL et al. Activation of liver X receptor improves glucose tolerance through coordinate regulation of glucose metabolism in liver and adipose tissue. Proc Natl Acad Sci U S A. 2003;100(9):5419–24. 10.1073/pnas.0830671100 PMC154360 12697904

[20] Kliewer SA, Lenhard JM, Willson TM, Patel I, Morris DC, Lehmann JM (1995). A prostaglandin J2 metabolite binds peroxisome proliferator-activated receptor gamma and promotes adipocyte differentiation. Cell.

[21] Hu E, Kim JB, Sarraf P, Spiegelman BM (1996). Inhibition of adipogenesis through MAP kinase-mediated phosphorylation of PPARgamma. Science.

[22] Walczak R, Tontonoz P (2002). PPARadigms and PPARadoxes: expanding roles for PPARgamma in the control of lipid metabolism. J Lipid Res.

[23] Gao M, Liu D (2013). The liver X receptor agonist T0901317 protects mice from high fat diet-induced obesity and insulin resistance. AAPS J.

[24] Kalaany NY, Gauthier KC, Zavacki AM, Mammen PP, Kitazume T, Peterson JA, Horton JD, Garry DJ, Bianco AC, Mangelsdorf DJ. LXRs regulate the balance between fat storage and oxidation. Cell Metab. 2005;1(4):231–44. 10.1016/j.cmet.2005.03.001 16054068

[25] Feng J, Han J, Pearce SF, Silverstein RL, Gotto Jr AM, Hajjar DP, Nicholson AC. Induction of CD36 expression by oxidized LDL and IL-4 by a common signaling pathway dependent on protein kinase C and PPAR-gamma. J Lipid Res. 2000;41(5):688–96. 10787429

[26] Collins AR, Meehan WP, Kintscher U, Jackson S, Wakino S, Noh G, Palinski W, Hsueh WA, Law RE. Troglitazone inhibits formation of early atherosclerotic lesions in diabetic and nondiabetic low density lipoprotein receptor-deficient mice. Arterioscler Thromb Vasc Biol. 2001;21(3):365–71. 10.1161/01.atv.21.3.365 11231915

[27] Chen Z, Ishibashi S, Perrey S, Osuga J, Gotoda T, Kitamine T, Tamura Y, Okazaki H, Yahagi N, Iizuka Y et al. Troglitazone inhibits atherosclerosis in apolipoprotein E-knockout mice: pleiotropic effects on CD36 expression and HDL. Arterioscler Thromb Vasc Biol. 2001;21(3):372–7. 10.1161/01.atv.21.3.372 11231916

[28] Joseph SB, McKilligin E, Pei L, Watson MA, Collins AR, Laffitte BA, Chen M, Noh G, Goodman J, Hagger GN et al. Synthetic LXR ligand inhibits the development of atherosclerosis in mice. Proc Natl Acad Sci U S A. 2002;99(11):7604–9. 10.1073/pnas.112059299 PMC124297 12032330

[29] Bradley MN, Hong C, Chen M, Joseph SB, Wilpitz DC, Wang X, Lusis AJ, Collins A, Hseuh WA, Collins JL et al. Ligand activation of LXR beta reverses atherosclerosis and cellular cholesterol overload in mice lacking LXR alpha and apoE. J Clin Invest. 2007;117(8):2337–46. 10.1172/JCI31909 PMC1924496 17657314

[30] Saez E, Tontonoz P, Nelson MC, Alvarez JG, Ming UT, Baird SM, Thomazy VA, Evans RM. Activators of the nuclear receptor PPARgamma enhance colon polyp formation. Nat Med. 1998;4(9):1058–61. 10.1038/2042 9734400

[31] Srivastava N, Kollipara RK, Singh DK, Sudderth J, Hu Z, Nguyen H, Humphries CG, Carstens R, Huffman KE. Inhibition of cancer cell proliferation by PPARgamma is mediated by a metabolic switch that increases reactive oxygen species levels. Cell Metab. 2014;20(4):650–61. 10.1016/j.cmet.2014.08.003 PMC4191999 25264247

[32] Vedin LL, Gustafsson JA, Steffensen KR. The oxysterol receptors LXRalpha and LXRbeta suppress proliferation in the colon. Mol Carcinog. 2013;52(11):835-44. 10.1002/mc.21924 22610535

[33] Fukuchi J, Kokontis JM, Hiipakka RA, Chuu CP, Liao S (2004). Antiproliferative effect of liver X receptor agonists on LNCaP human prostate cancer cells. Cancer Res.

[34] Plat J, Nichols JA, Mensink RP (2005). Plant sterols and stanols: effects on mixed micellar composition and LXR (target gene) activation. J Lipid Res.

[35] Ronco A, De Stefani E, Boffetta P, Deneo-Pellegrini H, Mendilaharsu M, Leborgne F (1999). Vegetables, fruits, and related nutrients and risk of breast cancer: a case-control study in Uruguay. Nutr Cancer.

[36] De Stefani E, Boffetta P, Ronco AL, Brennan P, Deneo-Pellegrini H, Carzoglio JC, Mendilaharsu M. Plant sterols and risk of stomach cancer: a case-control study in Uruguay. Nutr Cancer. 2000;37(2):140–4. 10.1207/S15327914NC372_4 11142085

[37] Mendilaharsu M, De Stefani E, Deneo-Pellegrini H, Carzoglio J, Ronco A (1998). Phytosterols and risk of lung cancer: a case-control study in Uruguay. Lung Cancer.

[38] Baskar AA, Ignacimuthu S, Paulraj GM, Al Numair KS (2010). Chemopreventive potential of beta-Sitosterol in experimental colon cancer model--an in vitro and In vivo study. BMC Complement Altern Med..

[39] Chuu CP, Hiipakka RA, Kokontis JM, Fukuchi J, Chen RY, Liao S (2006). Inhibition of tumor growth and progression of LNCaP prostate cancer cells in athymic mice by androgen and liver X receptor agonist. Cancer Res.

[40] Gabbi C, Kim HJ, Barros R, Korach-Andre M, Warner M, Gustafsson JA (2010). Estrogen-dependent gallbladder carcinogenesis in LXRbeta-/- female mice. Proc Natl Acad Sci U S A.

[41] Vedin LL, Lewandowski SA, Parini P, Gustafsson JA, Steffensen KR (2009). The oxysterol receptor LXR inhibits proliferation of human breast cancer cells. Carcinogenesis.

[42] Pommier AJ, Alves G, Viennois E, Bernard S, Communal Y, Sion B, Marceau G, Damon C, Mouzat K, Caira F et al. Liver X Receptor activation downregulates AKT survival signaling in lipid rafts and induces apoptosis of prostate cancer cells. Oncogene. 2010;29(18):2712–23. 10.1038/onc.2010.30 20190811

[43] Lee JH, Gong H, Khadem S, Lu Y, Gao X, Li S, Zhang J, Xie W. Androgen deprivation by activating the liver X receptor. Endocrinology. 2008;149(8):3778–88. 10.1210/en.2007-1605 PMC2488233 18450964

[44] Kotta-Loizou I, Giaginis C, Theocharis S (2012). The role of peroxisome proliferator-activated receptor-gamma in breast cancer. Anticancer Agents Med Chem.

[45] Pang X, Wei Y, Zhang Y, Zhang M, Lu Y, Shen P (2013). Peroxisome proliferator-activated receptor-gamma activation inhibits hepatocellular carcinoma cell invasion by upregulating plasminogen activator inhibitor-1. Cancer Sci.

[46] Girnun GD, Smith WM, Drori S, Sarraf P, Mueller E, Eng C, Nambiar P, Rosenberg DW, Bronson RT, Edelmann W et al. APC-dependent suppression of colon carcinogenesis by PPARgamma. Proc Natl Acad Sci U S A. 2002;99(21):13771–6. 10.1073/pnas.162480299 PMC129773 12370429

[47] Jansson EA, Are A, Greicius G, Kuo IC, Kelly D, Arulampalam V, Pettersson S. The Wnt/beta-catenin signaling pathway targets PPARgamma activity in colon cancer cells. Proc Natl Acad Sci U S A. 2005;102(5):1460–5. 10.1073/pnas.0405928102 PMC547827 15665104

[48] Dunn WB, Broadhurst D, Begley P, Zelena E, Francis-McIntyre S, Anderson N, Brown M, Knowles JD, Halsall A, Haselden JN (2011). Procedures for large-scale metabolic profiling of serum and plasma using gas chromatography and liquid chromatography coupled to mass spectrometry. Nat Protoc.

[49] Gertz J, Savic D, Varley KE, Partridge EC, Safi A, Jain P, Cooper GM, Reddy TE, Crawford GE, Myers RM. Distinct properties of cell-type-specific and shared transcription factor binding sites. Mol Cell. 2013;52(1):25–36. 10.1016/j.molcel.2013.08.037 PMC3811135 24076218

[50] Gertz J, Varley KE, Davis NS, Baas BJ, Goryshin IY, Vaidyanathan R, Kuersten S, Myers RM. Transposase mediated construction of RNA-seq libraries. Genome Res. 2012;22(1):134–41. 10.1101/gr.127373.111 PMC3246200 22128135

[51] Zhang Y, Liu T, Meyer CA, Eeckhoute J, Johnson DS, Bernstein BE, Nusbaum C, Myers RM, Brown M, Li W. Model-based analysis of ChIP-Seq (MACS). Genome Biol. 2008;9(9):R137. 10.1186/gb-2008-9-9-r137 PMC2592715 18798982

[52] Bailey TL, Williams N, Misleh C, Li WW (2006). MEME: discovering and analyzing DNA and protein sequence motifs. Nucleic Acids Res.

[53] Merico D, Isserlin R, Stueker O, Emili A, Bader GD (2010). Enrichment map: a network-based method for gene-set enrichment visualization and interpretation. PLoS One.

[54] Anders S, Huber W (2010). Differential expression analysis for sequence count data. Genome Biol.

[55] Subramanian A, Tamayo P, Mootha VK, Mukherjee S, Ebert BL, Gillette MA, Paulovich A, Pomeroy SL, Golub TR, Lander ES. Gene set enrichment analysis: a knowledge-based approach for interpreting genome-wide expression profiles. Proc Natl Acad Sci U S A. 2005;102(43):15545–50. 10.1073/pnas.0506580102 PMC1239896 16199517

[56] Creyghton MP, Cheng AW, Welstead GG, Kooistra T, Carey BW, Steine EJ, Hanna J, Lodato MA, Frampton GM, Sharp PA et al. Histone H3K27ac separates active from poised enhancers and predicts developmental state. Proc Natl Acad Sci U S A. 2010;107(50):21931–6. 10.1073/pnas.1016071107 PMC3003124 21106759

[57] McLean CY, Bristor D, Hiller M, Clarke SL, Schaar BT, Lowe CB, Wenger AM, Bejerano G. GREAT improves functional interpretation of cis-regulatory regions. Nat Biotechnol. 2010;28(5):495–501. 10.1038/nbt.1630 PMC4840234 20436461

[58] Angel P, Karin M (1991). The role of Jun, Fos and the AP-1 complex in cell-proliferation and transformation. Biochim Biophys Acta.

[59] Biddie SC, John S, Sabo PJ, Thurman RE, Johnson TA, Schiltz RL, Miranda TB, Sung MH, Trump S, Lightman SL. Transcription factor AP1 potentiates chromatin accessibility and glucocorticoid receptor binding. Mol Cell. 2011;43(1):145–55. 10.1016/j.molcel.2011.06.016 PMC3138120 21726817

[60] Savic D, Roberts BS, Carleton JB, Partridge EC, White MA, Cohen BA, Cooper GM, Gertz J, Myers RM: Promoter-distal RNA polymerase II binding discriminates active from inactive CCAAT/enhancer-binding protein beta binding sites. Genome Res. 2015;25(12):1791–800. 10.1101/gr.191593.115 PMC4665001 26486725

[61] De Santa F, Barozzi I, Mietton F, Ghisletti S, Polletti S, Tusi BK, Muller H, Ragoussis J, Wei CL, Natoli G. A large fraction of extragenic RNA pol II transcription sites overlap enhancers. PLoS Biol. 2010;8(5):e1000384. 10.1371/journal.pbio.1000384 PMC2867938 20485488

[62] Dong X, Greven MC, Kundaje A, Djebali S, Brown JB, Cheng C, Gingeras TR, Gerstein M, Guigo R, Birney E et al. Modeling gene expression using chromatin features in various cellular contexts. Genome Biol. 2012;13(9):R53. 10.1186/gb-2012-13-9-r53 PMC3491397 22950368

[63] Kolasinska-Zwierz P, Down T, Latorre I, Liu T, Liu XS, Ahringer J (2009). Differential chromatin marking of introns and expressed exons by H3K36me3. Nat Genet.

[64] Bovenga F, Sabba C, Moschetta A (2015). Uncoupling nuclear receptor LXR and cholesterol metabolism in cancer. Cell Metab.

[65] Lin CY, Gustafsson JA (2015). Targeting liver X receptors in cancer therapeutics. Nat Rev Cancer.

[66] Joshi H, Pal T, Ramaa CS (2014). A new dawn for the use of thiazolidinediones in cancer therapy. Expert Opin Investig Drugs.

[67] Zhang Z, Xu Y, Xu Q, Hou Y (2013). PPARgamma against tumors by different signaling pathways. Onkologie.

[68] Sever R, Glass CK (2013). Signaling by nuclear receptors. Cold Spring Harb Perspect Biol.

[69] Ghisletti S, Huang W, Ogawa S, Pascual G, Lin ME, Willson TM, Rosenfeld MG, Glass CK. Parallel SUMOylation-dependent pathways mediate gene- and signal-specific transrepression by LXRs and PPARgamma. Mol Cell. 2007;25(1):57–70. 10.1016/j.molcel.2006.11.022 PMC1850387 17218271

[70] Huang W, Ghisletti S, Saijo K, Gandhi M, Aouadi M, Tesz GJ, Zhang DX, Yao J, Czech MP, Goode BL et al. Coronin 2A mediates actin-dependent de-repression of inflammatory response genes. Nature. 2011;470(7334):414–8. 10.1038/nature09703 PMC3464905 21331046

[71] Ogawa S, Lozach J, Jepsen K, Sawka-Verhelle D, Perissi V, Sasik R, Rose DW, Johnson RS, Rosenfeld MG, Glass CK. A nuclear receptor corepressor transcriptional checkpoint controlling activator protein 1-dependent gene networks required for macrophage activation. Proc Natl Acad Sci U S A. 2004;101(40):14461–6. 10.1073/pnas.0405786101 PMC521940 15452344

