# Supplementary material for: Distinct gene regulatory programs define the inhibitory effects of liver X receptors and PPARG on cancer cell proliferation
Source: Genome Med. 2016 Jul 11;8:74. doi: 10.1186/s13073-016-0328-6 (PMC4940857; doi:10.1186/s13073-016-0328-6)
Supplement: Additional file 1: Figures S1–S40. — All supplemental figures with captions. (PDF 23001 kb) [file 13073_2016_328_MOESM1_ESM.pdf]

SUPPLEMENTAL FIGURES

Distinct gene regulatory programs define the inhibitory effects of LXRs and PPARG on cancer cell proliferation

Daniel Savic[1], Ryne C. Ramaker[1,2], Brian S. Roberts[1], Todd C. Burwell[1], Emma C. Dean[1], Sarah K. Meadows[1], Sara J. Cooper[1], Michael J. Garabedian[3], Jason Gertz[4] and Richard M. Myers[1]

(1) HudsonAlpha Institute for Biotechnology, Huntsville, AL 35806

(2) Department of Genetics, University of Alabama at Birmingham, Birmingham, AL, 35294

(3) Departments of Microbiology and Urology, New York University, New York, NY, 10016

(4) Department of Oncological Sciences, Huntsman Cancer Institute, University of Utah, Salt Lake City, UT, 84112

TABLE OF CONTENTS

Table of Contents……………………………………………………………….Page 2
Supplemental Figure Legends…………………………………………………Page 3-14
Figure S1..………………………………………………………………….....Page 15
Figure S2…………………………………………………………………………Page 16
Figure S3…………………………………………………………………………Page 17
Figure S4…………………………………………………………………………Page 18
Figure S5…………………………………………………………………………Page 19
Figure S6…………………………………………………………………………Page 20
Figure S7…………………………………………………………………………Page 21
Figure S8…………………………………………………………………………Page 22
Figure S9…………………………………………………………………………Page 23
Figure S10……………………………………………………………………….Page 24
Figure S11……………………………………………………………………….Page 25
Figure S12……………………………………………………………………….Page 26
Figure S13……………………………………………………………………….Page 27
Figure S14……………………………………………………………………….Page 28
Figure S15……………………………………………………………………….Page 29
Figure S16……………………………………………………………………….Page 30
Figure S17……………………………………………………………………….Page 31
Figure S18……………………………………………………………………….Page 32
Figure S19……………………………………………………………………….Page 33
Figure S20……………………………………………………………………….Page 34
Figure S21……………………………………………………………………….Page 35
Figure S22……………………………………………………………………….Page 36
Figure S23……………………………………………………………………….Page 37
Figure S24……………………………………………………………………….Page 38
Figure S25……………………………………………………………………….Page 39
Figure S26……………………………………………………………………….Page 40
Figure S27……………………………………………………………………….Page 41
Figure S28……………………………………………………………………….Page 42
Figure S29……………………………………………………………………….Page 43
Figure S30……………………………………………………………………….Page 44
Figure S31……………………………………………………………………….Page 45
Figure S32……………………………………………………………………….Page 46
Figure S33……………………………………………………………………….Page 47
Figure S34……………………………………………………………………….Page 48
Figure S35……………………………………………………………………….Page 49
Figure S36……………………………………………………………………….Page 50
Figure S37……………………………………………………………………….Page 51
Figure S38……………………………………………………………………….Page 52
Figure S39……………………………………………………………………….Page 53
Figure S40……………………………………………………………………….Page 54

SUPPLEMENTAL FIGURE LEGENDS

Figure S1. Additional metabolite levels after drug treatment

Relative metabolite abundance after 24 (left) and 48 (right) hours of GW3965 (light blue), rosiglitazone (gold) and DMSO (gray) cell treatments for a variety of cellular metabolites. * $p<0.05$.

Figure S2. Comparison of GW3965 and T0901317 agonists

(A) Comparisons of fold changes between genes significantly regulated (adjusted $p<0.05$) by both GW3965 (y-axis) and T0901317 (x-axis). The rank correlation is given at the bottom right of the plot. **(B)** The number of differentially regulated genes after 24 and 48 hours of drug treatment (adjusted $p<0.01$, fold change cutoff of +/-2). Plots are shown for rosiglitazone and a combination of GW3965 and T0901317 (GW & T0) treatments (adjusted $p<0.01$, fold change cutoff of +/-2 for both GW3965 and T0901317 treatments).

Figure S3. Differentially regulated genes at a fold change cutoff of +/-1.5

The number of differentially regulated genes after 24 and 48 hours of drug treatment (adjusted $p<0.01$ and fold change cutoff of +/-1.5) is given.

Figure S4. Gene ontology connectivity map for rosiglitazone

(A) Gene Ontology connectivity map for rosiglitazone treatment at 24 hours for up-regulated (red) and down-regulated (red) pathways. **(B)** Gene Ontology connectivity map for rosiglitazone treatment at 48 hours for up-regulated (red) and down-regulated (red) pathways.

Figure S5. H3K27ac sites gained and lost

(A) The number of H3K27ac sites gained (y-axis) after 24 and 48 hours of GW3965 (blue) and rosiglitazone (gold) treatment (x-axis). **(B)** The number of H3K27ac sites lost (y-axis) after 24 and 48 hours of GW3965 (blue) and rosiglitazone (gold) treatment (x-axis).

Figure S6. Common H3K27ac sites between ligand treatments and pathway enrichments

All GW3965 and rosiglitazone H3K27ac sites gained (compared to control DMSO conditions) were used for this analysis. Venn diagrams display overlap (in gray) between GW3965-gained (in blue) and rosiglitazone-gained (in gold) sites after ligand treatment after 24 hours (A) and after 48 hours (B). Pathway enrichments using GREAT for common H3K27ac sites gained across both drug treatments are shown at 24 hours (C) and 48 hours (D).

Figure S7. Dynamic changes to H3K27ac occupancy

(A) Heatmap denotes individual binding sites (y-axis) that exhibited changes in H3K27ac occupancy from control culture conditions (blue, DMSO, x-axis) to 24 hours of GW3965 treatment (green, 24hr, x-axis), and from 24 hours of GW3965 treatment to 48 hours of GW3965 treatment (red, 48hr, x-axis). **(B)** Heatmap denotes individual binding sites (y-axis) that exhibited changes in H3K27ac occupancy from baseline (blue DMSO, x-axis) to 24 hours of rosiglitazone treatment (green, 24hr, x-axis), and from 24 hours of GW3965 treatment to 48 hours of rosiglitazone treatment (red, 48hr, x-axis). For all heatmaps, weaker occupancies are shown in blue while stronger enrichment is given in yellow. Intermediate enrichments are given in black.

Figure S8. Enrichment of sites with increasing H3K27ac occupancy near differentially regulated genes

(A) Cumulative distribution functions display the fraction of target gene promoters (y-axis) at different distance cutoffs to the nearest H3K27ac binding event (x-axis). Plots for the promoters of GW3965 responsive up-regulated genes (blue) and down-regulated (red) genes (adjusted $p<0.01$, fold change cutoff of +/-2) are shown. The background distribution using all gene promoters in the genome is also displayed (BKG; black). **(B)** Cumulative distribution functions display the fraction of target gene promoters (y-axis) at different distance cutoffs to the nearest H3K27ac binding event (x-axis). Plots for the promoters of rosiglitazone responsive up-regulated genes (blue) and down-regulated (red) genes (adjusted $p<0.01$, fold change cutoff of +/-2) are shown. The background distribution using all gene promoters in the genome is also displayed (BKG; black).

Figure S9. Enrichment of sites with increasing H3K27ac occupancy near differentially regulated high-confidence LXR genes

Cumulative distribution functions display the fraction of target gene promoters (y-axis) at different distance cutoffs to the nearest H3K27ac binding event (x-axis). Plots for the promoters of GW3965+T0901317 responsive up-regulated genes (blue) and down-regulated (red) genes (adjusted $p<0.01$, fold change cutoff of +/-2 for both GW3965 and T0901317 drug treatments) are shown. The background distribution using all gene promoters in the genome is also displayed (BKG; black).

Figure S10. Pathway analysis of H3K27ac sites exhibiting increased temporal occupancy during GW3965 treatment

(A) Significant Gene Ontology Biological Process pathway analysis using GREAT for H3K27ac sites that exhibited increasingly stronger enrichment during GW3965 drug treatments. **(B)** Significant Gene Ontology Disease Ontology pathway analysis using GREAT for H3K27ac sites that exhibited increasingly stronger enrichment during GW3965 drug treatments.

Figure S11. Pathway analysis of H3K27ac sites exhibiting increased temporal occupancy during rosiglitazone treatment

(A) Significant Gene Ontology Biological Process pathway analysis using GREAT for H3K27ac sites that exhibited increasingly stronger enrichment during rosiglitazone drug treatments. **(B)** Significant Gene Ontology Disease Ontology pathway analysis using GREAT for H3K27ac sites that exhibited increasingly stronger enrichment during rosiglitazone drug treatments.

Figure S12. AP1 motif enrichment and JunD co-occupancy at LXRA sites

(A) The fold enrichment of various canonical motifs for the top 50% of ranked LXRA binding sites exhibiting stronger enrichment at 2 hours (light green) or 48 hours (dark green) of GW3965 treatment. **(B)** The percentage of JunD sites overlapping with LXRA nuclear receptor (NR) binding events after 2 and 48 hours of GW3965 treatment. Sites overlapping with nuclear receptors are in blue while sites not overlapping nuclear receptors are in red. **(C)** The percentage of LXRA sites after 2 and 48 hours of stimulation coincident with JunD binding.

Figure S13. Integration of nuclear receptor binding data with drug responsive genes (fold change cutoff of +/-1.5)

Cumulative distribution functions display the fraction of target gene promoters (y-axis) at different distance cutoffs to the nearest LXRA, LXRB and PPARG binding event (x-axis). Plots for the promoters of drug responsive up-regulated genes (green) and down-regulated genes (red) are shown (adjusted $p<0.01$, fold change cutoff of +/-1.5). The background distribution using all gene promoters in the genome is also displayed (Bkgd; black). The left panel represents 2-hour ChIP-seq data compared to 24-hour RNA-seq data while the right panel compares 48-hour ChIP-seq and RNA-seq data. For LXR analyses, only high-confidence GW3965+T0901317 responsive genes differentially regulated by both GW3965 and T0901317 were used. Down-regulated curves are absent for 24-hour LXR functions as only five genes were identified.

Figure S14. Integration of LXR binding data with 48-hour GW3965+T0901317 repressed genes not up-regulated at 24 hours

Cumulative distribution functions display the fraction of target gene promoters (y-axis) at different distance cutoffs to the nearest LXRA and LXRB binding event (x-axis). Plots for the promoters of GW3965+T0901317 repressed genes (adjusted p<0.01) are shown. ChIP-seq and RNA-seq data at 48 hours were used. Plots of all 48-hour GW3965+T0901317 down-regulated genes (red), as well as 48-hour repressed genes that were not up-regulated (adjusted p<0.05) at 24 hours (dark red) are shown. The background distribution using all gene promoters in the genome is also displayed (Bkgd; black). The top panel uses RNA-seq data using a fold change cutoff of +/-1.5 (for both GW3965 and T0901317) while the bottom panel uses a fold change cutoff of +/-2 (for both GW3965 and T0901317).

Figure S15. Integration of LXR binding data with GW3965 responsive genes (fold change cutoff of +/-2)

Cumulative distribution functions display the fraction of target gene promoters (y-axis) at different distance cutoffs to the nearest LXRA and LXRB binding event (x-axis). Plots for the promoters of GW3965 up-regulated genes (green) and repressed genes (red) are shown (adjusted p<0.01, fold change cutoff of +/2). The background distribution using all gene promoters in the genome is also displayed (Bkgd; black). The top panel represents 2-hour ChIP-seq data compared to 24-hour RNA-seq data while the bottom panel compares 48-hour ChIP-seq and RNA-seq data.

Figure S16. Integration of LXR binding data with GW3965 responsive genes (fold change cutoff of +/-1.5)

Cumulative distribution functions display the fraction of target gene promoters (y-axis) at different distance cutoffs to the nearest LXRA and LXRB binding event (x-axis). Plots for the promoters of GW3965 up-regulated genes (green) and repressed genes (red) are shown (adjusted p<0.01, fold change cutoff of +/1.5). The background distribution using all gene promoters in the genome is also displayed (Bkgd; black). The top panel represents 2-hour ChIP-seq data compared to 24-hour RNA-seq data while the bottom panel compares 48-hour ChIP-seq and RNA-seq data.

Figure S17. Integration of LXR binding data with 48-hour GW3965 repressed genes not up-regulated at 24 hours

Cumulative distribution functions display the fraction of target gene promoters (y-axis) at different distance cutoffs to the nearest LXRA and LXRB binding event (x-axis). Plots for the promoters of

GW3965 repressed genes (adjusted p<0.01) are shown. ChIP-seq and RNA-seq data at 48 hours were used. Plots using all down-regulated genes at 48 hours (red) as well as 48-hour repressed genes that were not up-regulated (adjusted p<0.05) at 24 hours (dark red) are shown. The background distribution using all gene promoters in the genome is also displayed (Bkgd; black). The top panel uses RNA-seq data using a fold change of +/-1.5 while the bottom panel uses a fold change of +/-2.

Figure S18. Integration of RNAP2 and LXR binding site co-occupancy with 48-hour GW3965+T0901317 repressed genes not up-regulated at 24 hours

Cumulative distribution functions display the fraction of target gene promoters (y-axis) at different distance cutoffs to the nearest LXRA and LXRB binding event (x-axis). Plots for the promoters of 48-hour GW3965+T0901317 repressed genes (adjusted p<0.01, fold change cutoff of -2 for both GW3965 and T0901317 treatments) are shown. ChIP-seq and RNA-seq data at 48 hours were used. All 48-hour repressed genes that were not up-regulated (adjusted p<0.05) were utilized. LXRA binding events coincident with RNAP2 are in dark purple (+RNAP2), while LXRA sites devoid of RNAP2 occupancy are denoted in pink (-RNAP2). The background distribution using all gene promoters in the genome for sites overlapping with RNAP2 (+RNAP2 Bkgd; black) or for sites devoid of RNAP2 (-RNAP2 Bkgd; gray) is also graphed.

Figure S19. Integration of RNAP2 and LXR binding site co-occupancy with 48-hour GW3965 repressed genes not up-regulated at 24 hours

Cumulative distribution functions display the fraction of target gene promoters (y-axis) at different distance cutoffs to the nearest LXRA and LXRB binding event (x-axis). Plots for the promoters of 48-hour GW3965 repressed genes (adjusted p<0.01, fold change cutoff of -2) at different distances (x-axis) are shown. ChIP-seq and RNA-seq data at 48 hours were used. All 48-hour repressed genes that were not up-regulated (adjusted p<0.05) at 24 hours were utilized. LXRA binding events coincident with RNAP2 are in dark purple (+RNAP2), while LXRA sites devoid of RNAP2 occupancy are denoted in pink (-RNAP2). The background distribution using all gene promoters in the genome for sites overlapping with RNAP2 (+RNAP2 Bkgd; black) or for sites devoid of RNAP2 (-RNAP2 Bkgd; gray) is also graphed.

Figure S20. Integration of RNAP2 and PPARG binding site co-occupancy with 24-hour rosiglitazone up-regulated genes

Cumulative distribution functions display the fraction of target gene promoters (y-axis) at different distance cutoffs to the nearest PPARG binding event (x-axis). Plots for the promoters of 24-hour

rosiglitazone up-regulated genes (adjusted p<0.01, fold change cutoff of 2) are shown. ChIP-seq at 2 hours and RNA-seq data at 24 hours were used. PPARG binding events coincident with RNAP2 are in dark purple (PPARG+RNAP2), while LXRA sites devoid of RNAP2 occupancy are denoted in pink (PPARG-RNAP2). The background distribution using all gene promoters in the genome for sites overlapping with RNAP2 (PPARG+RNAP2 Bkgd; black) or for sites devoid of RNAP2 (PPARG-RNAP2 Bkgd; gray) is also graphed. At the bottom right, the percentage of PPARG binding sites that co-occurred with RNAP2 is shown in the pie chart (green).

Figure S21. Integration of H3K27ac and nuclear receptor binding site co-occupancy with drug responsive genes

(A) Cumulative distribution functions display the fraction of target gene promoters (y-axis) at different distance cutoffs to the nearest LXRA and LXRB binding event (x-axis). Plots for the promoters of 48-hour GW3965+T0901317 repressed genes (adjusted p<0.01, fold change cutoff of -2 for both GW3965 and T0901317 treatments) are shown. ChIP-seq and RNA-seq data at 48 hours were used. LXRA and LXRB binding events coincident with H3K27ac are in dark purple (LXR+H3K27ac), while LXR sites devoid of H3K27ac occupancy are denoted in pink (LXR-H3K27ac). The background distribution using all gene promoters in the genome for sites overlapping with H3K27ac (+H3K27ac Bkgd; black) or for sites devoid of H3K27ac (-H3K27ac Bkgd; gray) is also graphed. **(B)** Cumulative distribution functions display the fraction of target gene promoters (y-axis) at different distance cutoffs to the nearest PPARG binding event (x-axis). Plots for the promoters of 24-hour rosiglitazone up-regulated genes (p<0.01, fold change cutoff of 2) are shown. ChIP-seq data at 2 hours and RNA-seq data at 24 hours were used. PPARG binding events coincident with H3K27ac are in dark purple (PPARG+H3K27ac), while PPARG sites devoid of H3K27ac occupancy are denoted in pink (PPARG-H3K27ac). The background distribution using all gene promoters in the genome for sites overlapping with H3K27ac (+H3K27ac Bkgd; black) or for sites devoid of H3K27ac (-H3K27ac Bkgd; gray) is also graphed.

Figure S22. Integration of H3K27ac and LXR binding site co-occupancy with 48-hour GW3965 repressed genes (fold change cutoff of -2)

Cumulative distribution functions display the fraction of target gene promoters (y-axis) at different distance cutoffs to the nearest LXRA and LXRB binding event (x-axis). Plots for the promoters of 48-hour GW3965 repressed genes (adjusted p<0.01, fold change cutoff of -2) are shown. ChIP-seq and RNA-seq data at 48 hours were used. LXRA and LXRB binding events coincident with H3K27ac are in dark purple (LXR+H3K27ac), while LXR sites devoid of H3K27ac occupancy are denoted in pink (LXR-H3K27ac). The background distribution using all gene promoters in the genome for sites overlapping

with H3K27ac (+H3K27ac Bkgd; black) or for sites devoid of H3K27ac (-H3K27ac Bkgd; gray) is also graphed.

Figure S23. Integration of H3K27ac and LXR binding site co-occupancy with 48-hour GW3965 repressed genes not up-regulated at 24 hours

Cumulative distribution functions display the fraction of target gene promoters (y-axis) at different distance cutoffs to the nearest LXRA and LXRB binding event (x-axis). Plots for the promoters of 48-hour GW3965 repressed genes ($p<0.01$, fold change cutoff of -2) are shown. ChIP-seq and RNA-seq data at 48 hours were used. All 48-hour down-regulated genes that were not up-regulated (adjusted $p<0.05$) at 24 hours were utilized. LXRA and LXRB binding events coincident with H3K27ac are in dark purple (LXR+H3K27ac), while LXR sites devoid of H3K27ac occupancy are denoted in pink (LXR-H3K27ac). The background distribution using all gene promoters in the genome for sites overlapping with H3K27ac (+H3K27ac Bkgd; black) or for sites devoid of H3K27ac (-H3K27ac Bkgd; gray) is also graphed.

Figure S24. Integration of H3K27ac and nuclear receptor binding site co-occupancy with all additional drug responsive gene sets

Cumulative distribution functions (CDFs) display the fraction of target gene promoters (y-axis) at different distance cutoffs to the nearest LXRA (A), LXRB (B) and PPARG (C) binding event (x-axis). Plots for the promoters of 24-hour (first panel) and 48-hour (second panel) GW3965+T0901317 activated genes (adjusted $p<0.01$, fold change cutoff of 2 for both GW3965 and T0901317 treatments) are shown in (A) and (B). For PPARG (C), CDFs using 24-hour rosiglitazone repressed genes (first panel, adjusted $p<0.01$, fold change cutoff of -2), 48-hour rosiglitazone activated genes (second panel, adjusted $p<0.01$, fold change cutoff of 2) and 48-hour rosiglitazone repressed genes (third panel, adjusted $p<0.01$, fold change cutoff of -2) are shown.

Figure S25. Integration of JunD and LXR binding site co-occupancy with 48-hour GW3965+T0901317 repressed genes

Cumulative distribution functions display the fraction of target gene promoters (y-axis) at different distance cutoffs to the nearest LXRA and LXRB binding event (x-axis). Plots for the promoters of 48-hour GW3965+T0901317 repressed genes ($p<0.01$ and a fold change cutoff of -2 for both GW3965 and T0901317 treatments) are shown. ChIP-seq and RNA-seq data at 48 hours were used. LXRA and LXRB binding events coincident with JunD are in green (LXR+JunD), while LXR sites devoid of JunD occupancy are denoted in pink (LXR-JunD). The background distribution using all gene promoters in

the genome for sites overlapping with JunD (+JunD Bkgd; black) or for sites devoid of JunD (-JunD Bkgd; gray) is also graphed.

Figure S26. H3K27ac read enrichment at nuclear receptor binding sites

Histogram depicts the read depth ratios (x-axis) of H3K27ac enrichment at LXRA, LXRB and PPARG binding sites at 24 hours (top panel) and 48 hours (bottom panel). Negative values highlight stronger enrichment under control culture conditions (DMSO, 0 hours) while positive values denote stronger enrichment after drug treatment (24 or 48 hours of drug treatment).

Figure S27. Correlation of gene expression with RNAP2 promoter ChIP-seq enrichment

(A) Plot depicts correlation of gene expression (x-axis, RNA-seq RPKM) with RNAP2 promoter enrichment (y-axis, ChIP-seq reads +/-500-bp from TSS) after 24 hours of GW3965 (top panel, blue) and rosiglitazone (bottom panel, gold) treatments. Rank correlations are given in the top left of each plot. **(B)** Plot depicts correlation of gene expression (x-axis, RNA-seq RPKM) with RNAP2 promoter enrichment (y-axis, ChIP-seq reads +/-500-bp from TSS) after 48 hours of GW3965 (top panel, blue) and rosiglitazone (bottom panel, gold) treatments. Rank correlations are given in the top left of each plot.

Figure S28. Correlation of differentially expressed genes with RNAP2 promoter ChIP-seq enrichment at 24 hours of drug treatment (fold change +/-2)

(A) Smooth scatter plot comparing RNA-derived gene expression levels after 24 hours of GW3965 treatment (y-axis, RPKM) with read depth ratios of ChIP-derived RNAP2 promoter occupancy (x-axis). Read depth at promoters assesses the enrichment after 24 hours of GW3965 treatment (24h) normalized to control culture conditions (0h, DMSO). Data is presented for all GW3965 responsive genes (adjusted $p<0.01$, fold change cutoff of +/-2). **(B)** Smooth scatter plot comparing RNA-derived gene expression levels after 24 hours of rosiglitazone stimulation (y-axis, RPKM) with read depth ratios of ChIP-derived RNAP2 promoter occupancy (x-axis). Read depth at promoters assesses the enrichment after 24 hours of rosiglitazone (24h) normalized to control culture conditions (0h, DMSO). Data is presented for all GW3965 responsive genes (adjusted $p<0.01$, fold change cutoff of +/-2). fc= fold change.

Figure S29. Correlation of differentially expressed genes with RNAP2 promoter ChIP-seq enrichment after 48 hours of GW3965 treatment (fold change +/-1.5 and +/1.75)

(A) Smooth scatter plot comparing RNA-derived gene expression levels after 48 hours of GW3965 stimulation (y-axis, RPKM) with read depth ratios of ChIP-derived RNAP2 promoter occupancy (x-axis). Read depth at promoters assesses the enrichment after 48 hours of GW3965 (48h) normalized to control culture conditions (0h, DMSO). Data is presented for all GW3965 responsive genes (adjusted p<0.01, fold change cutoff of +/-1.5). **(B)** Smooth scatter plot comparing RNA-derived gene expression levels after 48 hours of GW3965 stimulation (y-axis, RPKM) with read depth ratios of ChIP-derived RNAP2 promoter occupancy (x-axis). Read depth at promoters assesses the enrichment after 48 hours of GW3965 (48h) normalized to control culture conditions (0h, DMSO). Data is presented for all GW3965 responsive genes (adjusted p<0.01, fold change cutoff of +/-1.75).

Figure S30. Correlation of differentially expressed genes with RNAP2 promoter ChIP-seq enrichment after 48 hours of rosiglitazone treatment (fold change +/-1.5 and +/1.75)

(A) Smooth scatter plot comparing RNA-derived gene expression levels after 48 hours of rosiglitazone stimulation (y-axis, RPKM) with read depth ratios of ChIP-derived RNAP2 promoter occupancy (x-axis). Read depth at promoters assesses the enrichment after 48 hours of rosiglitazone (48h) normalized to control culture conditions (0h, DMSO). Data is presented for all GW3965 responsive genes (adjusted p<0.01, fold change cutoff of +/-1.5). **(B)** Smooth scatter plot comparing RNA-derived gene expression levels after 48 hours of rosiglitazone stimulation (y-axis, RPKM) with read depth ratios of ChIP-derived RNAP2 promoter occupancy (x-axis). Read depth at promoters assesses the enrichment after 48 hours of rosiglitazone (48h) normalized to control culture conditions (0h, DMSO). Data is presented for all GW3965 responsive genes (adjusted p<0.01, fold change cutoff of +/-1.75).

Figure S31. Correlation of RNA-seq and RNAP2 promoter ChIP-seq enrichment between drug responses

(A) Smooth scatter plot comparing RNA-derived gene expression levels after 48 hours of GW3965 stimulation (y-axis, RPKM) with read depth ratios of ChIP-derived RNAP2 promoter occupancy using rosiglitazone treatment data (x-axis). Read depth at promoters assesses the enrichment after 48 hours of rosiglitazone (ROSI, 48h) normalized to control culture conditions (0h, DMSO). Data is presented for all GW3965 responsive genes (adjusted p<0.01, fold change cutoff of +/-2). **(B)** Smooth scatter plot comparing RNA-derived gene expression levels after 48 hours of rosiglitazone stimulation (y-axis, RPKM) ith read depth ratios of ChIP-derived RNAP2 promoter occupancy using GW3965 treatment data (x-axis). Read depth at promoters assesses the enrichment after 48 hours of GW3965 (48h)

normalized to control culture conditions (0h, DMSO). Data is presented for all rosiglitazone responsive genes (adjusted p<0.01, fold change cutoff of +/-2). fc= fold change.

Figure S32. UCSC genome browser image of the *DCBLD2* gene locus.

The *DCBLD2* genome locus is shown from the UCSC genome browser. Genome coordinates are given above the image. Raw ChIP-seq read enrichments for LXRA and LXRB binding after 2 hours and 48 hours is shown. RNAP2 enrichment at baseline (ctrl, DMSO) and after 24 and 48 hours of GW3965 stimulation is displayed. LXR binding exhibits stronger enrichment at 48 hours. At 48 hours, the *DCBLD2* promoter region also displays stronger occupancy, despite this gene being significantly repressed (adjusted p<0.01, fold change < -2) after 48 hours of GW3965 treatment.

Figure S33. UCSC genome browser image of the *GADD45A* gene locus.

The *GADD45A* genome locus is shown from the UCSC genome browser. Genome coordinates are given above the image. Raw ChIP-seq read enrichments for LXRA and LXRB binding after 2 hours and 48 hours is shown. RNAP2 enrichment at baseline (ctrl, DMSO) and after 24 and 48 hours of GW3965 stimulation is displayed. LXR binding exhibits stronger enrichment at 48 hours. At 48 hours, the *GADD45A* promoter region also displays stronger occupancy, despite this gene being significantly repressed (adjusted p<0.01, fold change < -2) after 48 hours of GW3965 treatment.

Figure S34. UCSC genome browser image of the *PPP1R15A* gene locus.

The *PPP1R15A* genome locus is shown from the UCSC genome browser. Genome coordinates are given above the image. Raw ChIP-seq read enrichments for LXRA and LXRB binding after 2 hours and 48 hours is shown. RNAP2 enrichment at baseline (ctrl, DMSO) and after 24 and 48 hours of GW3965 stimulation is displayed. LXR binding exhibits stronger enrichment at 48 hours. At 48 hours, the *PPP1R15A* promoter region also displays stronger occupancy, despite this gene being significantly repressed (adjusted p<0.01, fold change < -2) after 48 hours of GW3965 treatment.

Figure S35. UCSC genome browser image of the *PROSER2* gene locus.

The *PROSER2* genome locus is shown from the UCSC genome browser. Genome coordinates are given above the image. Raw ChIP-seq read enrichment for LXRA and LXRB binding after 2 hours and 48 hours is shown. RNAP2 enrichment at baseline (ctrl, DMSO) and after 24 and 48 hours of GW3965 stimulation is displayed. LXR binding exhibits stronger enrichment at 48 hours. At 48 hours, the *PROSER2* promoter region also displays stronger occupancy, despite this gene being significantly repressed (adjusted p<0.01, fold change < -2) after 48 hours of GW3965 treatment.

Figure S36. Correlation of gene expression with H3K36me3 gene body ChIP-seq enrichment

(A) Plot depicts correlation of gene expression (y-axis, RPKM) with H3K36me3 gene body ChIP-seq enrichment (x-axis, average H3K36me3 read depth per base pair in gene body) during control culture conditions (0h, DMSO). Rank correlations are given in the bottom right of each plot. **(B)** Plot depicts correlation of gene expression (y-axis, RPKM) with H3K36me3 gene body ChIP-seq enrichment (x-axis, average H3K36me3 read depth per base pair in gene body) after 48 hours of GW3965 treatment (48h). Rank correlations are given in the bottom right of each plot.

Figure S37. Correlation of 48-hour GW3965 responsive genes with H3K36me3 gene body ChIP-seq enrichment (fold change cutoff of +/-2)

Smooth scatter plot comparing RNA-derived gene expression levels after 48 hours of GW3965 stimulation (y-axis, RPKM) with read depth ratios of ChIP-derived H3K36me3 gene body occupancy (x-axis). Read depth at gene bodies assesses the enrichment after 48 hours of GW3965 (48h) normalized to control culture conditions (0h, DMSO). Data is presented for all GW3965 responsive genes (adjusted $p<0.01$, fold change cutoff of +/-2).

Figure S38. Correlation of 48-hour GW3965 responsive genes with H3K36me3 gene body ChIP-seq enrichment (fold change cutoff of +/-1.75)

(A) Smooth scatter plot comparing RNA-derived gene expression levels after 48 hours of GW3965 stimulation (y-axis, RPKM) with read depth ratios of ChIP-derived H3K36me3 gene body occupancy (x-axis). Read depth at gene bodies assesses the enrichment after 48 hours of GW3965 (48h) normalized to control culture conditions (0h, DMSO). Data is presented for all GW3965 responsive genes (adjusted $p<0.01$, fold change cutoff of +/-1.75). **(B)** Histogram tabulating the read depth ratios (x-axis) of H3K36me3 enrichment in gene bodies for genes repressed after 48 hours of GW3965 treatment. Negative values highlight stronger enrichment during control culture conditions (0h, DMSO) while positive values denote stronger enrichment after 48 hours of GW3965 treatment. Data is presented for all GW3965 responsive genes (adjusted $p<0.01$, fold change cutoff of +/-1.75).

Figure S39. Correlation of 48-hour GW3965 responsive genes with H3K36me3 gene body ChIP-seq enrichment (fold change cutoff of +/-1.5)

(A) Smooth scatter plot comparing RNA-derived gene expression levels after 48 hours of GW3965 stimulation (y-axis, RPKM) with read depth ratios of ChIP-derived H3K36me3 gene body occupancy (x-

axis). Read depth at gene bodies assesses the enrichment after 48 hours of GW3965 (48h) normalized to control culture conditions (0h, DMSO). Data is presented for all GW3965 responsive genes (adjusted p<0.01, fold change cutoff of +/-1.5). **(B)** Histogram tabulating the read depth ratios (x-axis) of H3K36me3 enrichment in gene bodies for genes repressed after 48 hours of GW3965 treatment. Negative values highlight stronger enrichment during control culture conditions (0h, DMSO) while positive values denote stronger enrichment after 48 hours of GW3965 treatment. Data is presented for all GW3965 responsive genes (adjusted p<0.01, fold change cutoff of +/-1.5).

Figure S40. Analysis of RNAP2 enrichment with H3K36me3 gene body ChIP-seq enrichment at 48 hours (fold change cutoff of 1.5)

(A) Violin plot of changes in RNAP2 read depth ratio in gene bodies of down-regulated genes after 48 hours of GW3965 treatment. Plots are displayed for genes that harbor LXRA binding events within 10kb (<10kb) from promoters, as well as plots for genes with no evidence of nearby LXRA occupancy (>100kb). **(B)** Violin plot of changes in RNAP2 read depth ratio in gene bodies of down-regulated genes after 48 hours of GW3965 treatment. Plots are displayed for genes that harbor LXRB binding events within 10kb (<10kb) from promoters, as well as plots for genes with no evidence of nearby LXRB occupancy (>100kb).

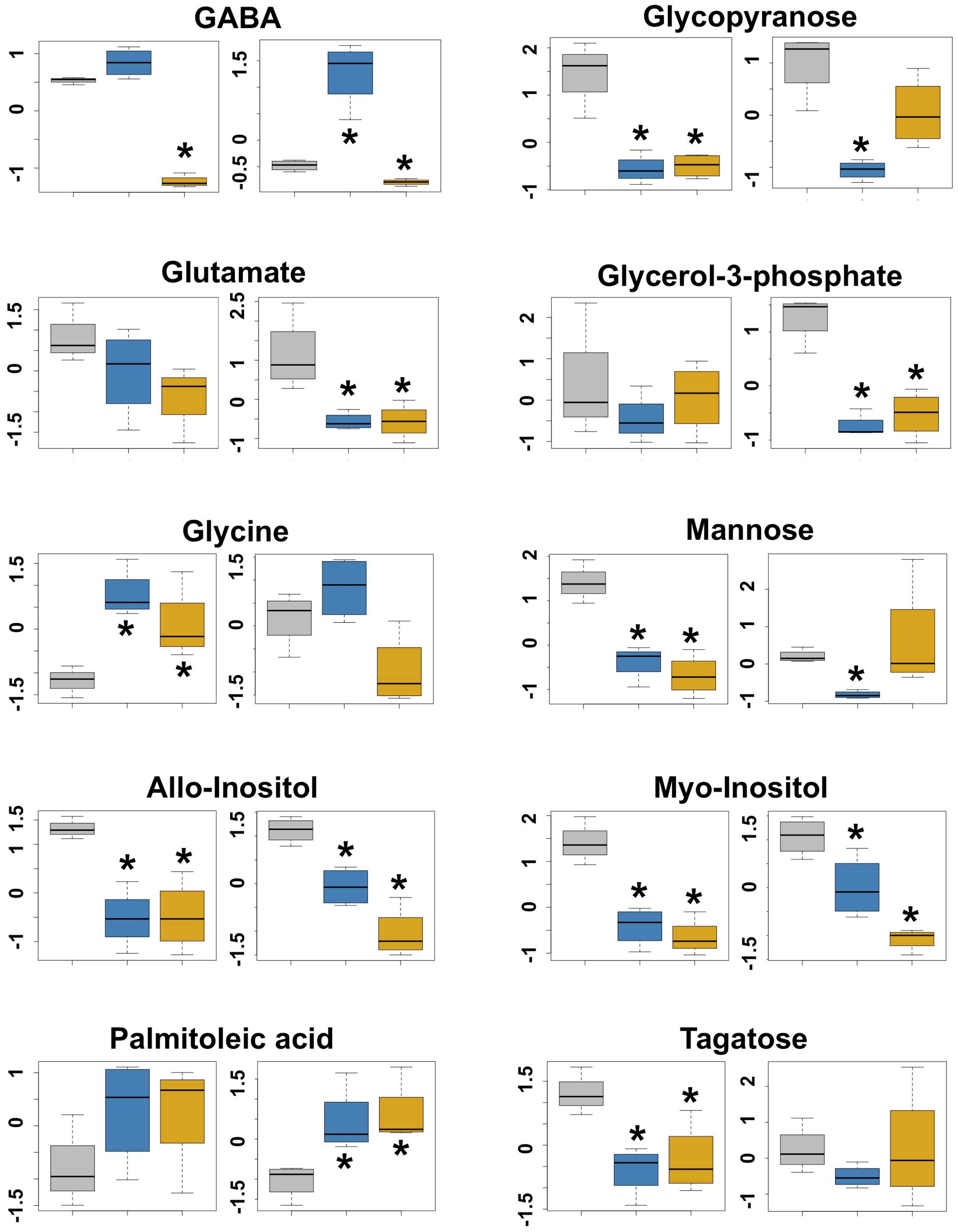
GABA
Glycopyranose
Glutamate
Glycerol-3-phosphate
Glycine
Mannose
Allo-Inositol
Myo-Inositol
Palmitoleic acid
Tagatose

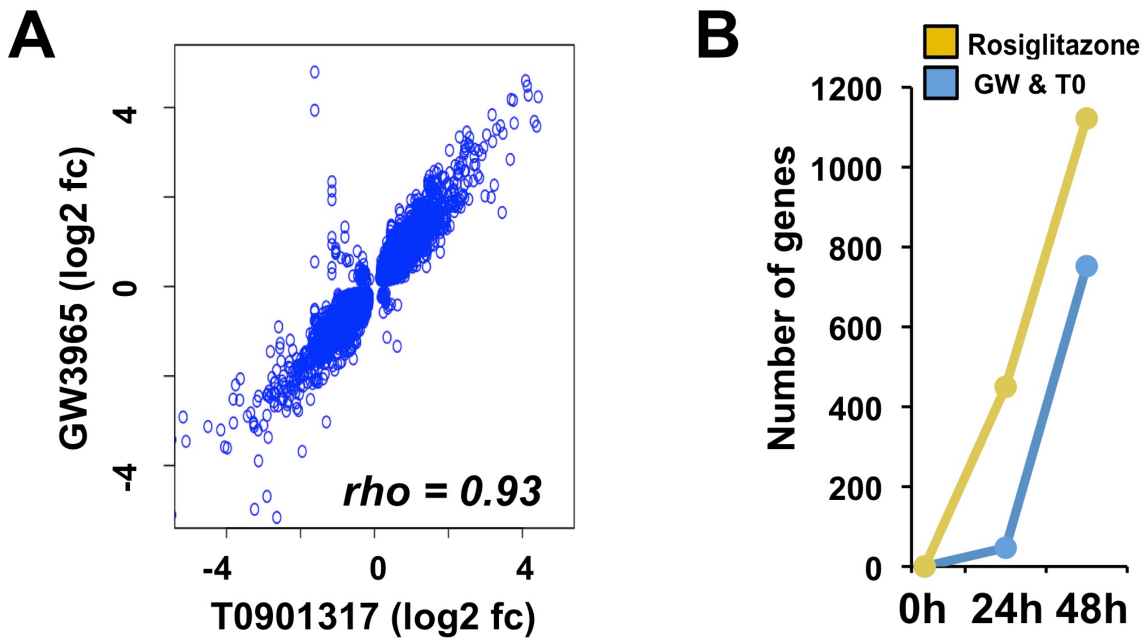
A
GW3965 (log2 fc)
4
0
-4
rho = 0.93
-4
0
4
T0901317 (log2 fc)
B
Rosiglitazone
GW & T0
Number of genes
1200
1000
800
600
400
200
0
0h
24h
48h

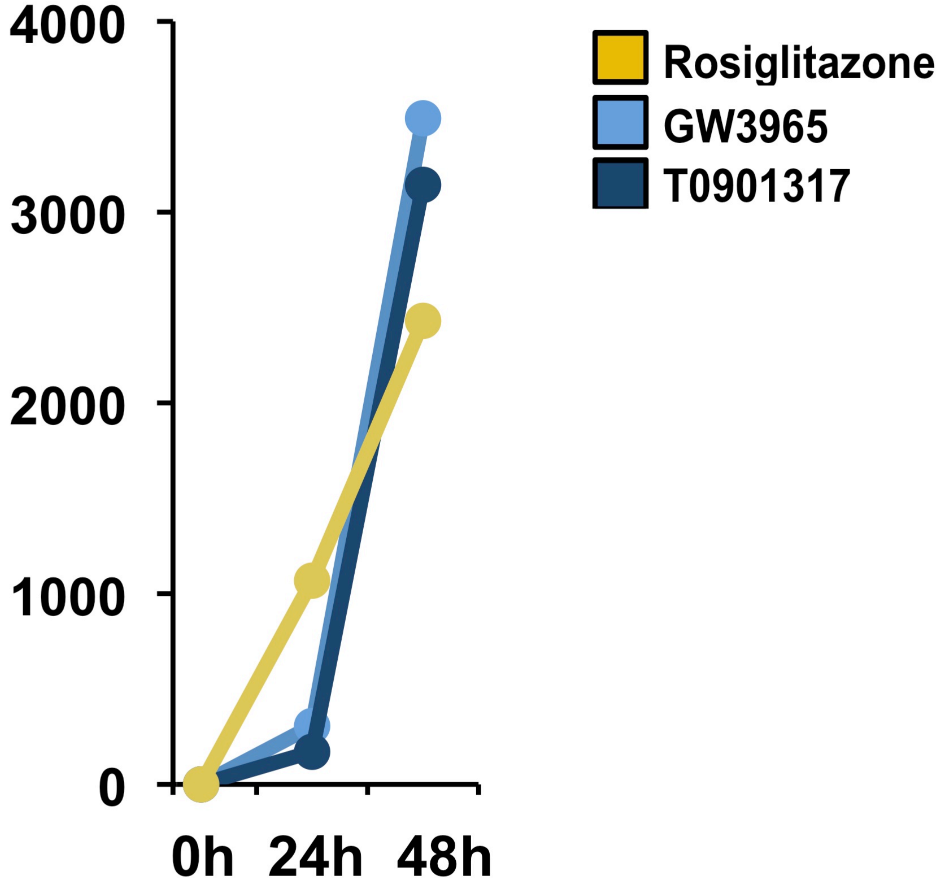
4000
3000
2000
1000
0
0h
24h
48h
Rosiglitazone
GW3965
T0901317

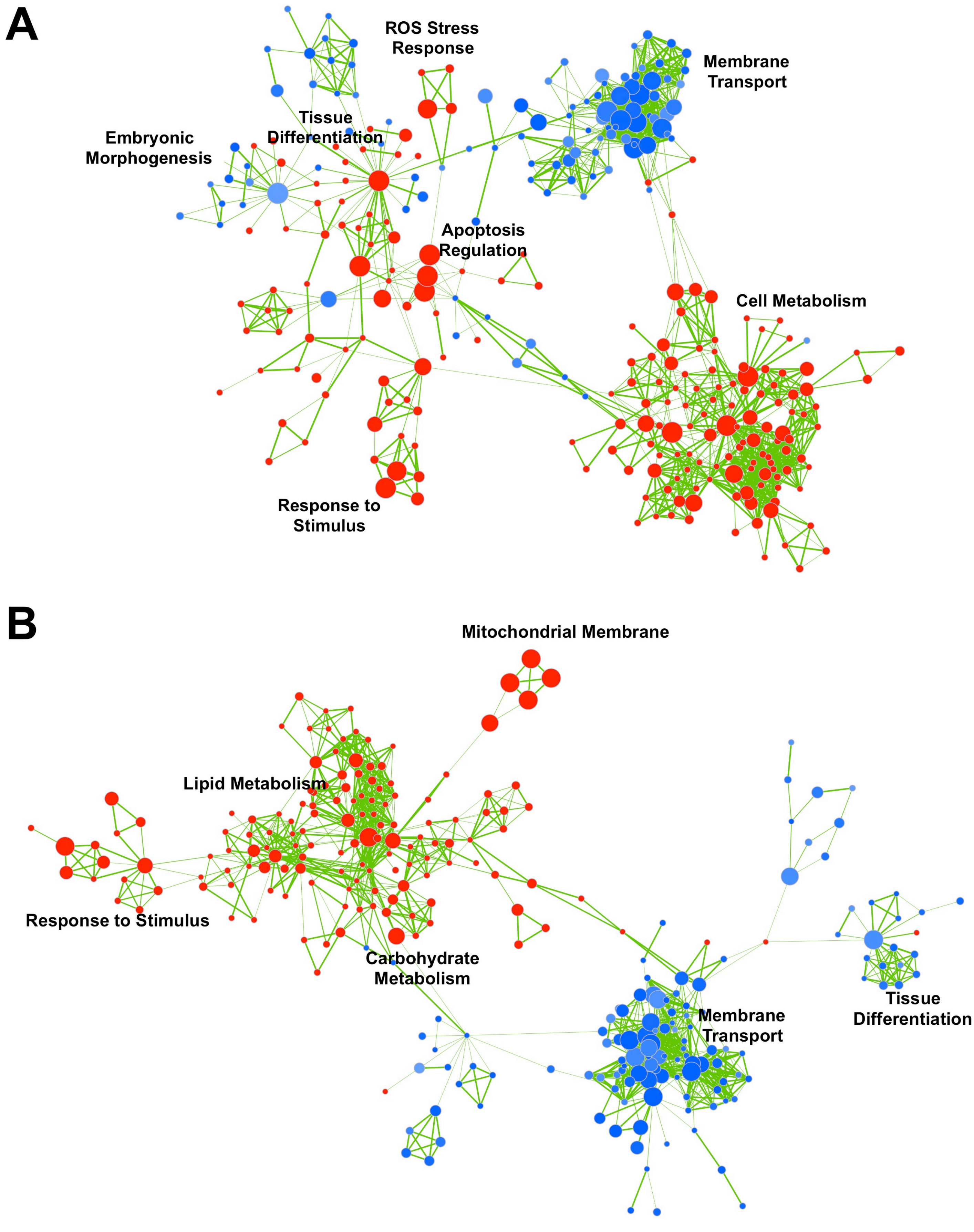
A
ROS Stress Response
Membrane Transport
Embryonic Morphogenesis
Tissue Differentiation
Apoptosis Regulation
Cell Metabolism
Response to Stimulus
B
Mitochondrial Membrane
Lipid Metabolism
Response to Stimulus
Carbohydrate Metabolism
Membrane Transport
Tissue Differentiation

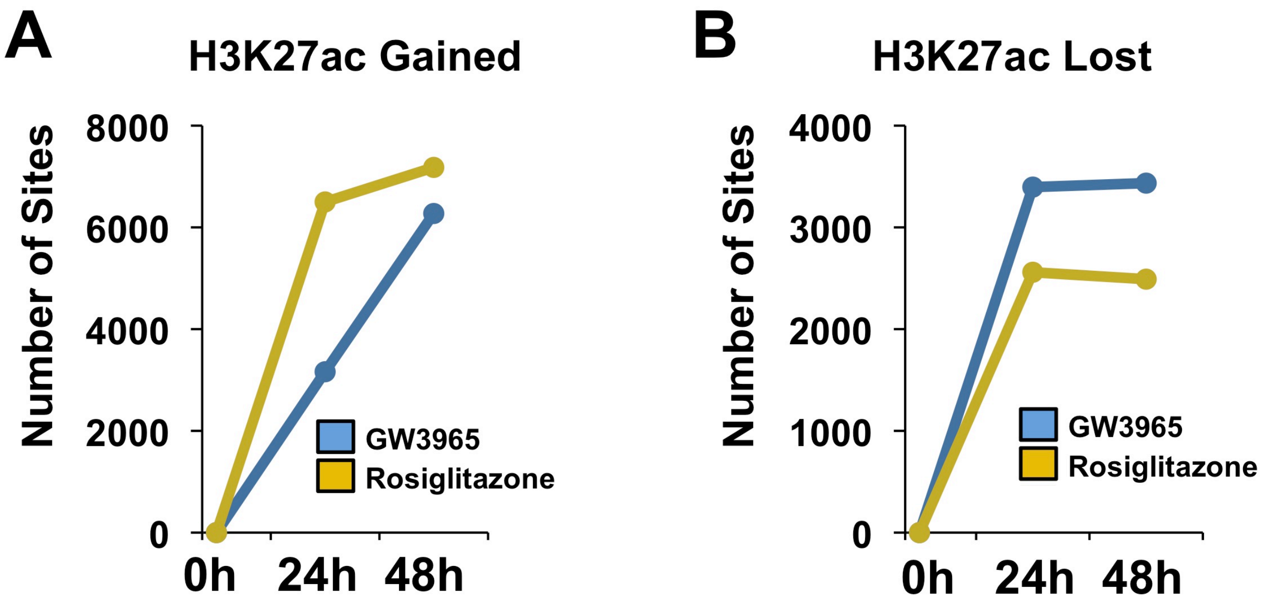
A
H3K27ac Gained
Number of Sites
8000
6000
4000
2000
0
0h
24h
48h
GW3965
Rosiglitazone
B
H3K27ac Lost
Number of Sites
4000
3000
2000
1000
0
0h
24h
48h
GW3965
Rosiglitazone

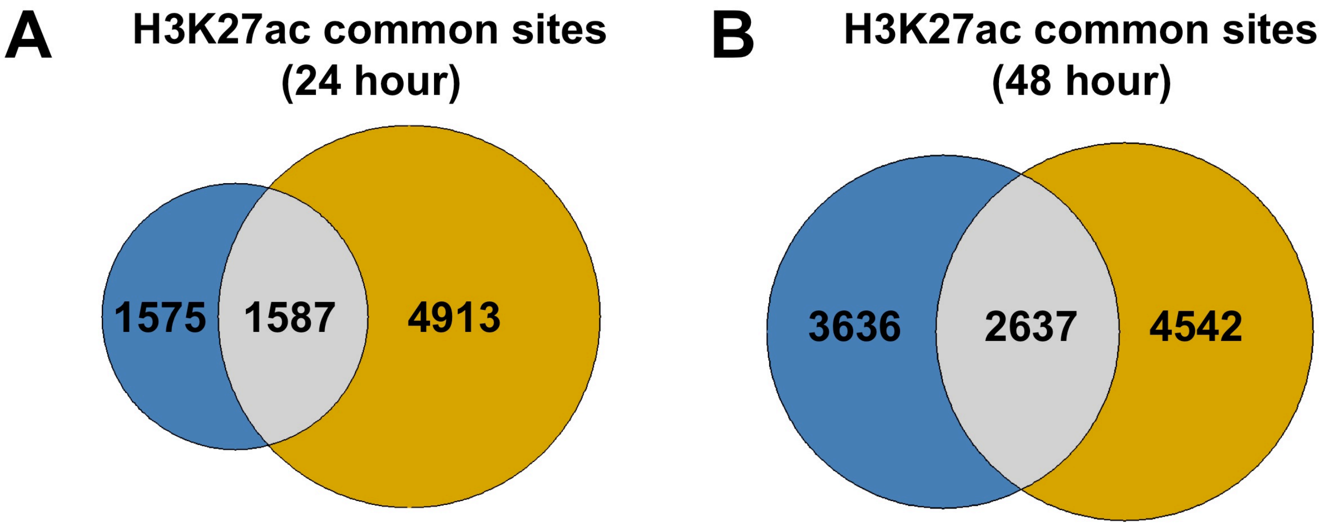

C

Database	Pathway Name	Signifcance -log10(Binomial p value)
GO Biological Process	Regulation of homotypic cell-cell adhesion	6.21
GO Biological Process	Inositol phosphate meatbolic process	4.26
GO Biological Process	Toll-like receptor 5 signaling pathway	3.72
GO Biological Process	Toll-like receptor 10 signaling pathway	3.72
GO Biological Process	Toll-like receptor 2 signaling pathway	3.4
GO Biological Process	Positive regulation of cation channel activity	3.26
GO Biological Process	Regulation of potassium ion transmembrane transporter activity	3.13
MSigDB Pathway	Stablilization and expansion of the E-cadherin adherens junction	3.54
MSigDB Pathway	Mechanism of Gene Regulation by Peroxisome Proliferators via PPARa	3

D

Database	Pathway Name	Signifcance -log10(Binomial p value)
GO Biological Process	Response to osmotic stress	10.15
GO Biological Process	Regulation of mesenchymal cell proliferation	7.47
GO Biological Process	Cellular hormone metabolic process	6.53
GO Biological Process	Retinoid metabolic process	6.1
GO Biological Process	Glycoside metabolic process	5.44
GO Biological Process	Cellular monvalent inorganic cation homeostasis	4.84
GO Biological Process	Regulation of carbohydrate biosynthetic process	4.69
GO Biological Process	Regulation of intracellular pH	4.61
GO Biological Process	Inactivation of MAPK activity	3.93
GO Biological Process	Detection of calcium ion	3.06
MSigDB Pathway	ErbB1 downstream signaling	10.73
MSigDB Pathway	IL17 signaling pathway	10.14
MSigDB Pathway	Genes involved in PPARA activates gene expression	7.68
MSigDB Pathway	PDGFR-beta signaling pathway	7.38
MSigDB Pathway	Calcium signaling in the CD4+ TCR pathway	7.26
MSigDB Pathway	IL6-mediated signaling events	6.59
MSigDB Pathway	Genes involved in apoptotic cleavage of cell adhesion proteins	6.47
MSigDB Pathway	Stabilization and expansion of the E-cadherin adherens junction	5.75
MSigDB Pathway	TGF-beta receptor signaling	5.63
MSigDB Pathway	Genes involved in MAPK targets/ Nulear events mediated by MAP kinases	4.74
Panther Pathway	Apoptosis signaling pathway	7.73
Panther Pathway	Oxidative stress response	5.14

A

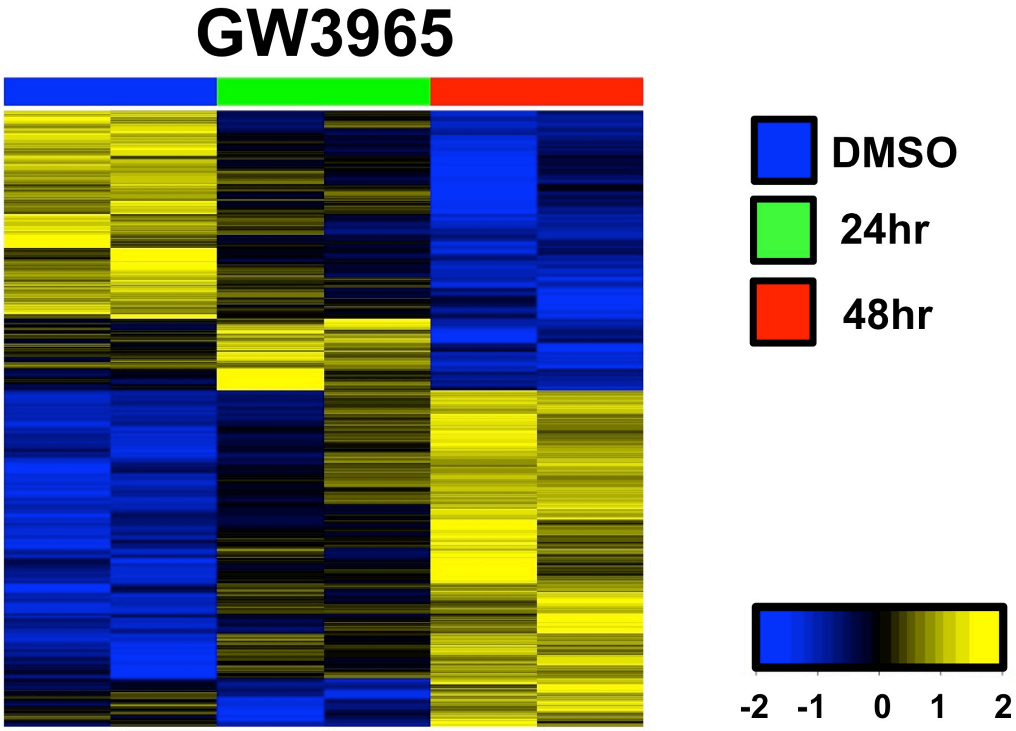
GW3965
DMSO
24hr
48hr
-2 -1 0 1 2

B

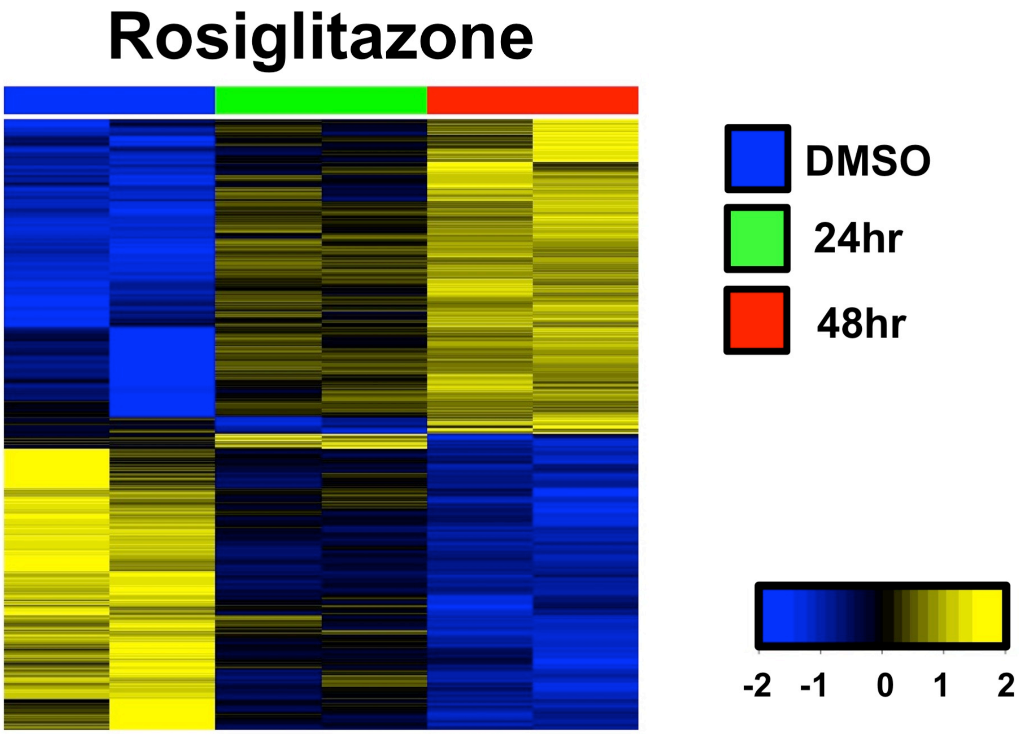
Rosiglitazone
DMSO
24hr
48hr
-2 -1 0 1 2

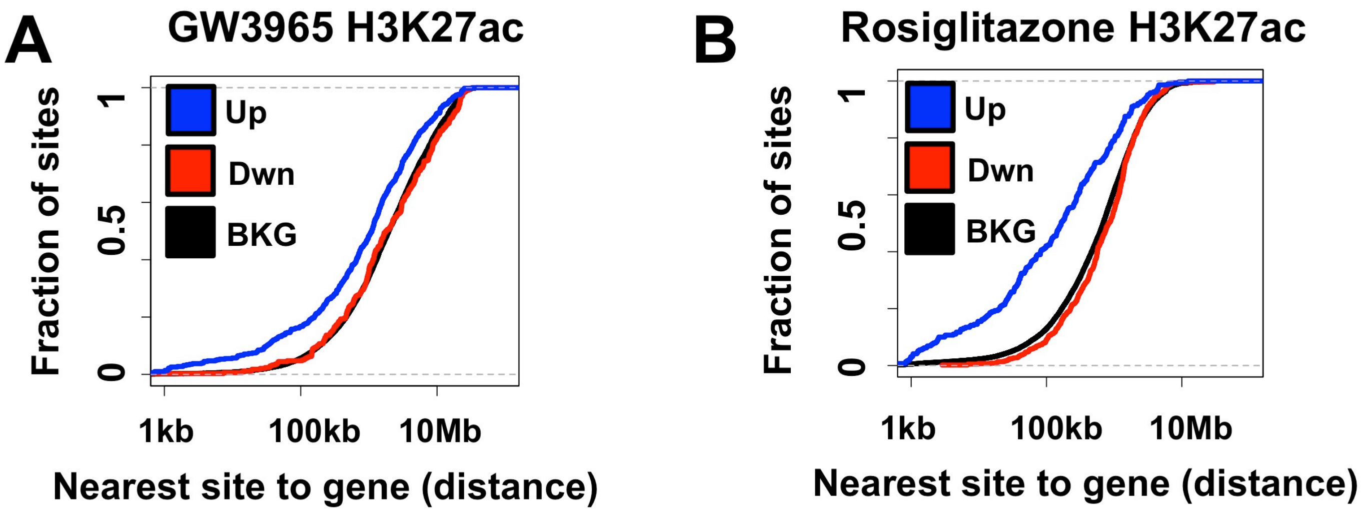
A
GW3965 H3K27ac
Fraction of sites
1
0.5
0
Up
Dwn
BKG
1kb
100kb
10Mb
Nearest site to gene (distance)
B
Rosiglitazone H3K27ac
Fraction of sites
1
0.5
0
Up
Dwn
BKG
1kb
100kb
10Mb
Nearest site to gene (distance)

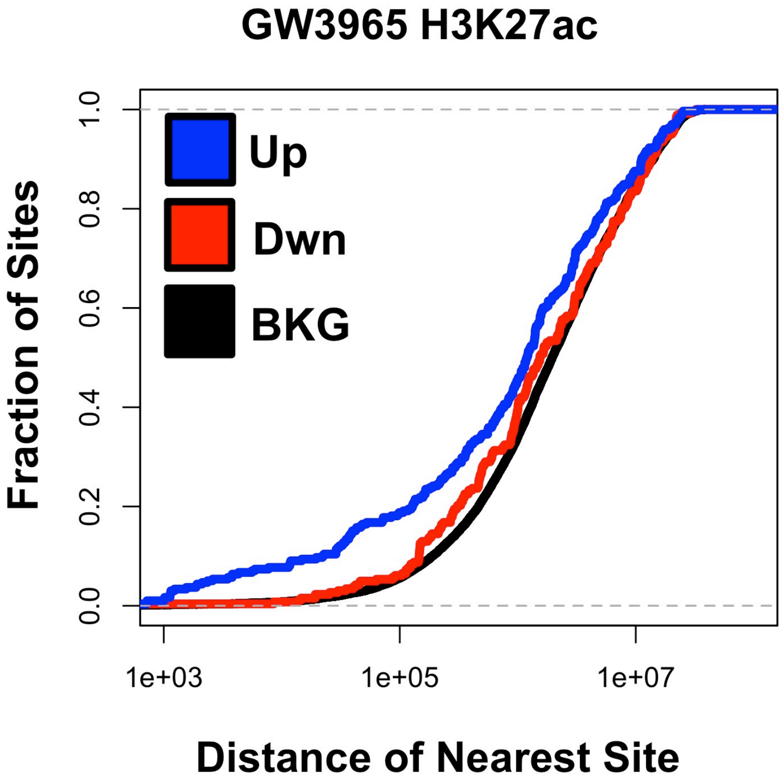

GW3965 H3K27ac
Up
Dwn
BKG
Fraction of Sites
Distance of Nearest Site
0.0
0.2
0.4
0.6
0.8
1.0
1e+03
1e+05
1e+07

A

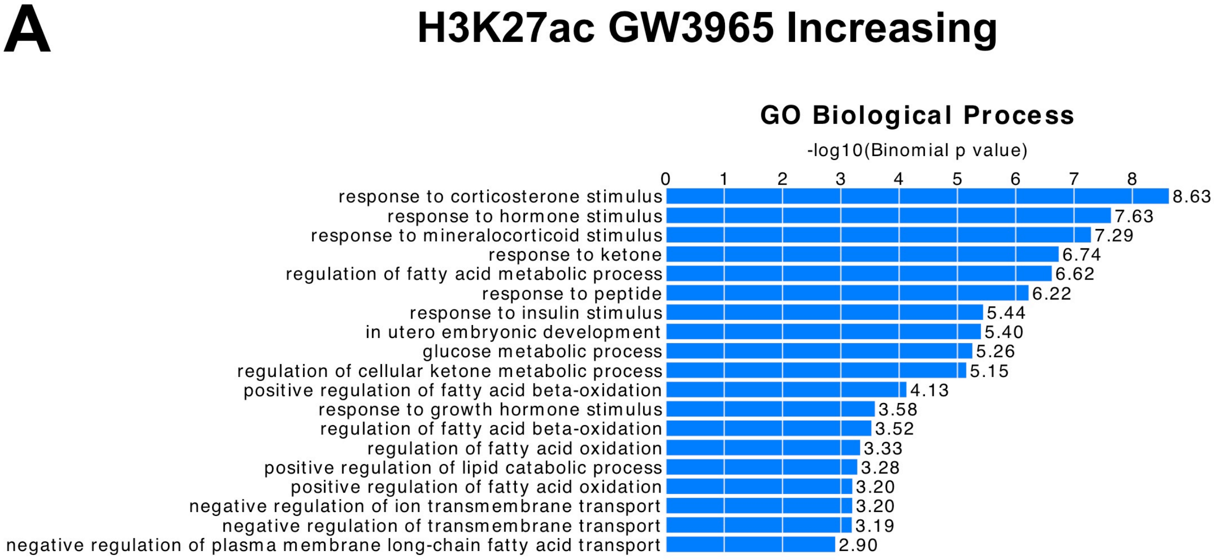
H3K27ac GW3965 Increasing
GO Biological Process
-log10(Binomial p value)
0 1 2 3 4 5 6 7 8
response to corticosterone stimulus 8.63
response to hormone stimulus 7.63
response to mineralocorticoid stimulus 7.29
response to ketone 6.74
regulation of fatty acid metabolic process 6.62
response to peptide 6.22
response to insulin stimulus 5.44
in utero embryonic development 5.40
glucose metabolic process 5.26
regulation of cellular ketone metabolic process 5.15
positive regulation of fatty acid beta-oxidation 4.13
response to growth hormone stimulus 3.58
regulation of fatty acid beta-oxidation 3.52
regulation of fatty acid oxidation 3.33
positive regulation of lipid catabolic process 3.28
positive regulation of fatty acid oxidation 3.20
negative regulation of ion transmembrane transport 3.20
negative regulation of transmembrane transport 3.19
negative regulation of plasma membrane long-chain fatty acid transport 2.90

B

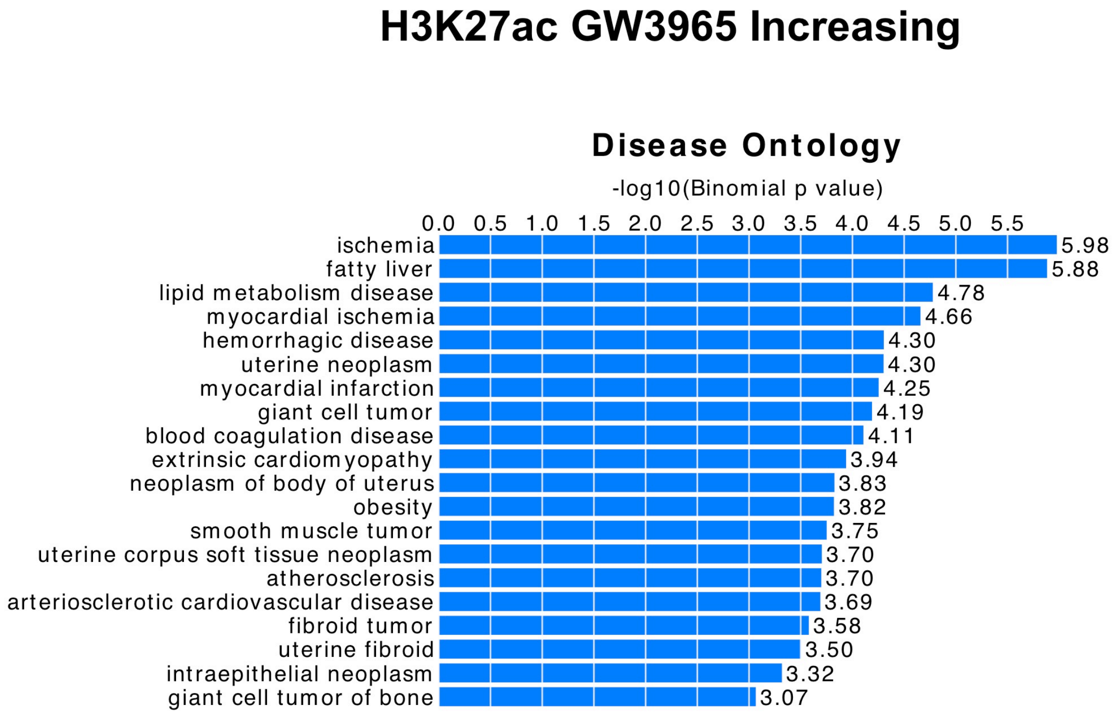
H3K27ac GW3965 Increasing
Disease Ontology
-log10(Binomial p value)
0.0 0.5 1.0 1.5 2.0 2.5 3.0 3.5 4.0 4.5 5.0 5.5
ischemia 5.98
fatty liver 5.88
lipid metabolism disease 4.78
myocardial ischemia 4.66
hemorrhagic disease 4.30
uterine neoplasm 4.30
myocardial infarction 4.25
giant cell tumor 4.19
blood coagulation disease 4.11
extrinsic cardiomyopathy 3.94
neoplasm of body of uterus 3.83
obesity 3.82
smooth muscle tumor 3.75
uterine corpus soft tissue neoplasm 3.70
atherosclerosis 3.70
arteriosclerotic cardiovascular disease 3.69
fibroid tumor 3.58
uterine fibroid 3.50
intraepithelial neoplasm 3.32
giant cell tumor of bone 3.07

A

H3K27ac Rosiglitazone Increasing

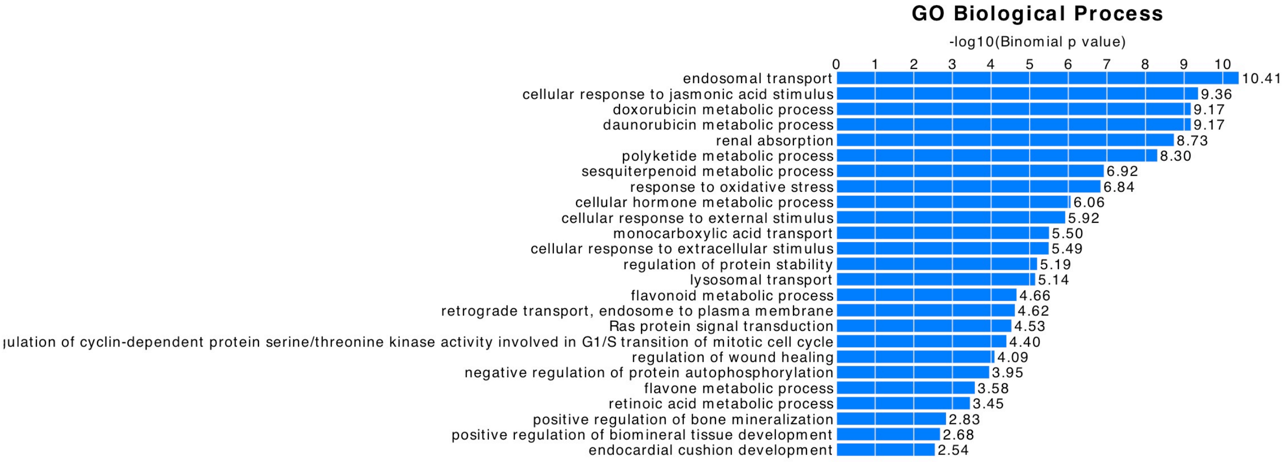

B

H3K27ac Rosiglitazone Increasing

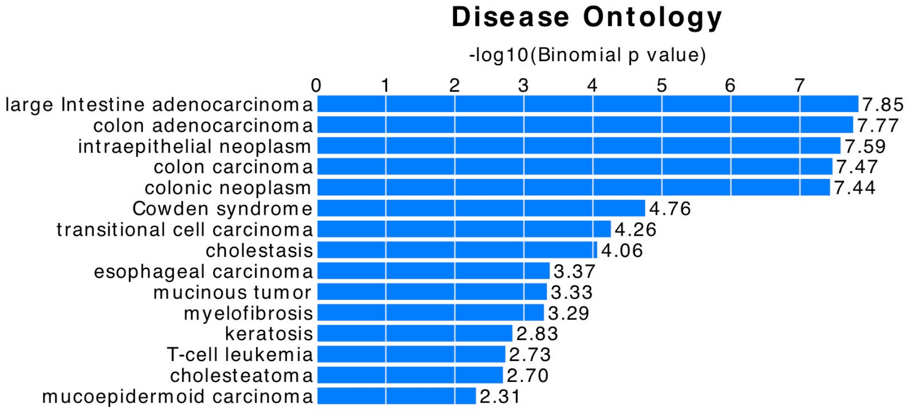

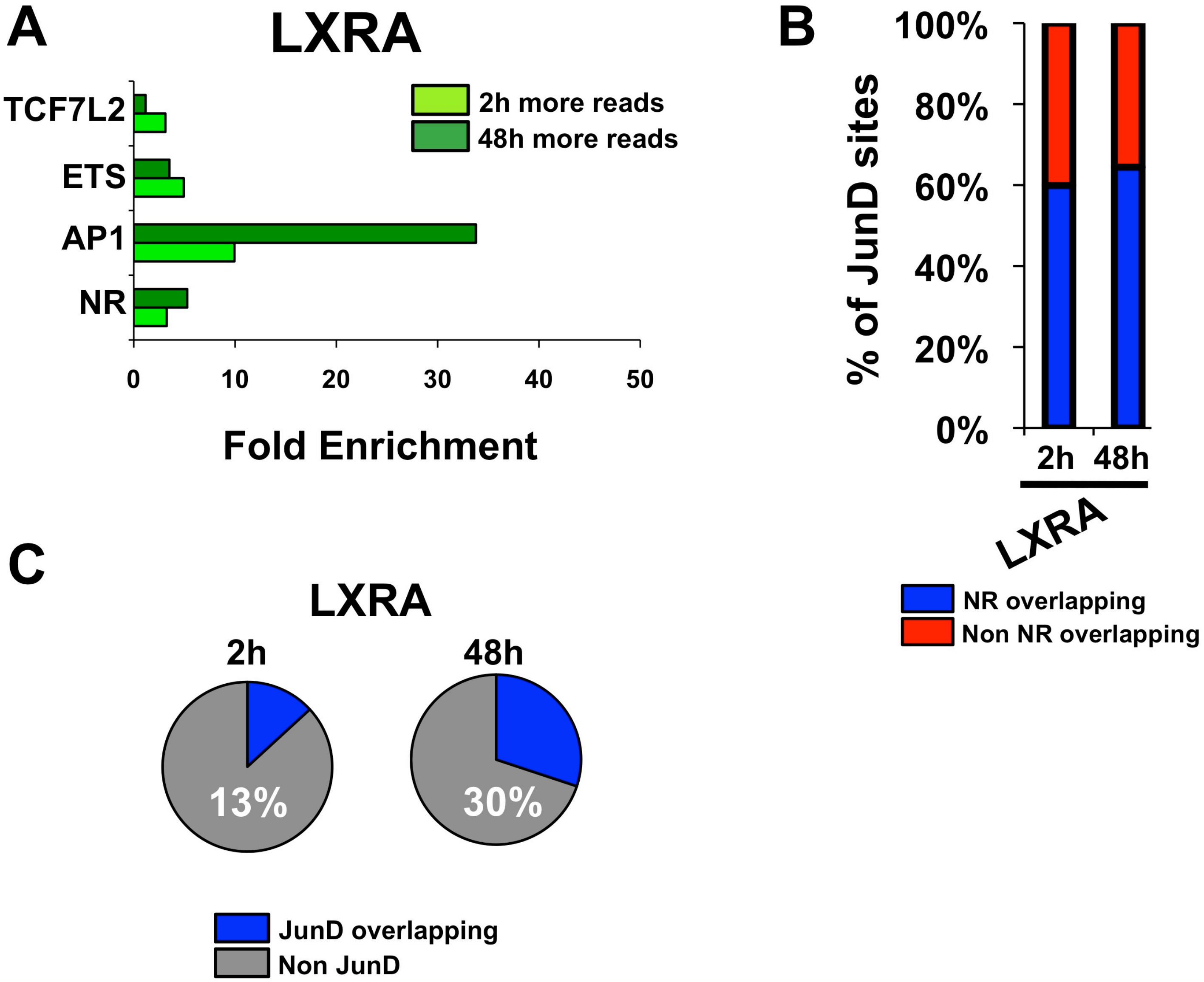
A
LXRA
TCF7L2
ETS
AP1
NR
2h more reads
48h more reads
0
10
20
30
40
50
Fold Enrichment
B
100%
80%
60%
40%
20%
0%
% of JunD sites
2h
48h
LXRA
NR overlapping
Non NR overlapping
C
LXRA
2h
48h
13%
30%
JunD overlapping
Non JunD

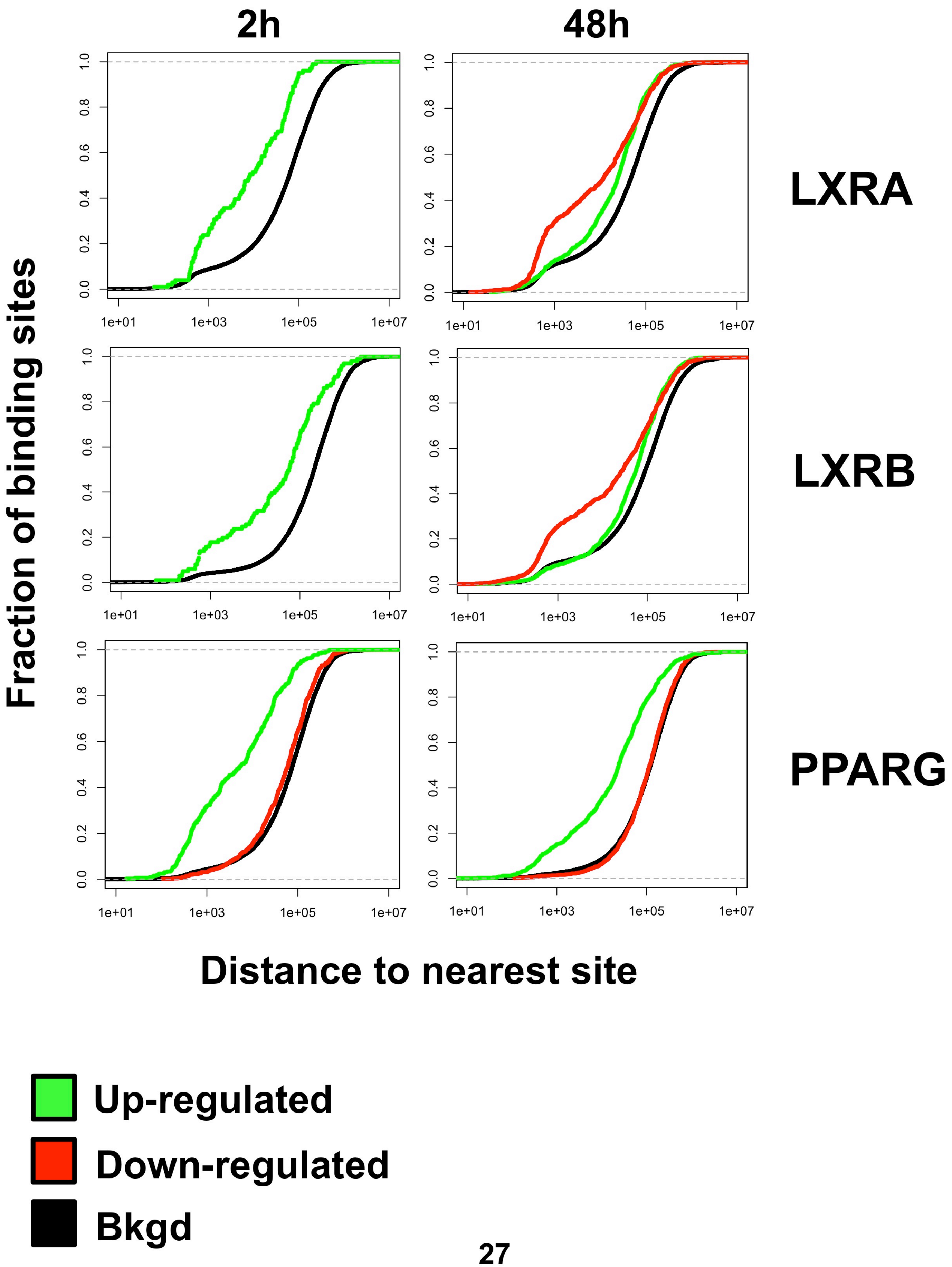
2h
48h
LXRA
LXRB
PPARG
Fraction of binding sites
Distance to nearest site
0.0
0.2
0.4
0.6
0.8
1.0
1e+01
1e+03
1e+05
1e+07
Up-regulated
Down-regulated
Bkgd

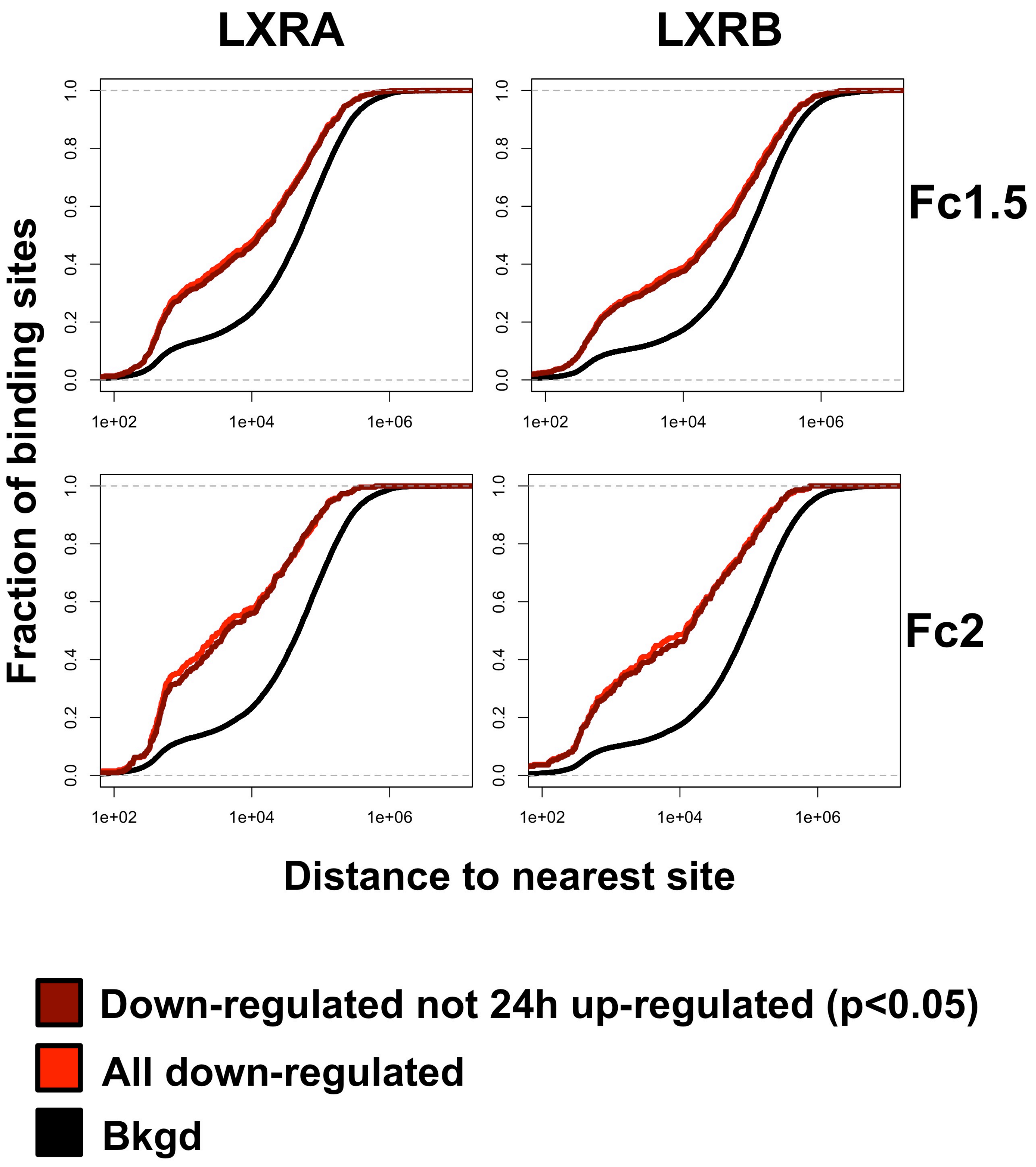
LXRA
LXRB
Fc1.5
Fc2
Fraction of binding sites
Distance to nearest site
0.0
0.2
0.4
0.6
0.8
1.0
1e+02
1e+04
1e+06
Down-regulated not 24h up-regulated (p<0.05)
All down-regulated
Bkgd

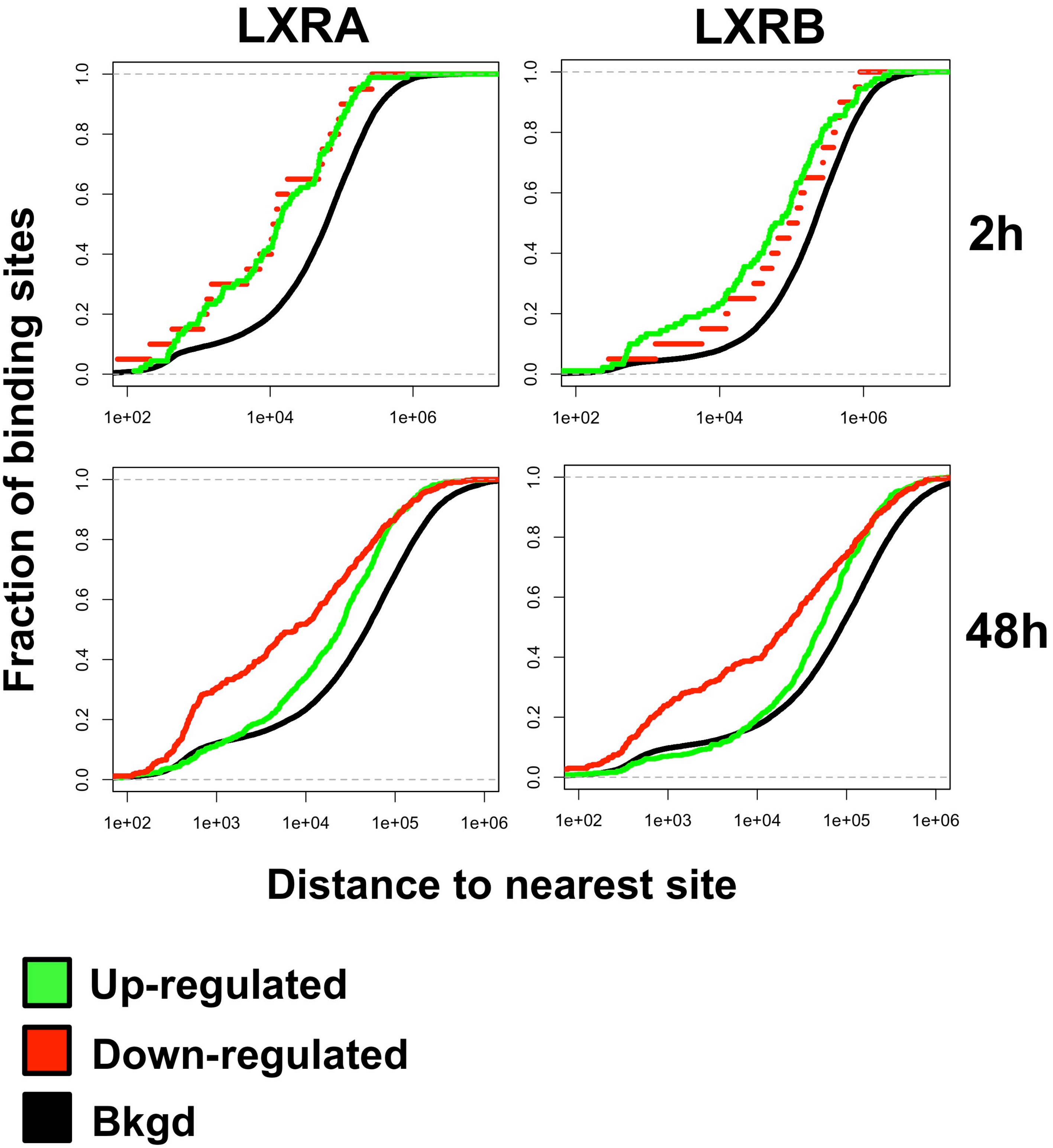
LXRA
LXRB
2h
48h
Fraction of binding sites
Distance to nearest site
0.0
0.2
0.4
0.6
0.8
1.0
1e+02
1e+03
1e+04
1e+05
1e+06
Up-regulated
Down-regulated
Bkgd

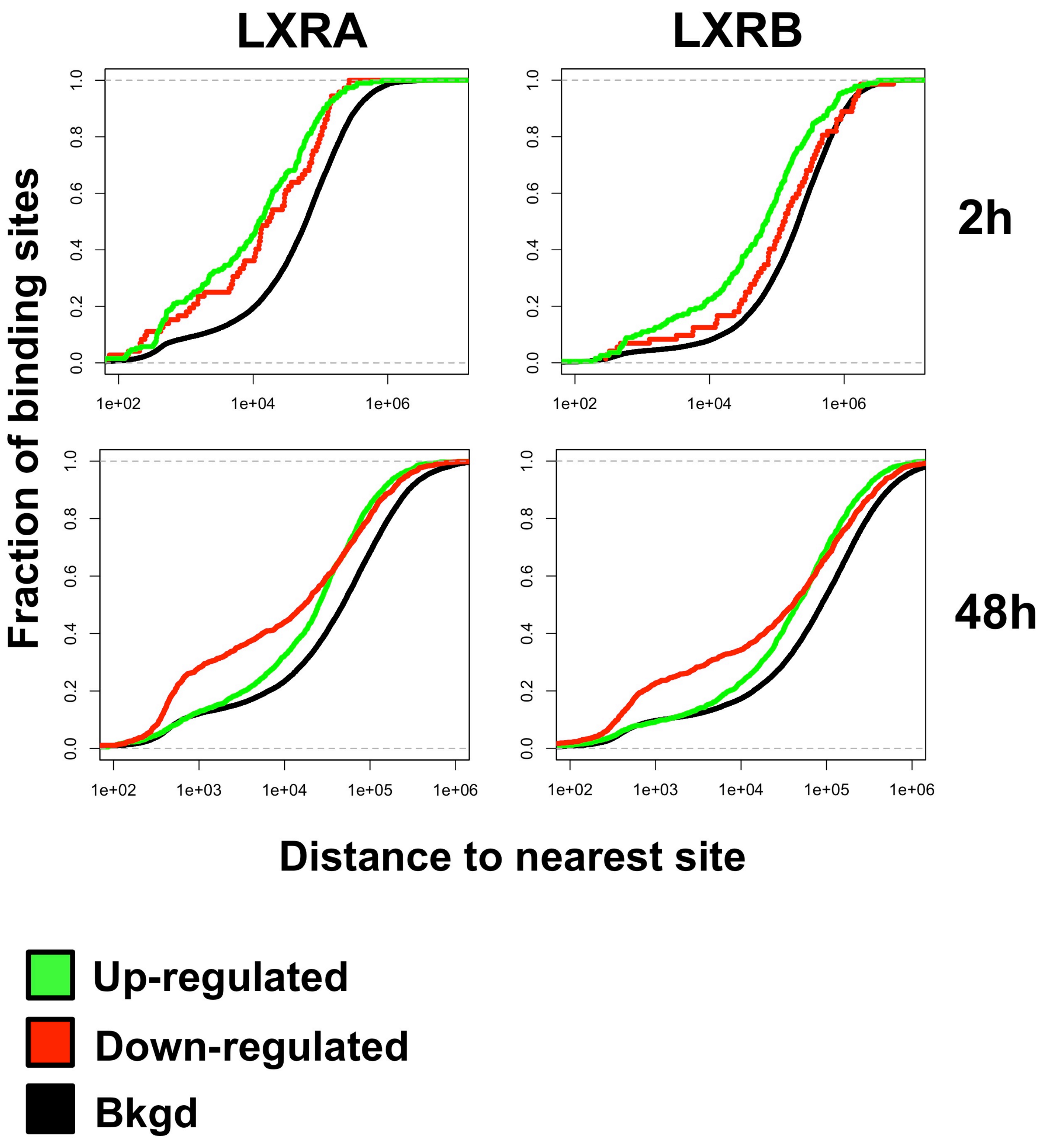
LXRA
LXRB
2h
48h
Fraction of binding sites
Distance to nearest site
0.0
0.2
0.4
0.6
0.8
1.0
1e+02
1e+03
1e+04
1e+05
1e+06
Up-regulated
Down-regulated
Bkgd

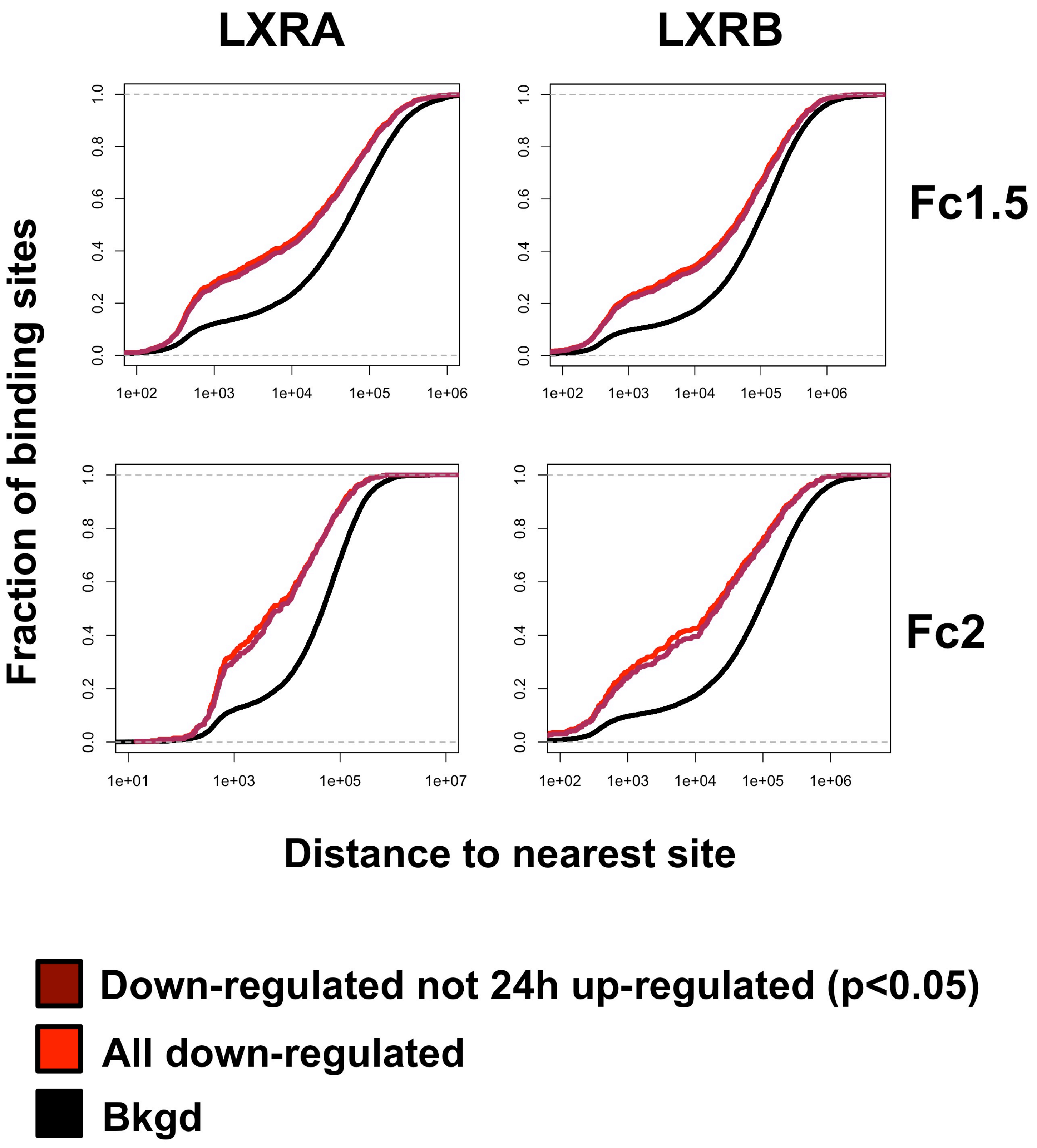
LXRA
LXRB
Fc1.5
Fc2
Fraction of binding sites
Distance to nearest site
0.0
0.2
0.4
0.6
0.8
1.0
1e+01
1e+02
1e+03
1e+04
1e+05
1e+06
1e+07
Down-regulated not 24h up-regulated (p<0.05)
All down-regulated
Bkgd

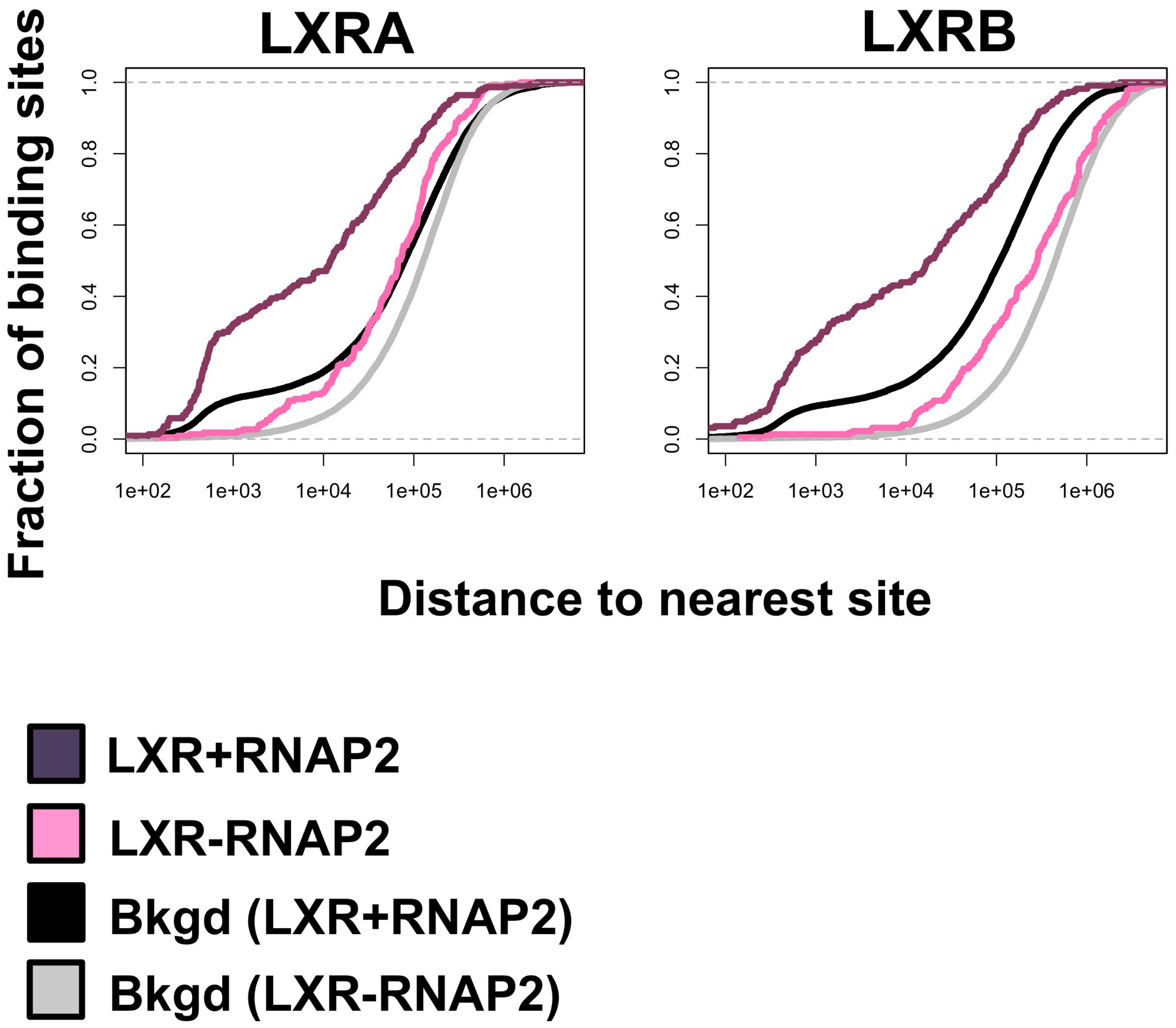
LXRA
LXRB
Fraction of binding sites
0.0
0.2
0.4
0.6
0.8
1.0
1e+02
1e+03
1e+04
1e+05
1e+06
Distance to nearest site
LXR+RNAP2
LXR-RNAP2
Bkgd (LXR+RNAP2)
Bkgd (LXR-RNAP2)

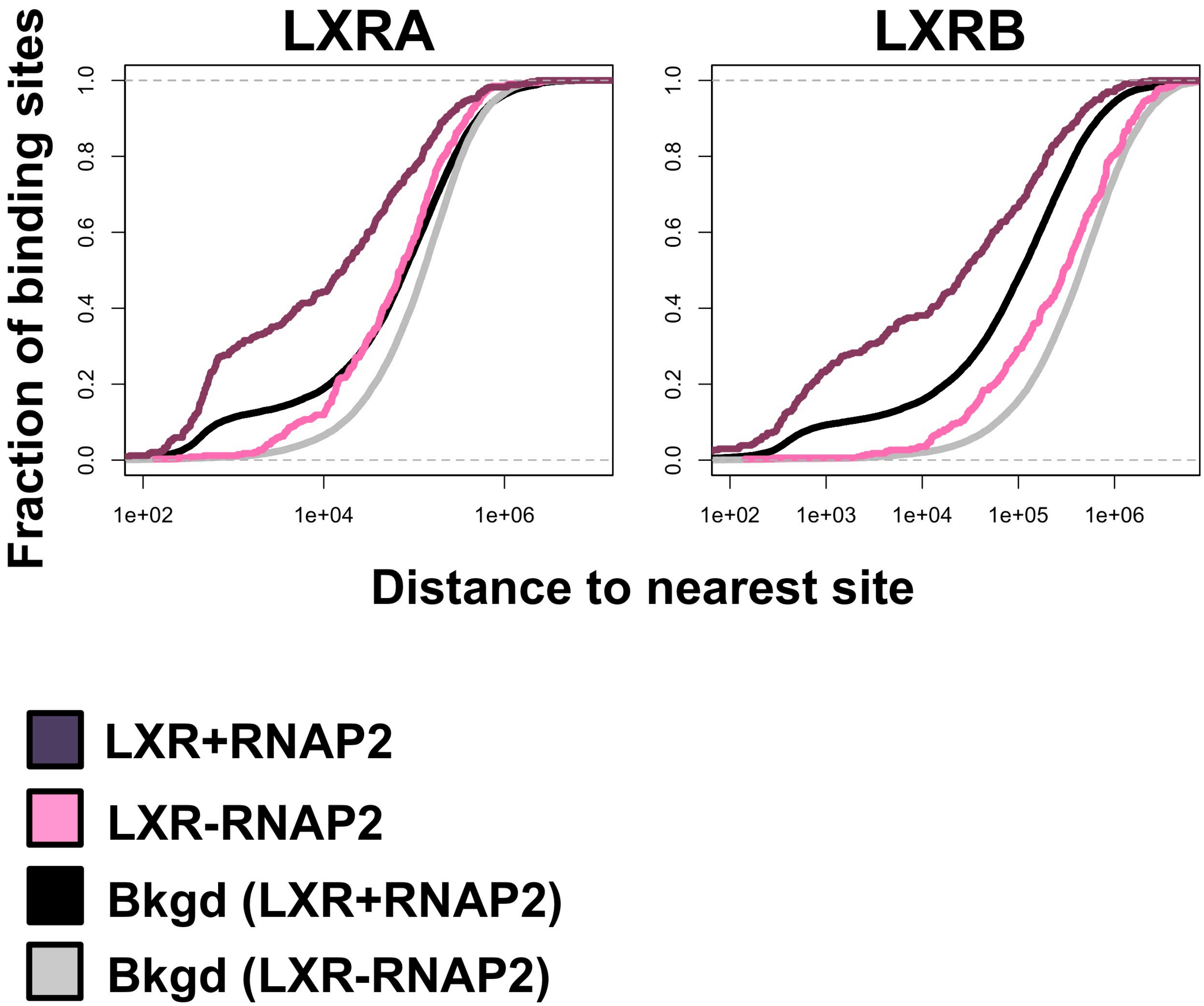
LXRA
LXRB
Fraction of binding sites
0.0
0.2
0.4
0.6
0.8
1.0
1e+02
1e+03
1e+04
1e+05
1e+06
Distance to nearest site
LXR+RNAP2
LXR-RNAP2
Bkgd (LXR+RNAP2)
Bkgd (LXR-RNAP2)

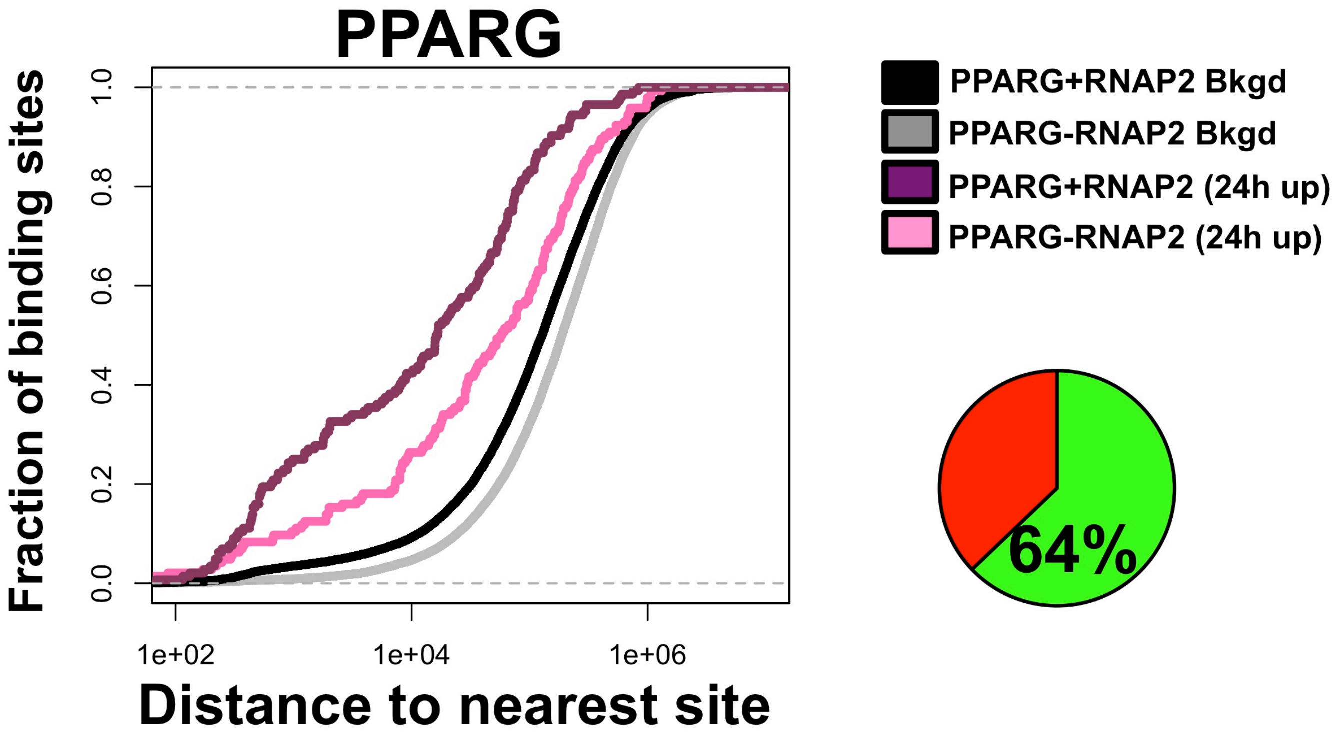
PPARG
Fraction of binding sites
0.0
0.2
0.4
0.6
0.8
1.0
1e+02
1e+04
1e+06
Distance to nearest site
PPARG+RNAP2 Bkgd
PPARG-RNAP2 Bkgd
PPARG+RNAP2 (24h up)
PPARG-RNAP2 (24h up)
64%

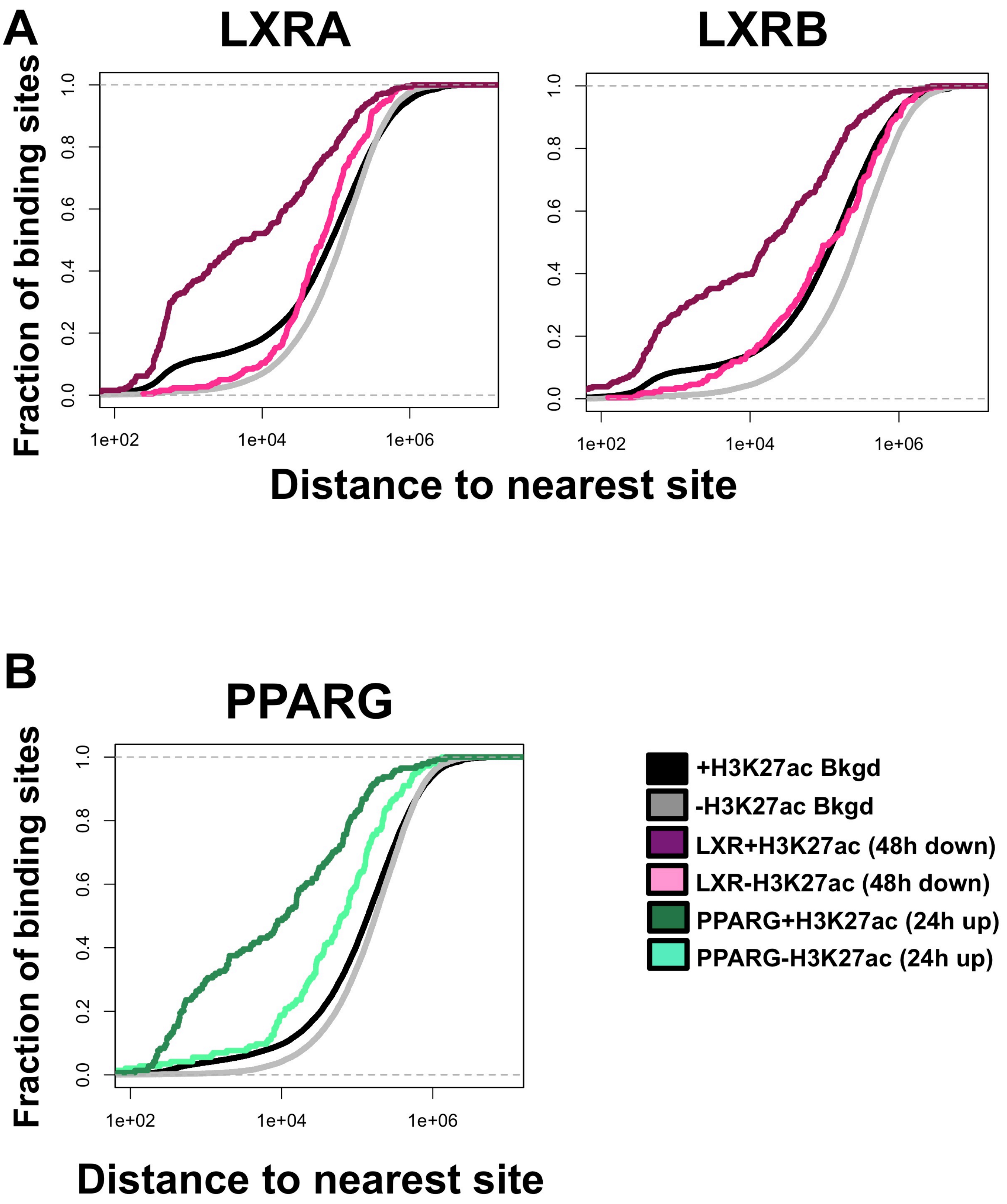
A
LXRA
LXRB
Fraction of binding sites
0.0
0.2
0.4
0.6
0.8
1.0
1e+02
1e+04
1e+06
Distance to nearest site
B
PPARG
Fraction of binding sites
0.0
0.2
0.4
0.6
0.8
1.0
1e+02
1e+04
1e+06
Distance to nearest site
+H3K27ac Bkgd
-H3K27ac Bkgd
LXR+H3K27ac (48h down)
LXR-H3K27ac (48h down)
PPARG+H3K27ac (24h up)
PPARG-H3K27ac (24h up)

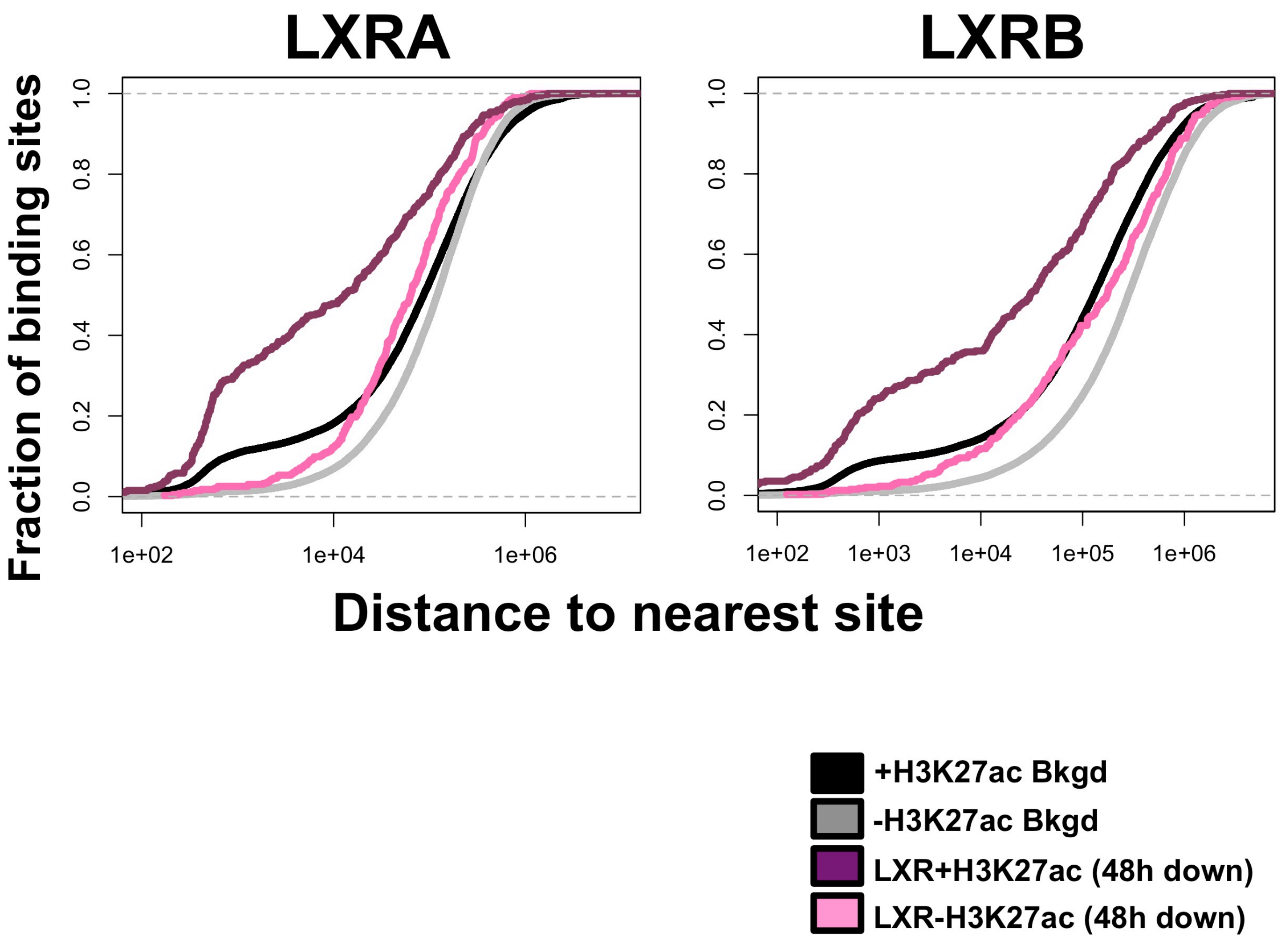

LXRA
LXRB
Fraction of binding sites
0.0
0.2
0.4
0.6
0.8
1.0
1e+02
1e+03
1e+04
1e+05
1e+06
Distance to nearest site
+H3K27ac Bkgd
-H3K27ac Bkgd
LXR+H3K27ac (48h down)
LXR-H3K27ac (48h down)

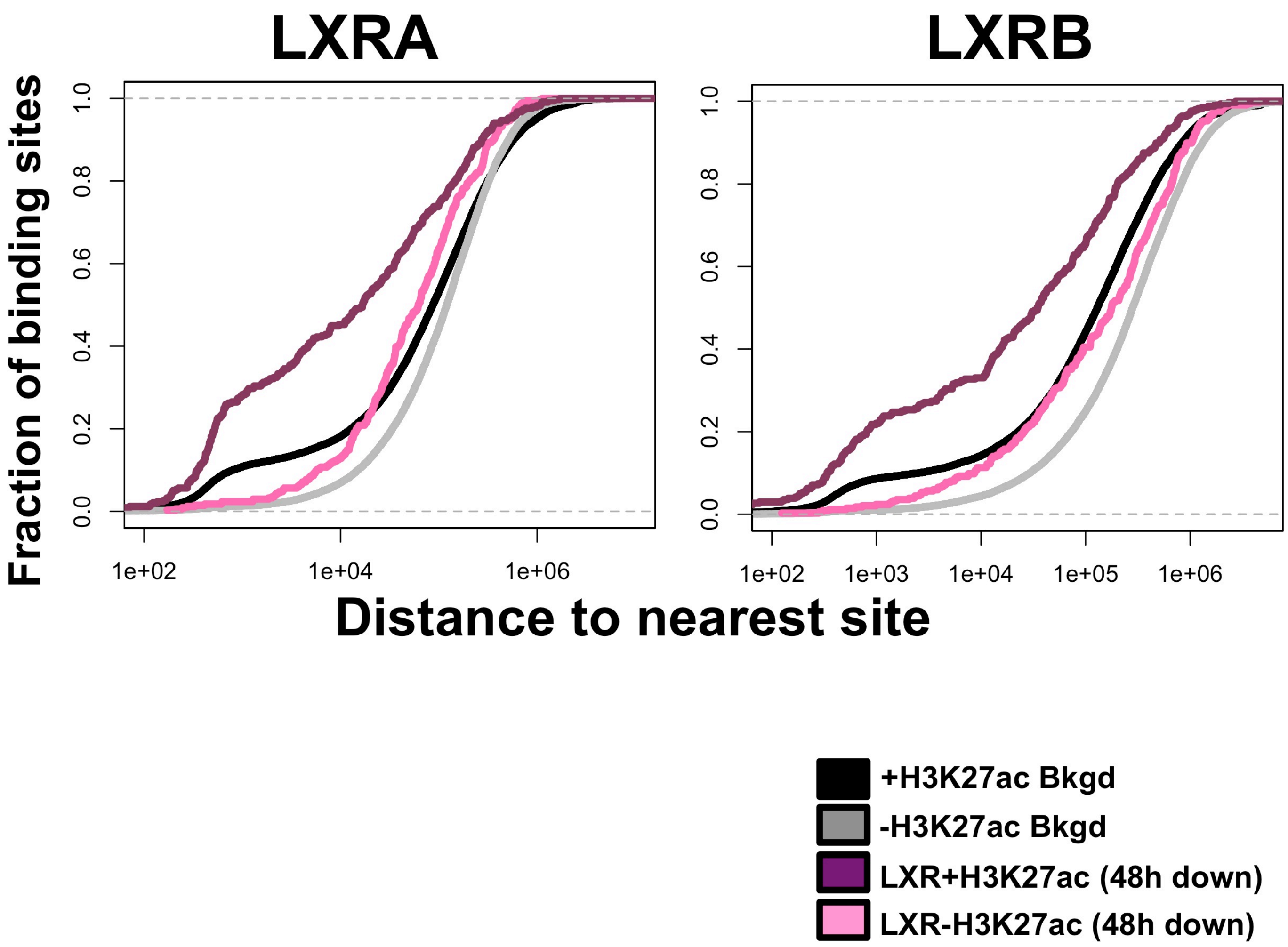

LXRA
LXRB
Fraction of binding sites
0.0
0.2
0.4
0.6
0.8
1.0
1e+02
1e+04
1e+06
1e+02
1e+03
1e+04
1e+05
1e+06
Distance to nearest site
+H3K27ac Bkgd
-H3K27ac Bkgd
LXR+H3K27ac (48h down)
LXR-H3K27ac (48h down)

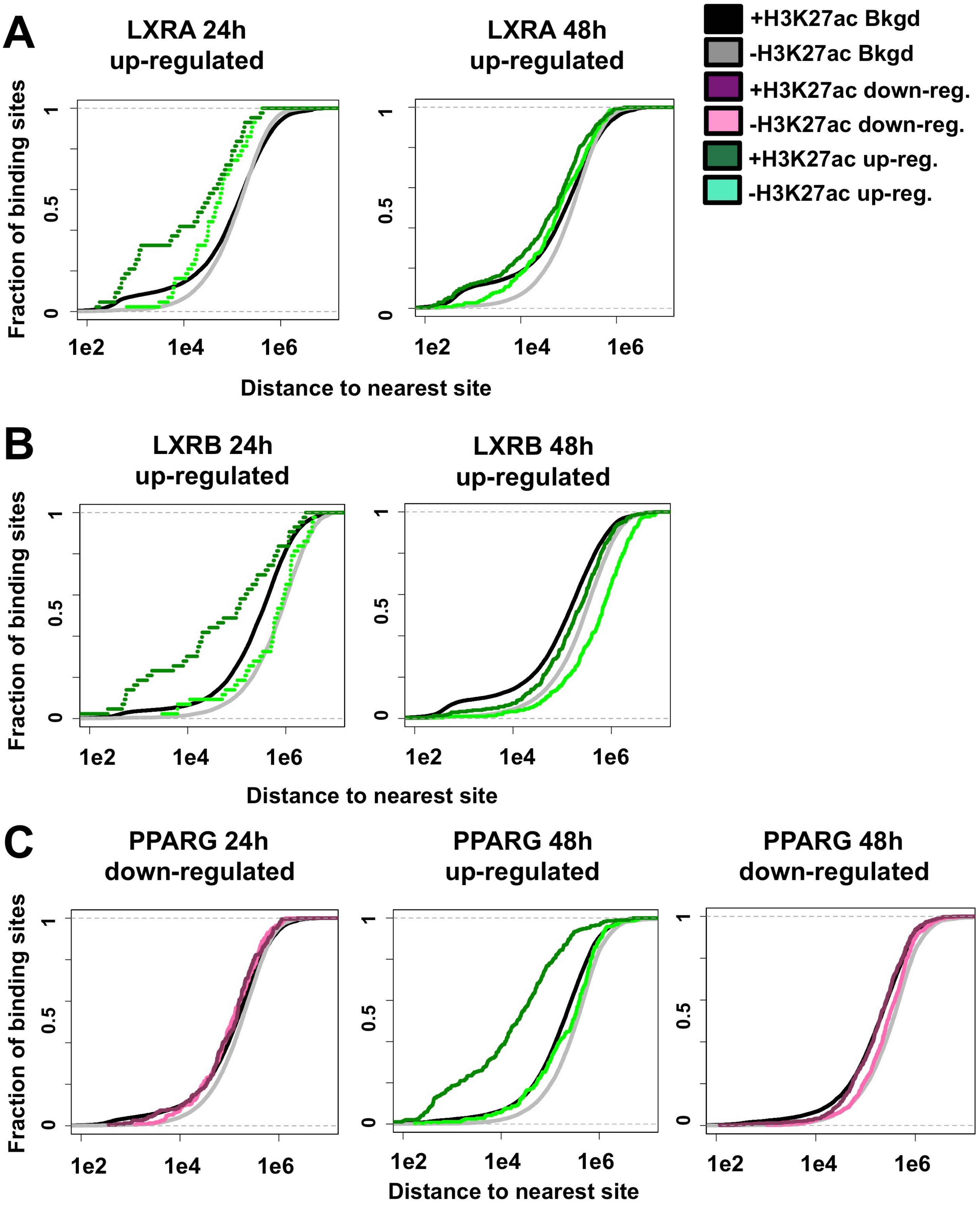
A
LXRA 24h up-regulated
LXRA 48h up-regulated
+H3K27ac Bkgd
-H3K27ac Bkgd
+H3K27ac down-reg.
-H3K27ac down-reg.
+H3K27ac up-reg.
-H3K27ac up-reg.
Fraction of binding sites
0
0.5
1
1e2
1e4
1e6
Distance to nearest site
B
LXRB 24h up-regulated
LXRB 48h up-regulated
C
PPARG 24h down-regulated
PPARG 48h up-regulated
PPARG 48h down-regulated

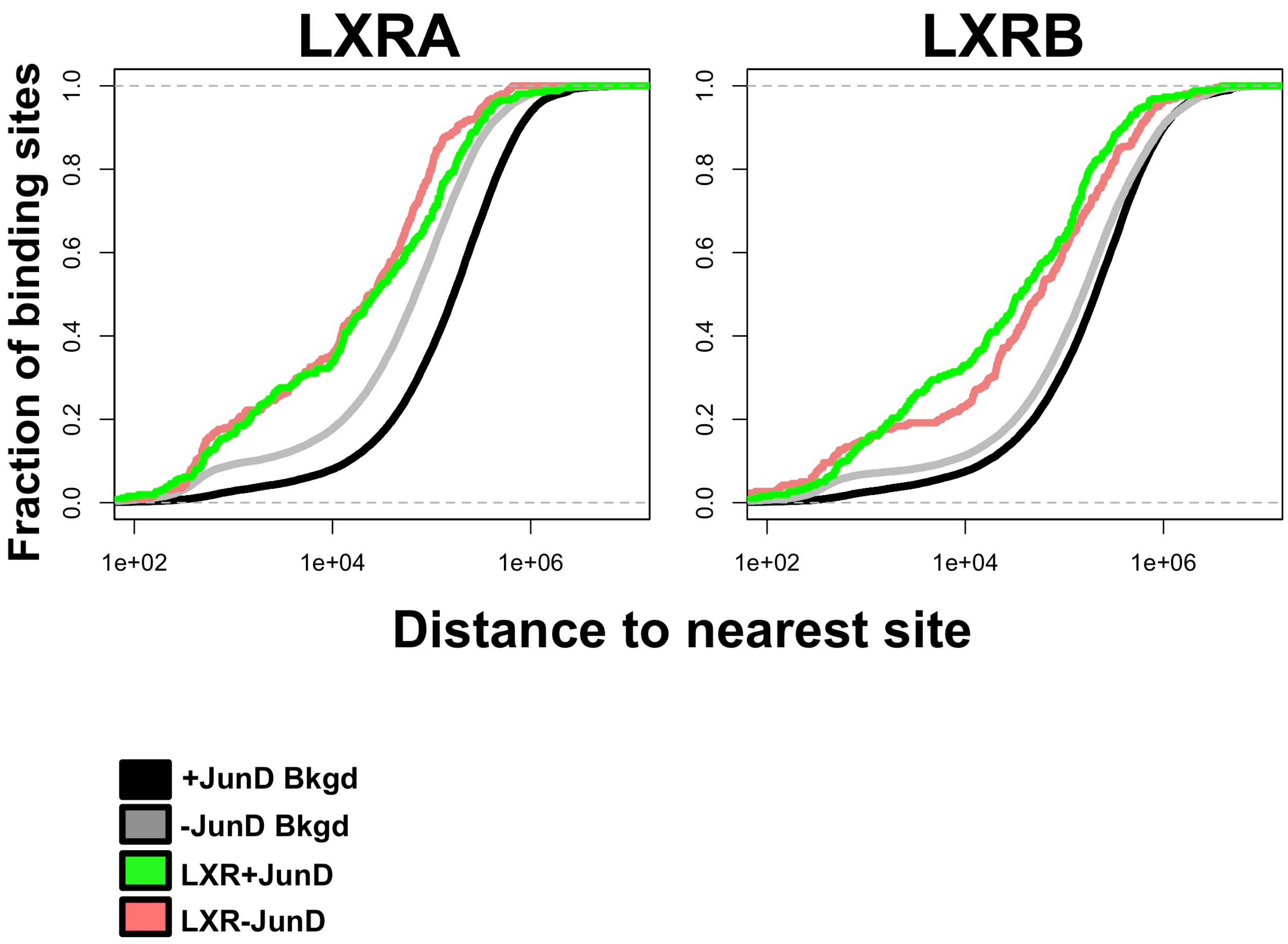
LXRA
LXRB
Fraction of binding sites
0.0
0.2
0.4
0.6
0.8
1.0
1e+02
1e+04
1e+06
Distance to nearest site
+JunD Bkgd
-JunD Bkgd
LXR+JunD
LXR-JunD

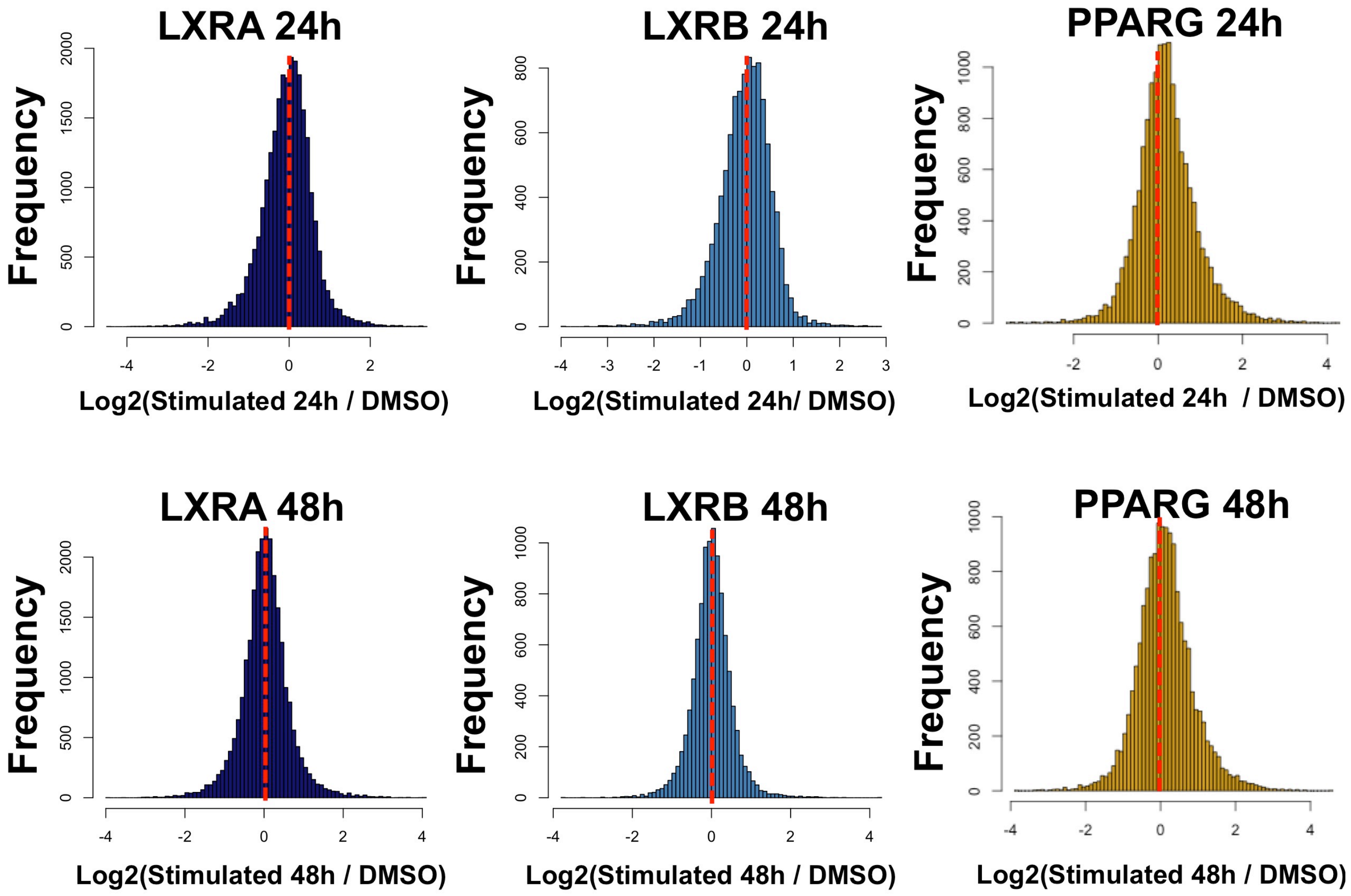

LXRA 24h
LXRB 24h
PPARG 24h
Frequency
Log2(Stimulated 24h / DMSO)
Log2(Stimulated 24h/ DMSO)
Log2(Stimulated 24h / DMSO)
LXRA 48h
LXRB 48h
PPARG 48h
Log2(Stimulated 48h / DMSO)
Log2(Stimulated 48h / DMSO)
Log2(Stimulated 48h / DMSO)

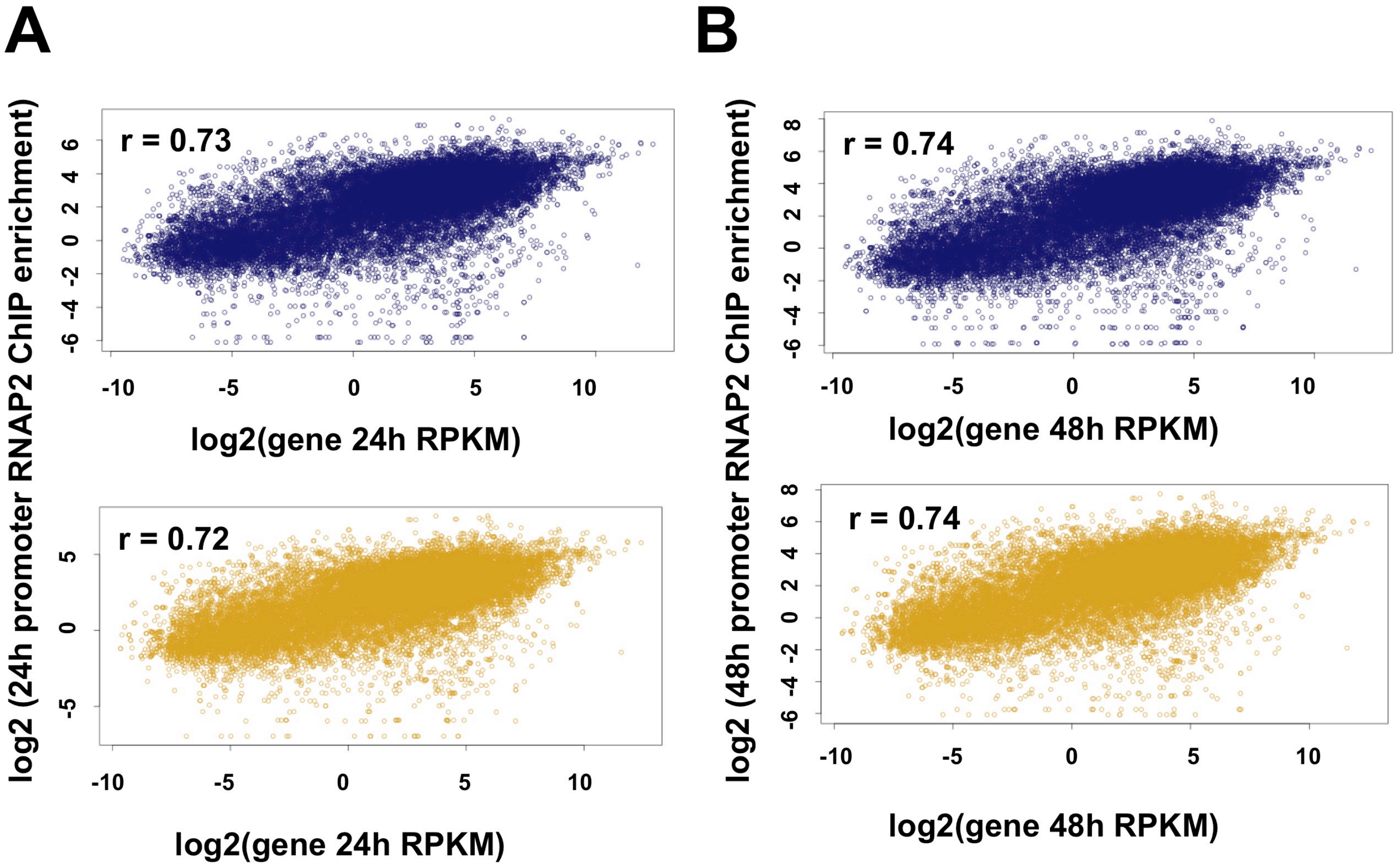
A
B
r = 0.73
r = 0.72
r = 0.74
r = 0.74
log2 (24h promoter RNAP2 ChIP enrichment)
log2 (48h promoter RNAP2 ChIP enrichment)
log2(gene 24h RPKM)
log2(gene 24h RPKM)
log2(gene 48h RPKM)
log2(gene 48h RPKM)

A

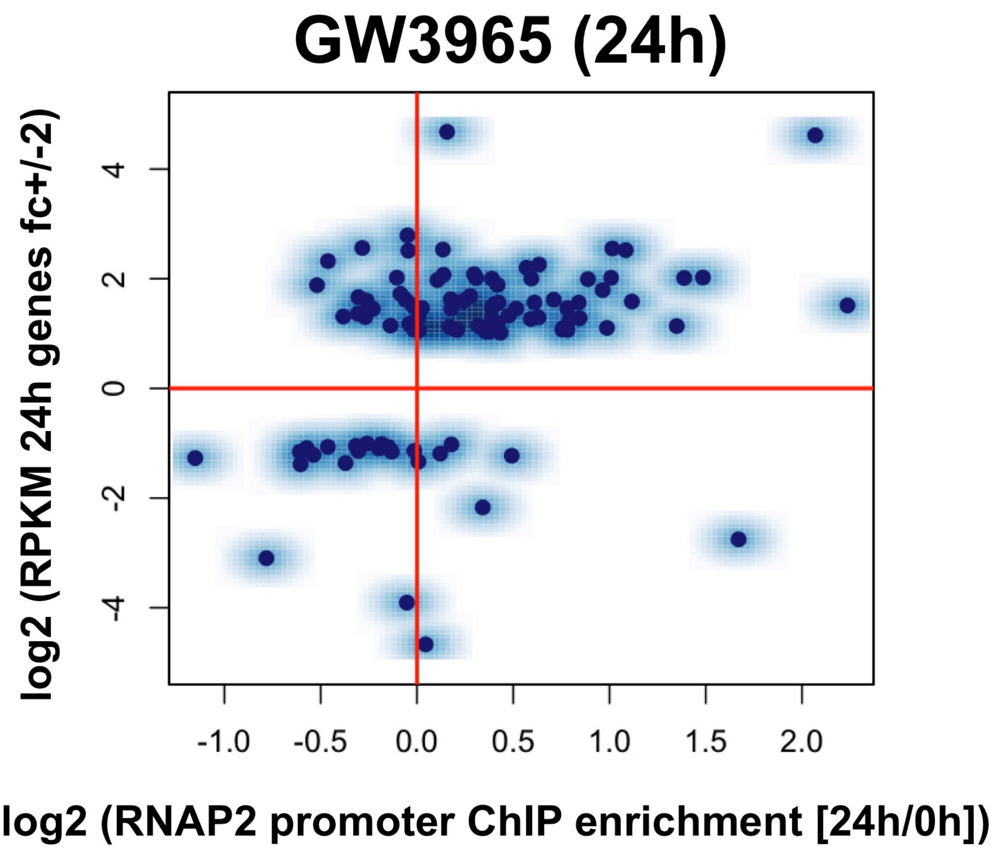
GW3965 (24h)
log2 (RPKM 24h genes fc+/-2)
4
2
0
-2
-4
-1.0
-0.5
0.0
0.5
1.0
1.5
2.0
log2 (RNAP2 promoter ChIP enrichment [24h/0h])

B

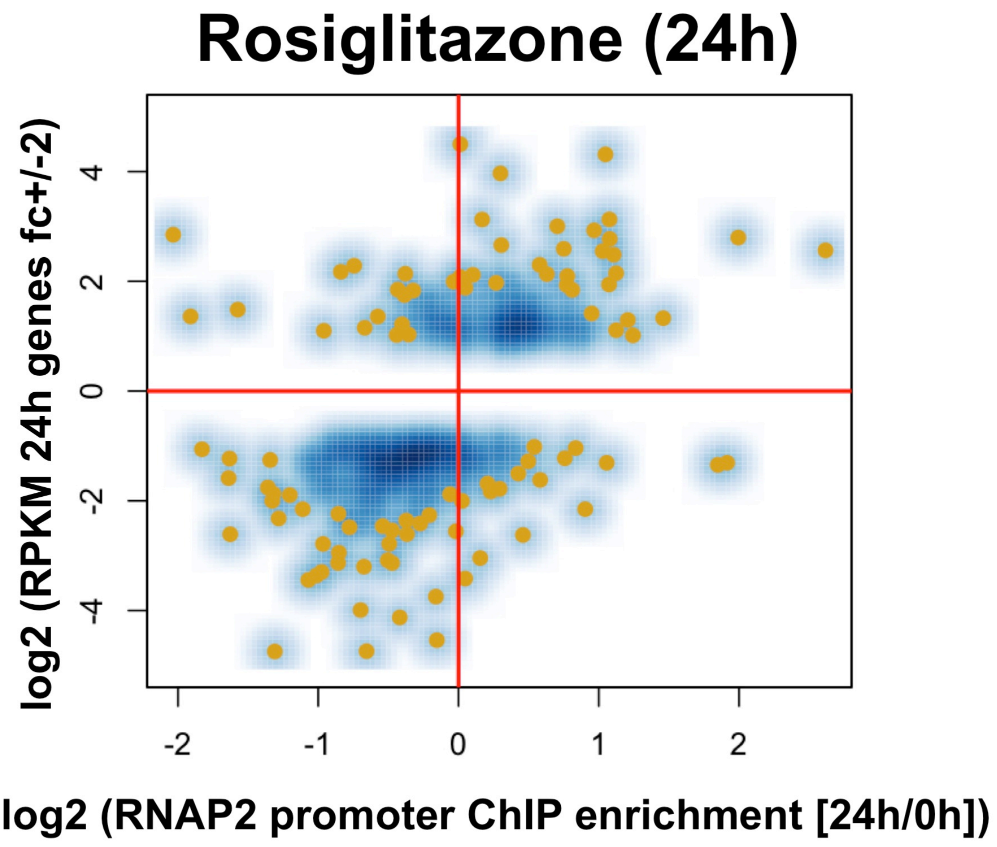
Rosiglitazone (24h)
log2 (RPKM 24h genes fc+/-2)
4
2
0
-2
-4
-2
-1
0
1
2
log2 (RNAP2 promoter ChIP enrichment [24h/0h])

A

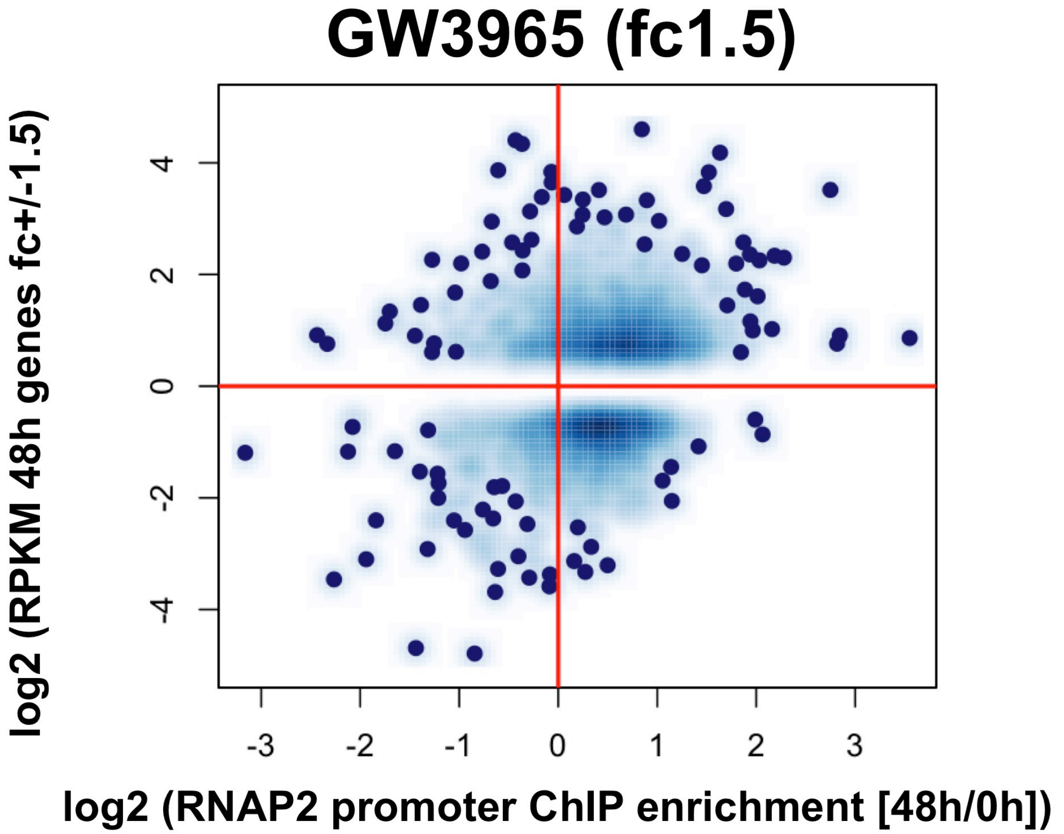
GW3965 (fc1.5)
log2 (RPKM 48h genes fc+/-1.5)
log2 (RNAP2 promoter ChIP enrichment [48h/0h])
4
2
0
-2
-4
-3
-2
-1
0
1
2
3

B

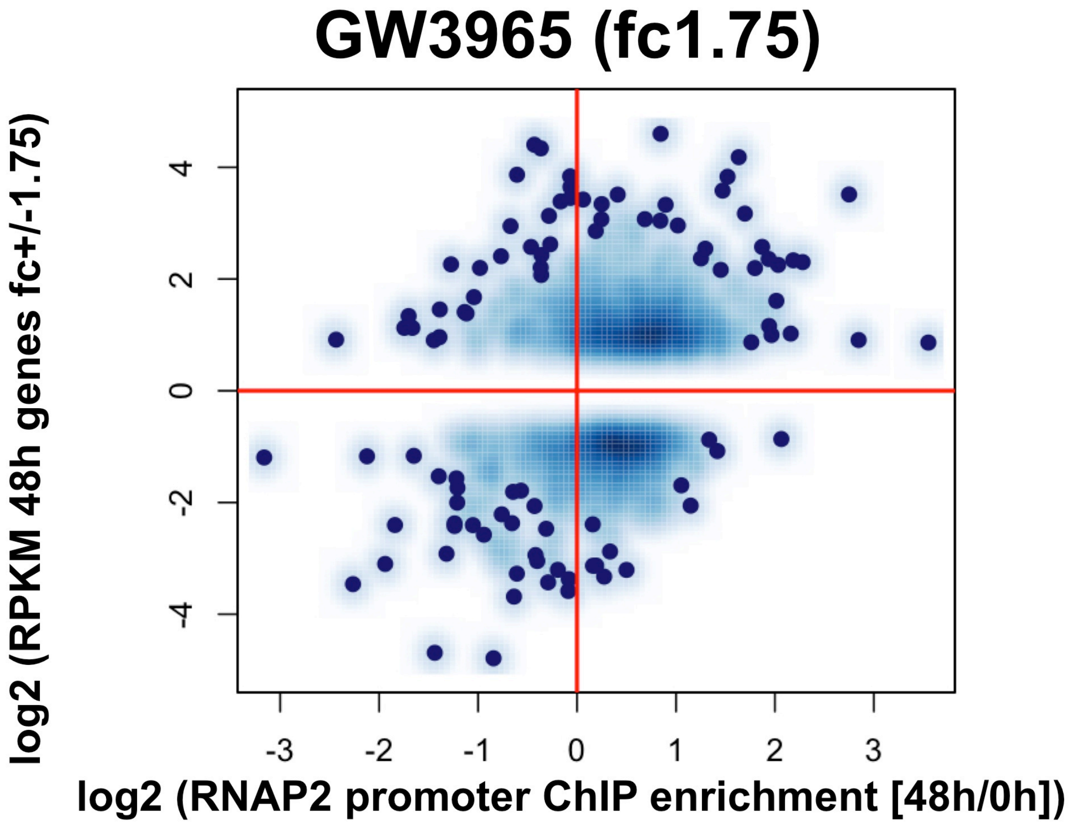
GW3965 (fc1.75)
log2 (RPKM 48h genes fc+/-1.75)
log2 (RNAP2 promoter ChIP enrichment [48h/0h])
4
2
0
-2
-4
-3
-2
-1
0
1
2
3

A

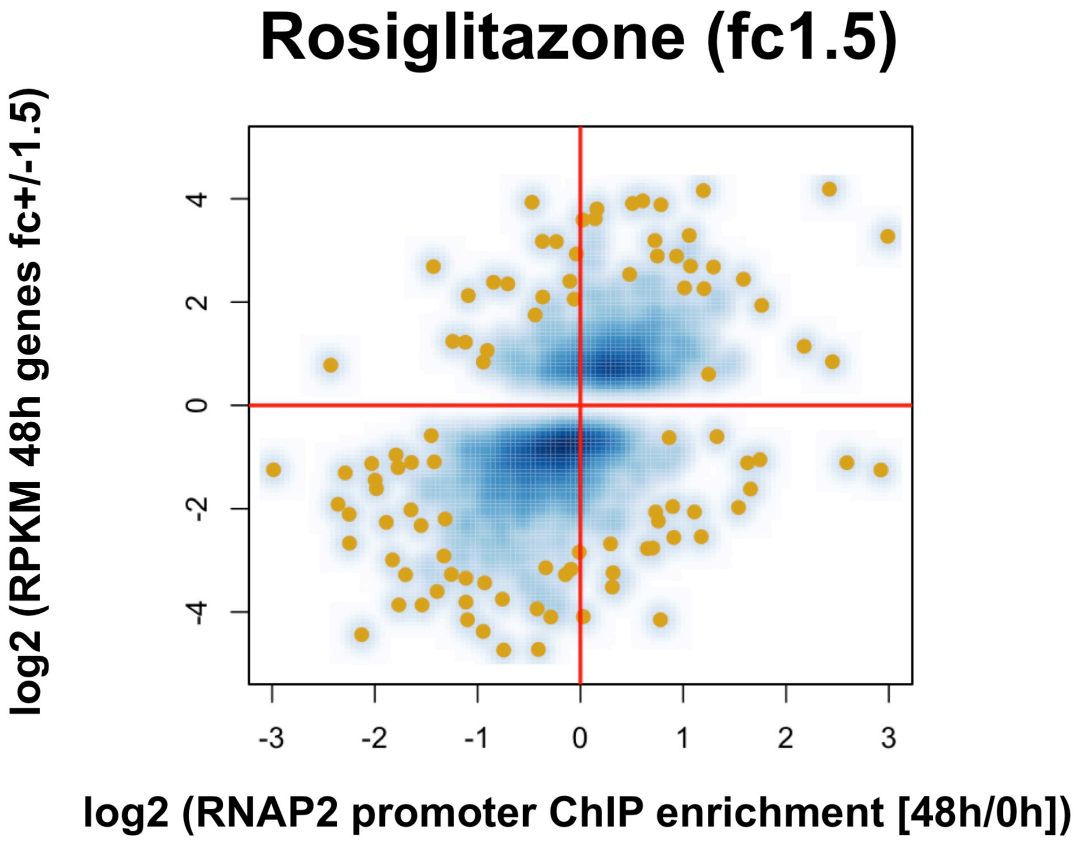
Rosiglitazone (fc1.5)
log2 (RPKM 48h genes fc+/-1.5)
4
2
0
-2
-4
-3
-2
-1
0
1
2
3
log2 (RNAP2 promoter ChIP enrichment [48h/0h])

B

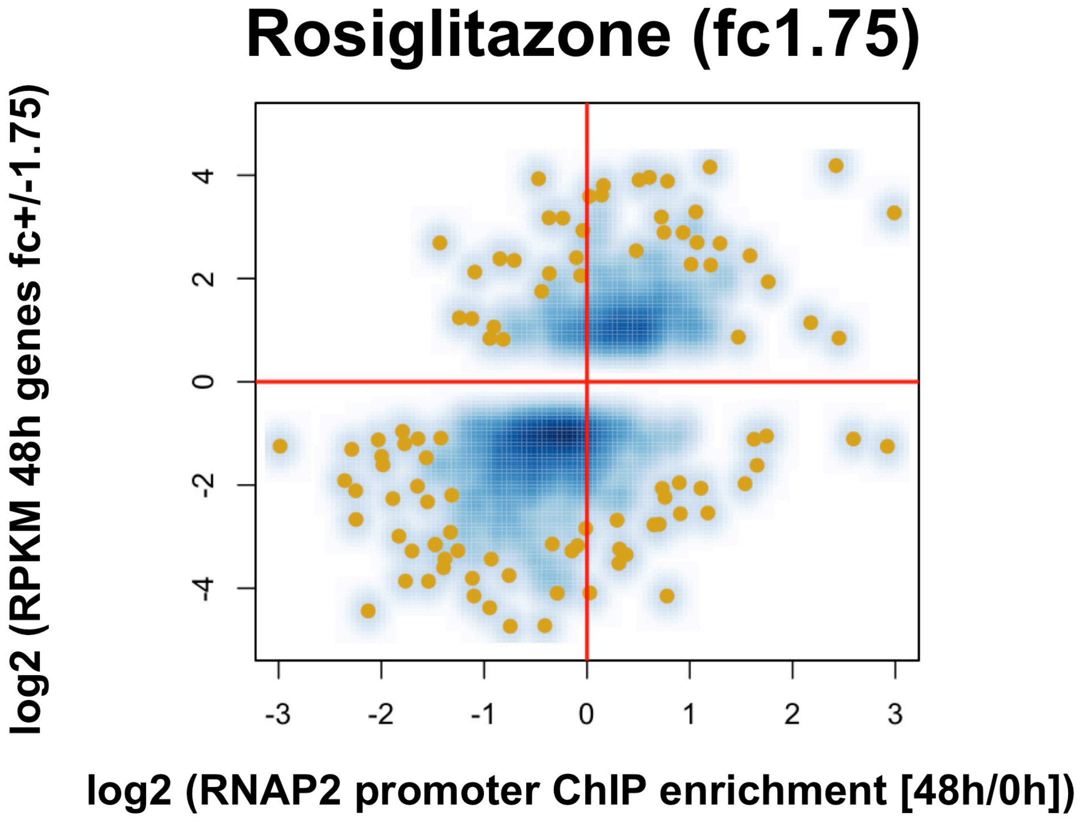
Rosiglitazone (fc1.75)
log2 (RPKM 48h genes fc+/-1.75)
4
2
0
-2
-4
-3
-2
-1
0
1
2
3
log2 (RNAP2 promoter ChIP enrichment [48h/0h])

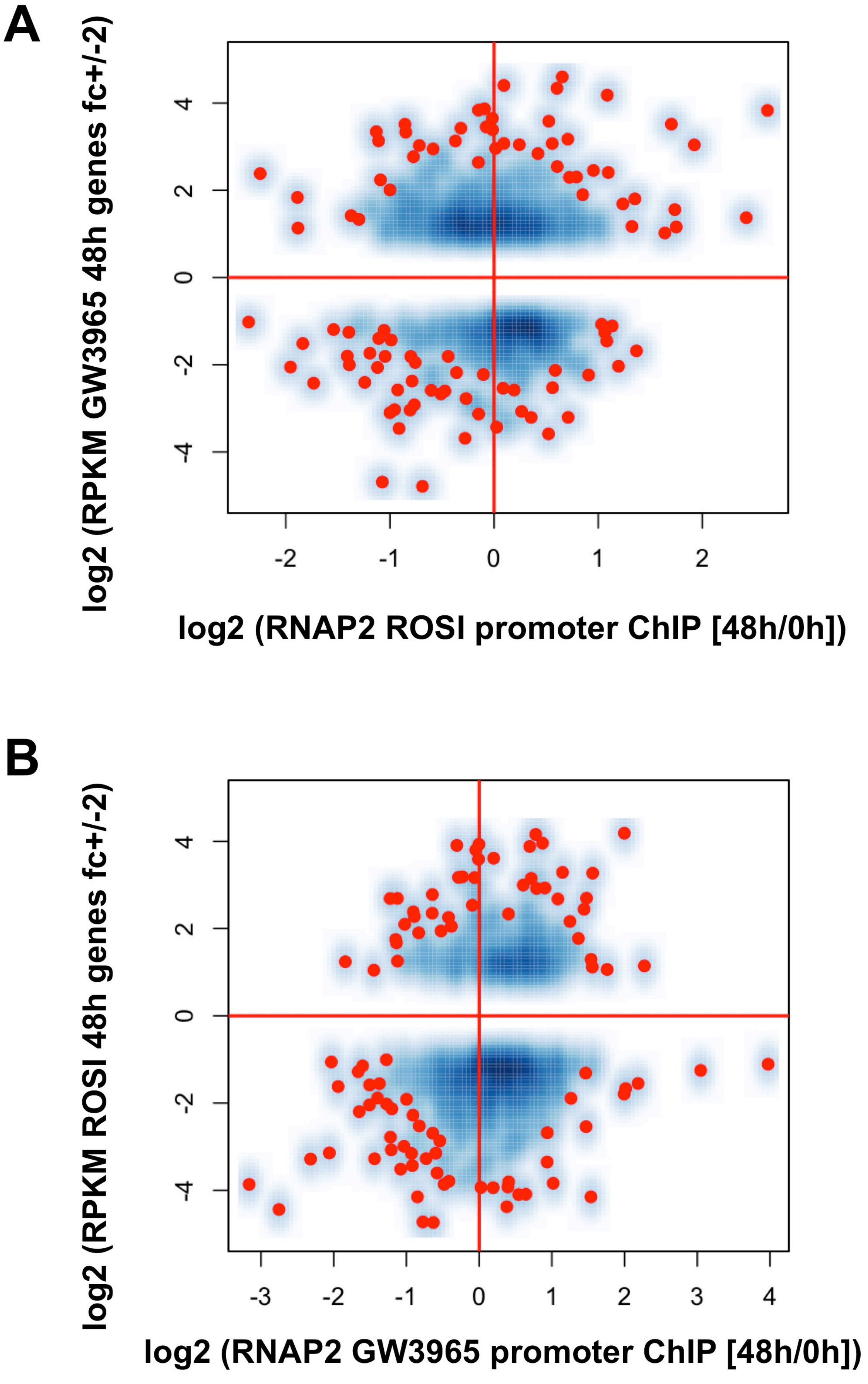
A
log2 (RPKM GW3965 48h genes fc+/-2)
4
2
0
-2
-4
-2
-1
0
1
2
log2 (RNAP2 ROSI promoter ChIP [48h/0h])
B
log2 (RPKM ROSI 48h genes fc+/-2)
4
2
0
-2
-4
-3
-2
-1
0
1
2
3
4
log2 (RNAP2 GW3965 promoter ChIP [48h/0h])

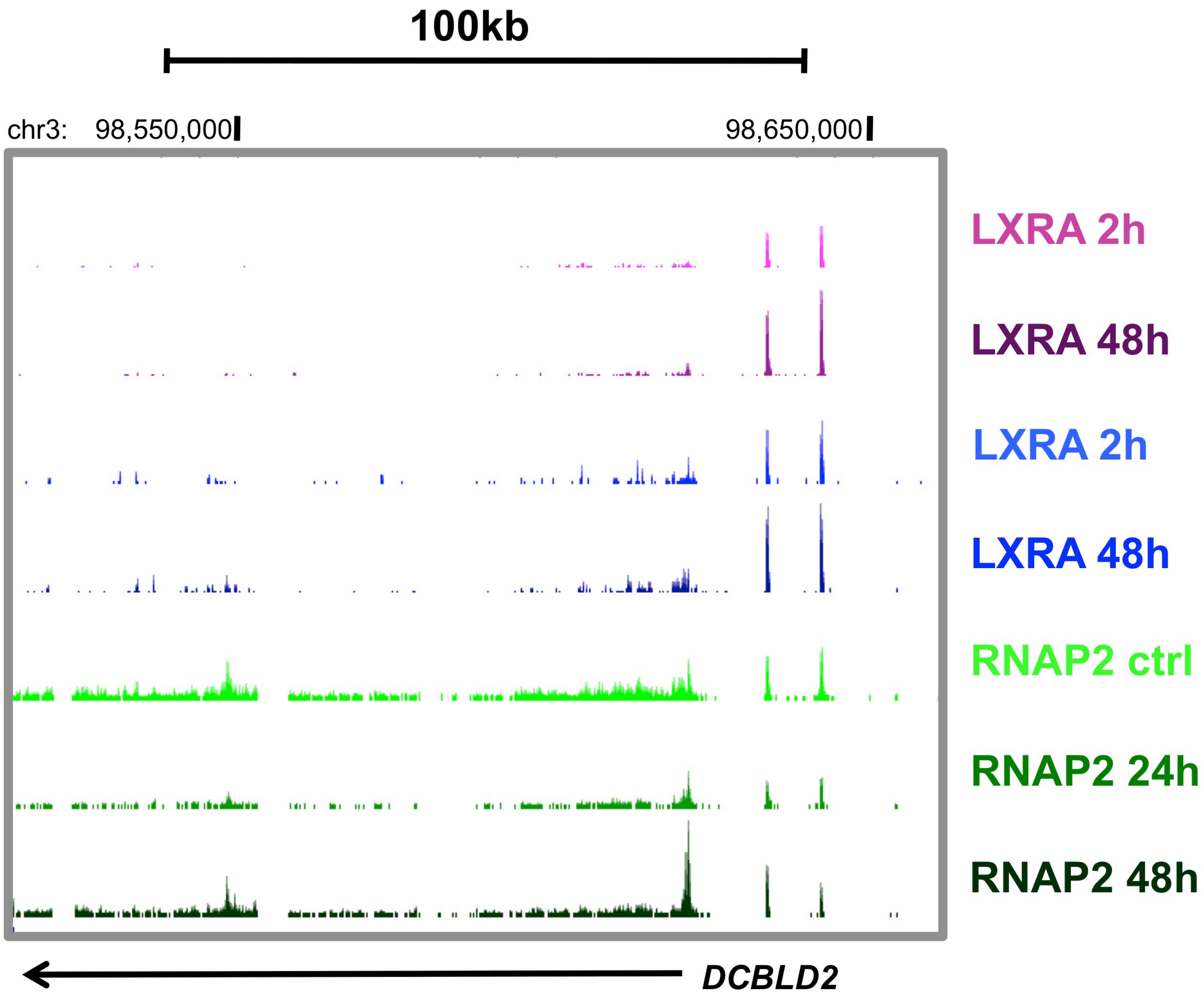
100kb
chr3: 98,550,000
98,650,000
LXRA 2h
LXRA 48h
LXRA 2h
LXRA 48h
RNAP2 ctrl
RNAP2 24h
RNAP2 48h
DCBLD2

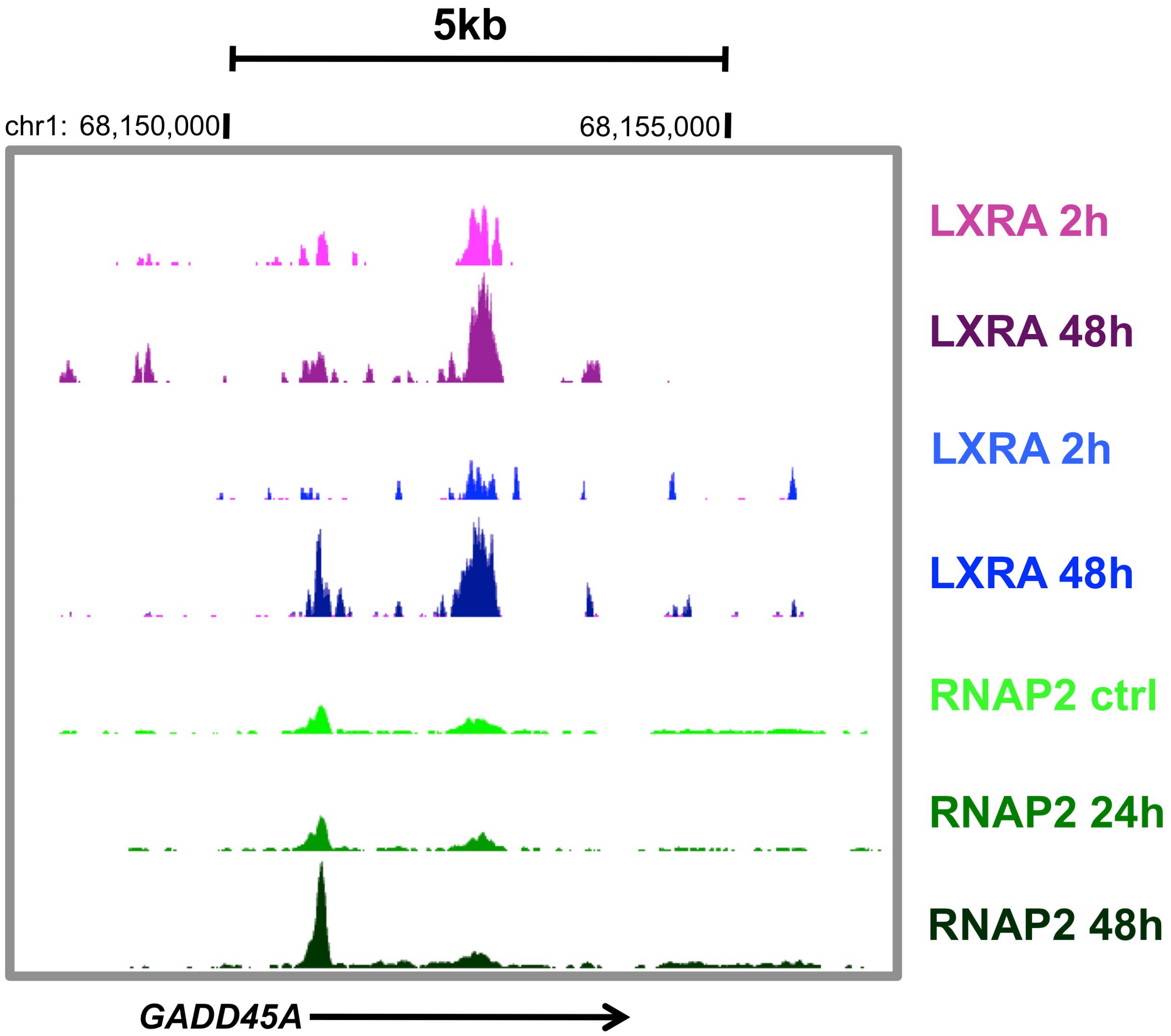
5kb
chr1: 68,150,000
68,155,000
LXRA 2h
LXRA 48h
LXRA 2h
LXRA 48h
RNAP2 ctrl
RNAP2 24h
RNAP2 48h
GADD45A

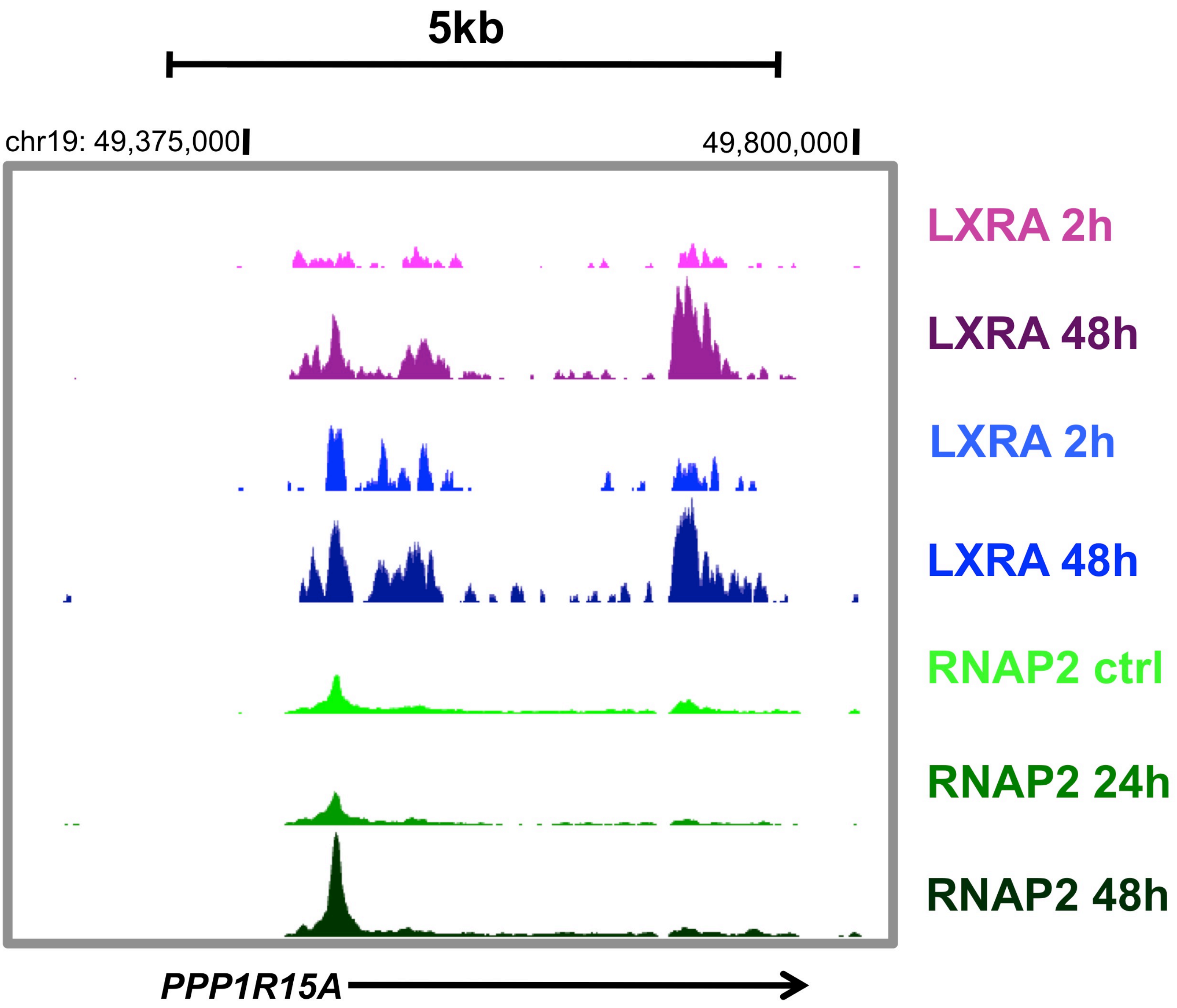
5kb
chr19: 49,375,000
49,800,000
LXRA 2h
LXRA 48h
LXRA 2h
LXRA 48h
RNAP2 ctrl
RNAP2 24h
RNAP2 48h
PPP1R15A

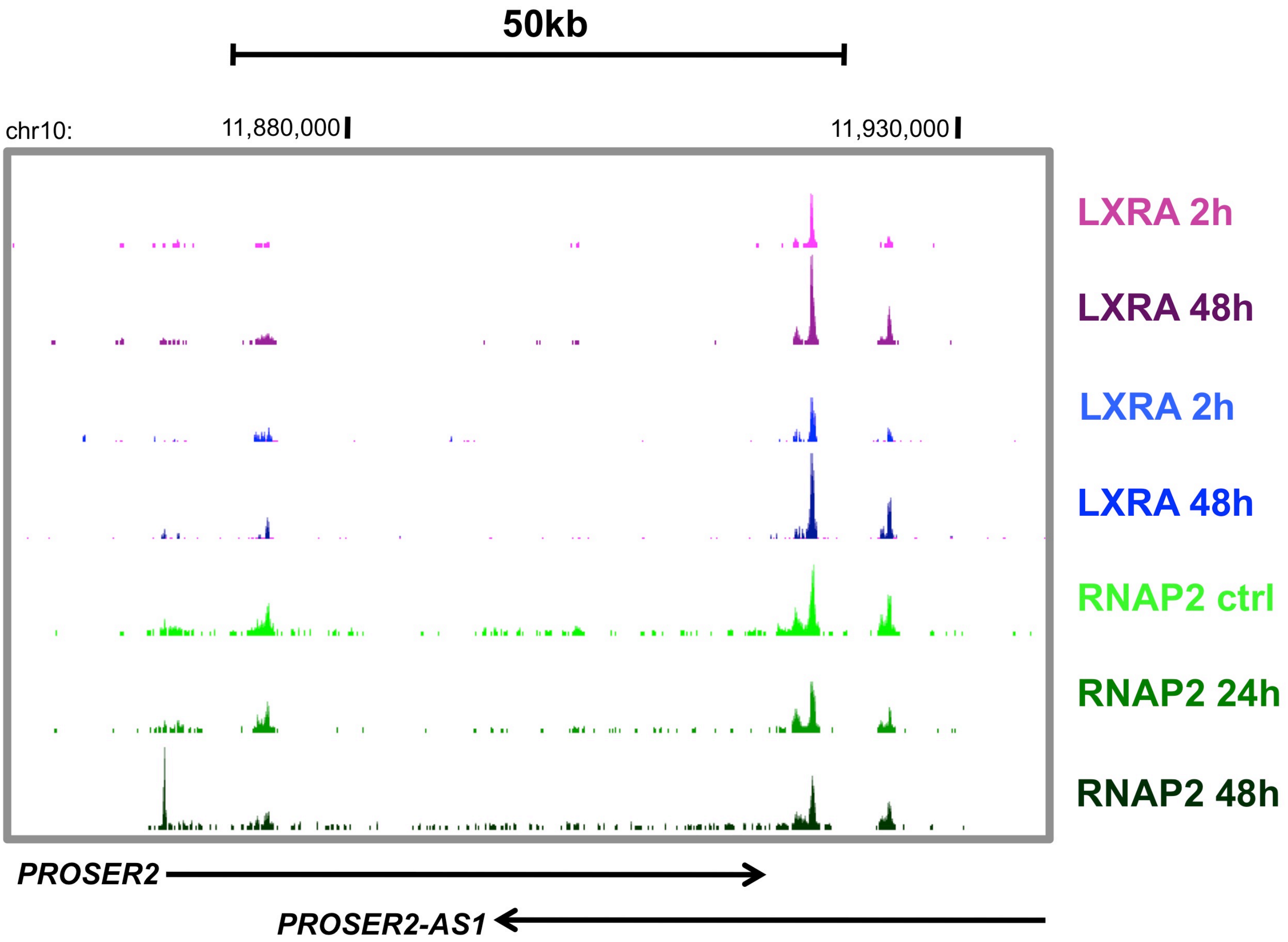
50kb
chr10:
11,880,000
11,930,000
LXRA 2h
LXRA 48h
LXRA 2h
LXRA 48h
RNAP2 ctrl
RNAP2 24h
RNAP2 48h
PROSER2
PROSER2-AS1

A

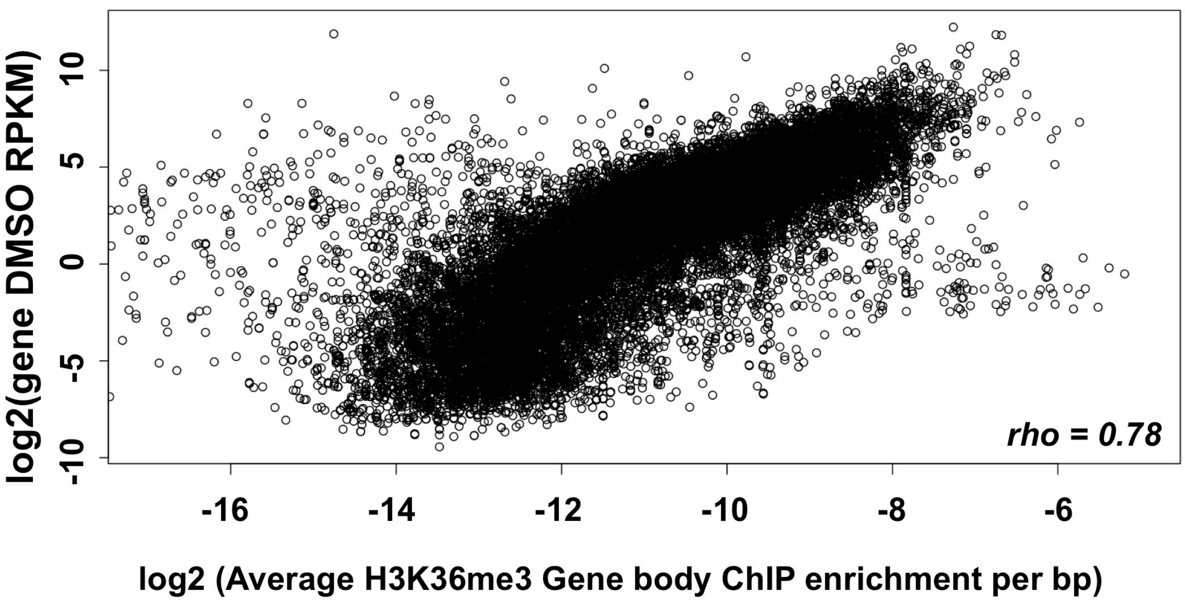
log2(gene DMSO RPKM)
10
5
0
-5
-10
rho = 0.78
-16
-14
-12
-10
-8
-6
log2 (Average H3K36me3 Gene body ChIP enrichment per bp)

B

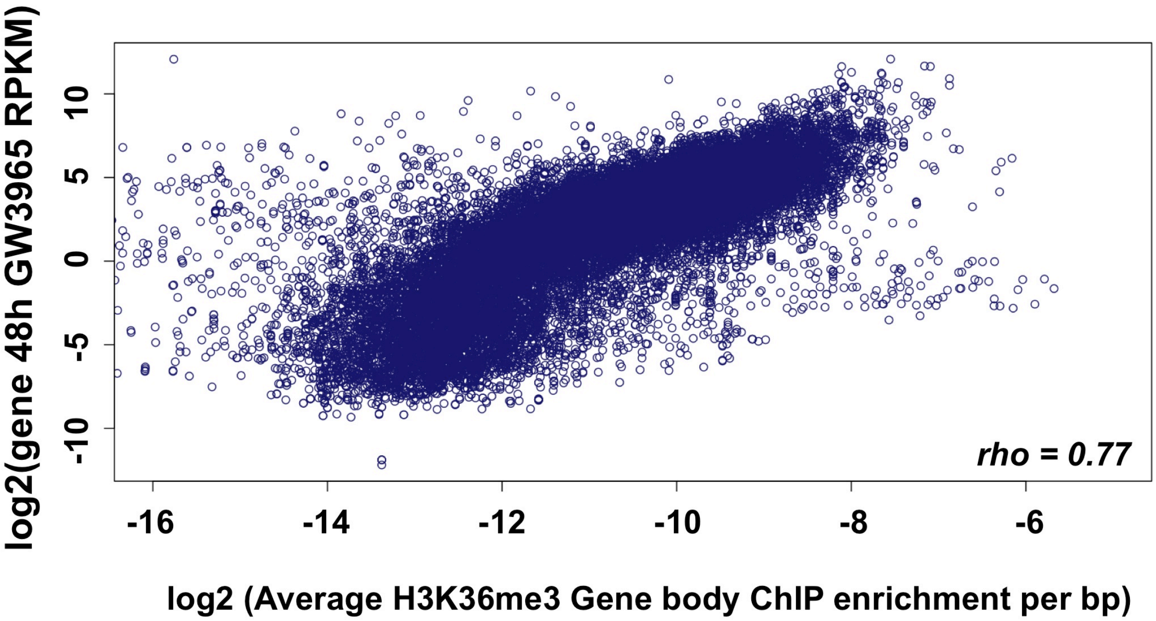
log2(gene 48h GW3965 RPKM)
10
5
0
-5
-10
rho = 0.77
-16
-14
-12
-10
-8
-6
log2 (Average H3K36me3 Gene body ChIP enrichment per bp)

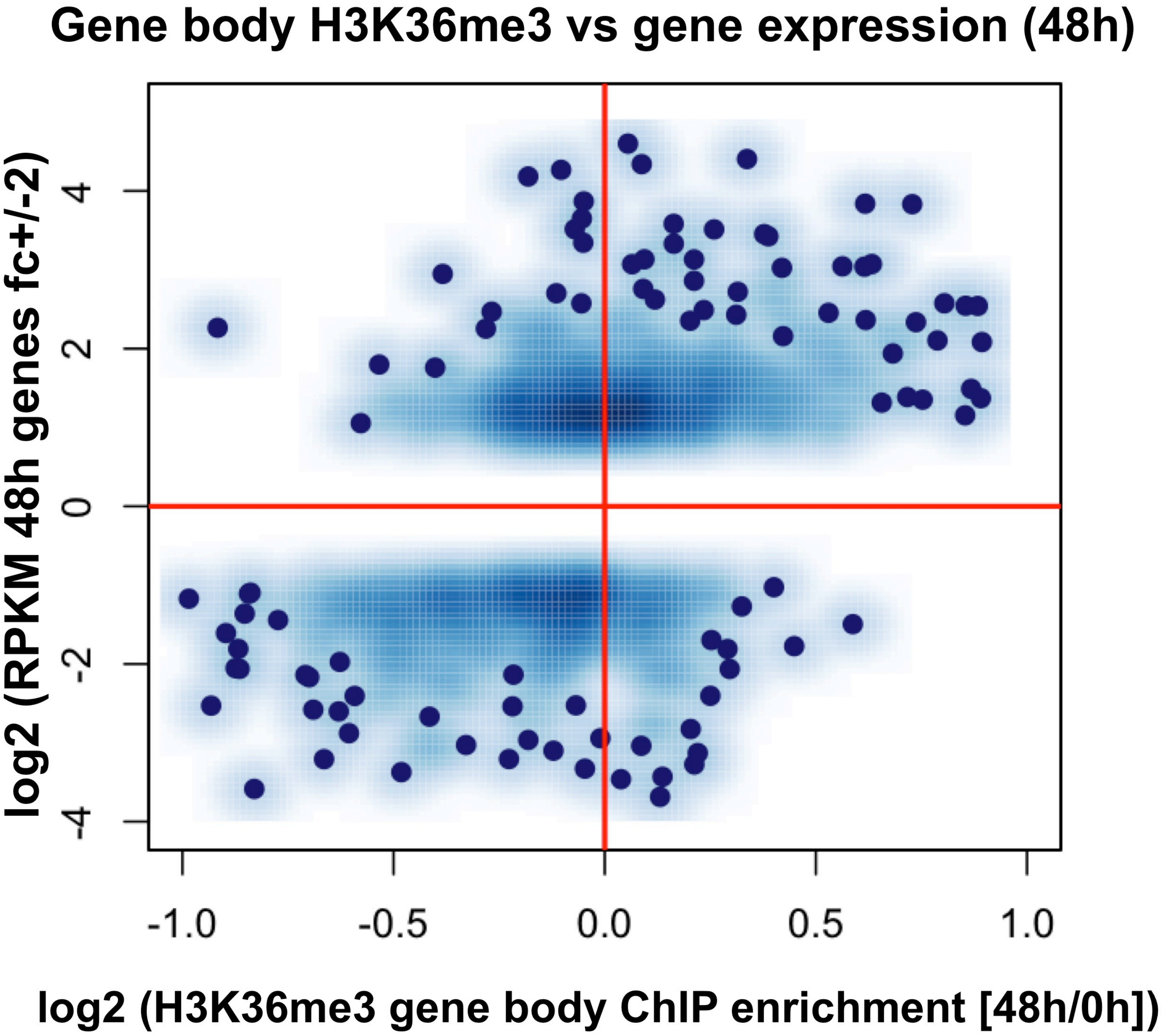
Gene body H3K36me3 vs gene expression (48h)
log2 (RPKM 48h genes fc+/-2)
4
2
0
-2
-4
-1.0
-0.5
0.0
0.5
1.0
log2 (H3K36me3 gene body ChIP enrichment [48h/0h])

A

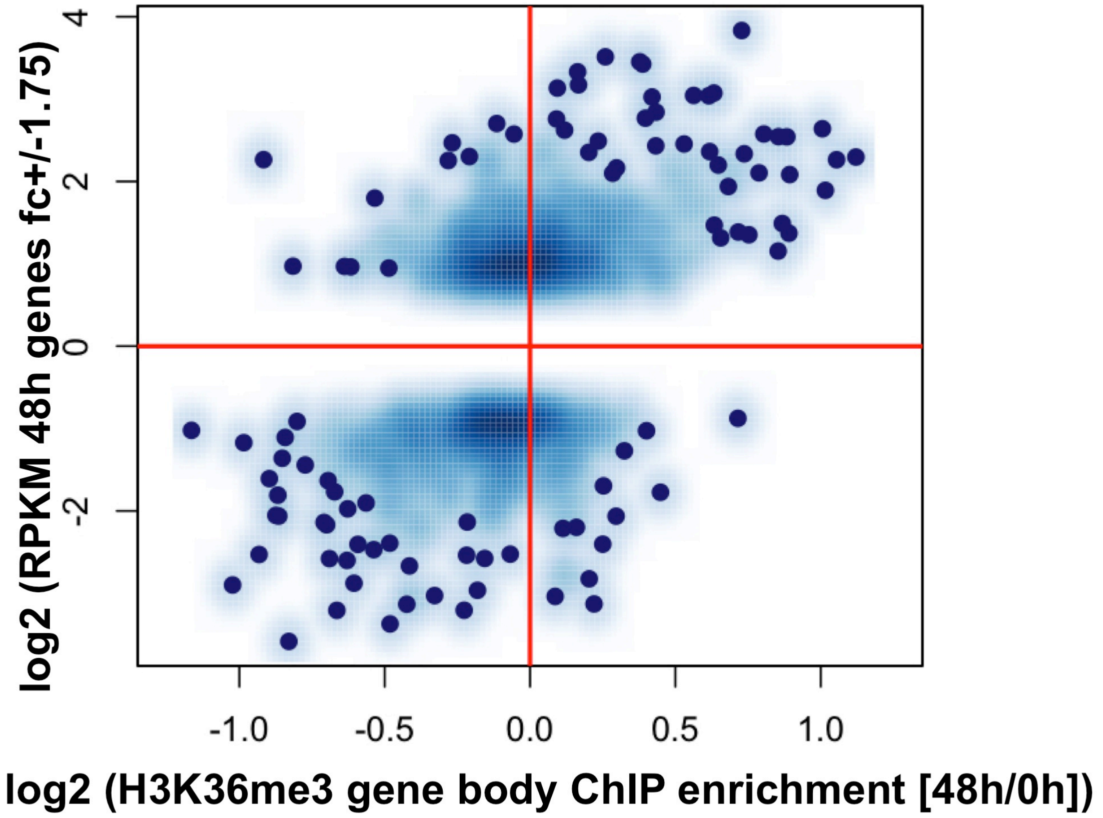
log2 (RPKM 48h genes fc+/-1.75)
4
2
0
-2
-1.0
-0.5
0.0
0.5
1.0
log2 (H3K36me3 gene body ChIP enrichment [48h/0h])

B

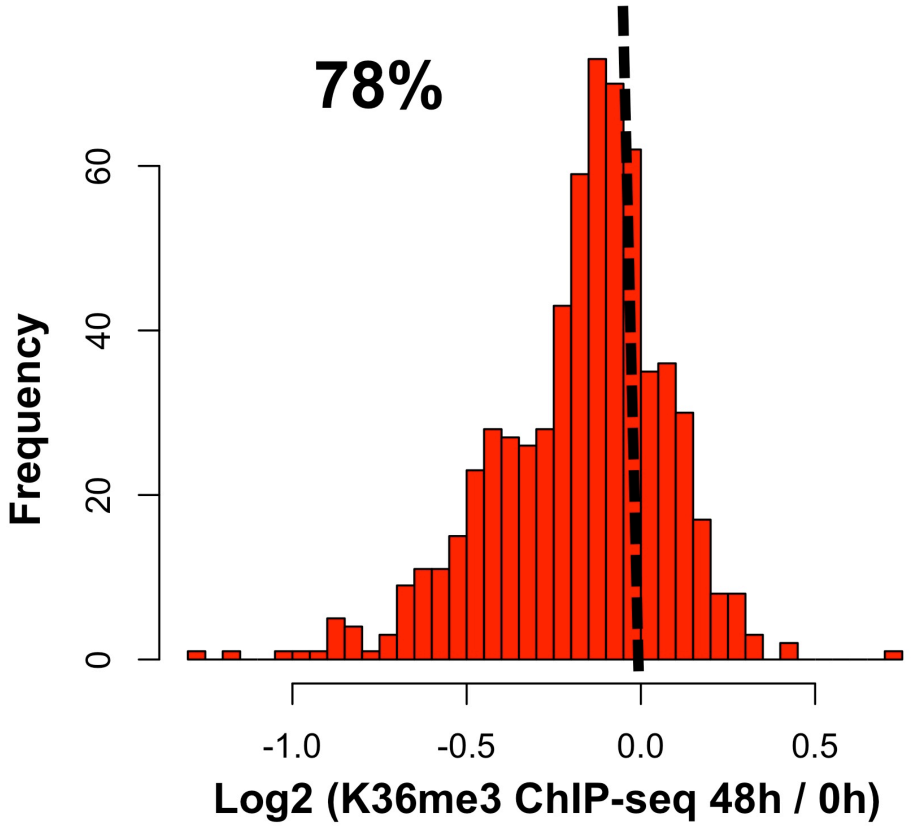
78%
Frequency
60
40
20
0
-1.0
-0.5
0.0
0.5
Log2 (K36me3 ChIP-seq 48h / 0h)

A

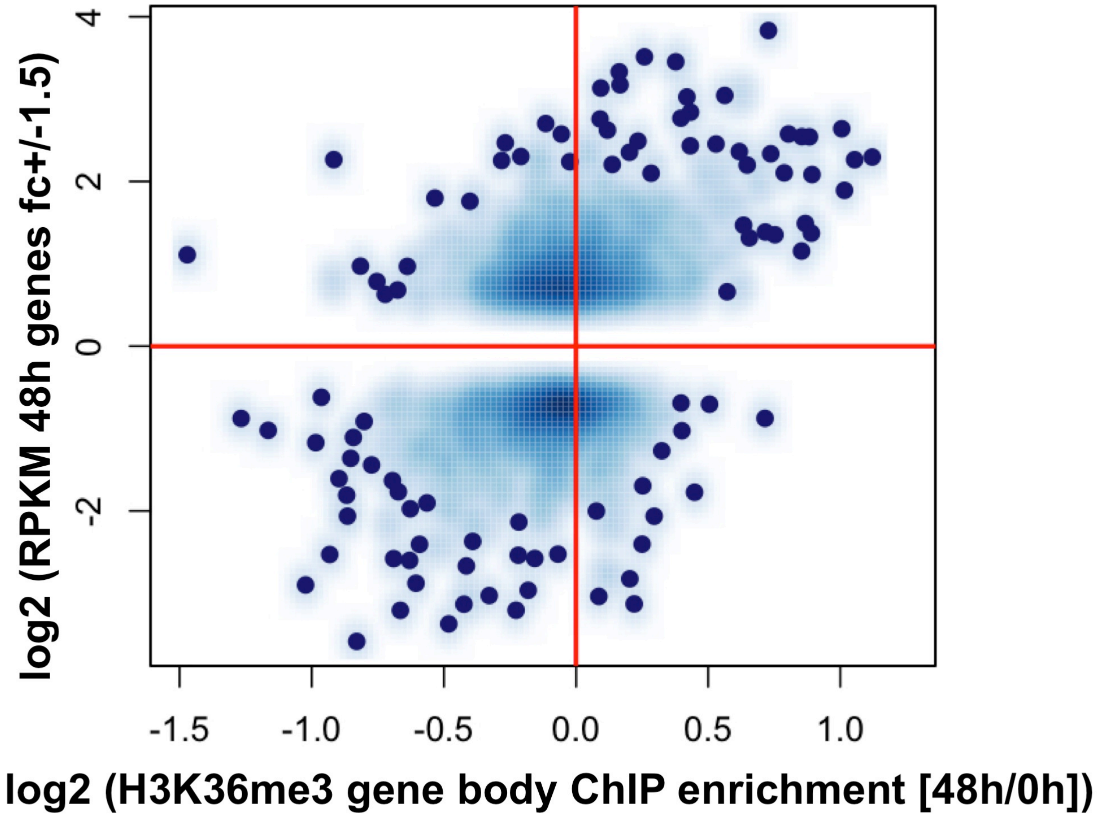

log2 (RPKM 48h genes fc+/-1.5)
4
2
0
-2
-1.5
-1.0
-0.5
0.0
0.5
1.0
log2 (H3K36me3 gene body ChIP enrichment [48h/0h])

B

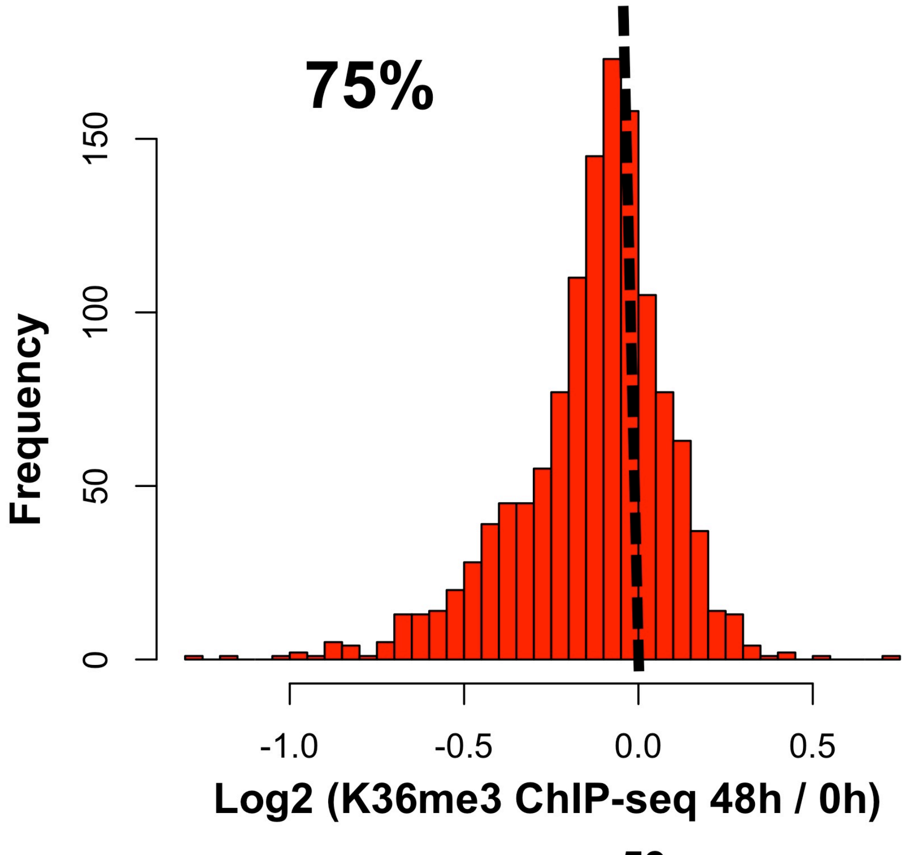

75%
Frequency
150
100
50
0
-1.0
-0.5
0.0
0.5
Log2 (K36me3 ChIP-seq 48h / 0h)

A

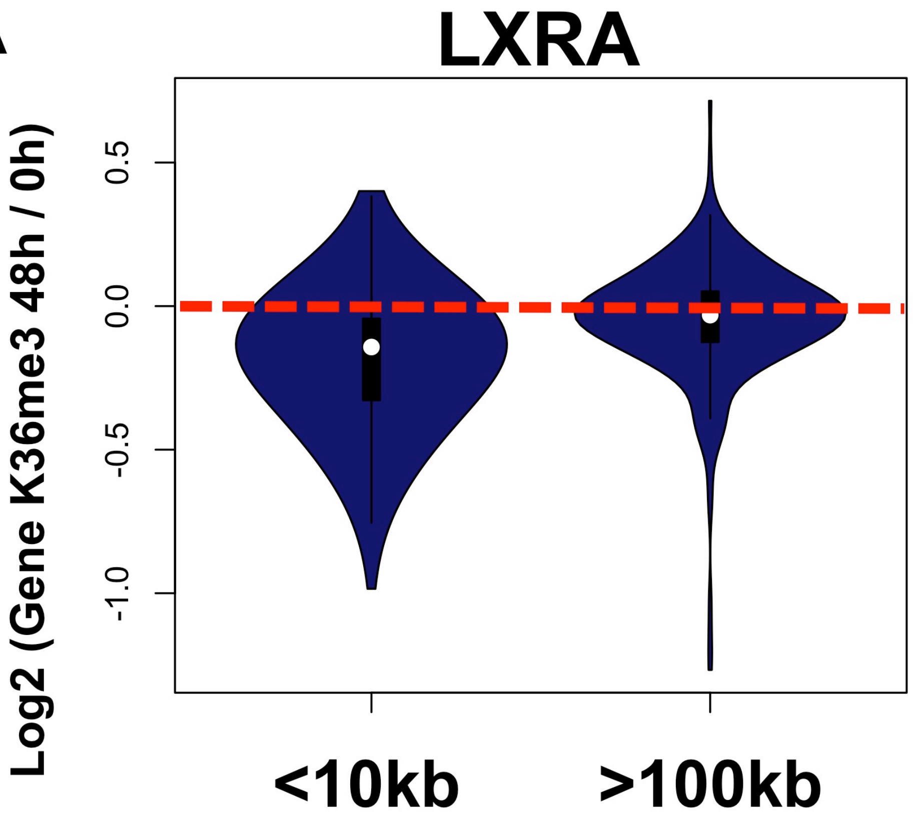
LXRA
Log2 (Gene K36me3 48h / 0h)
0.5
0.0
-0.5
-1.0
<10kb
>100kb

B

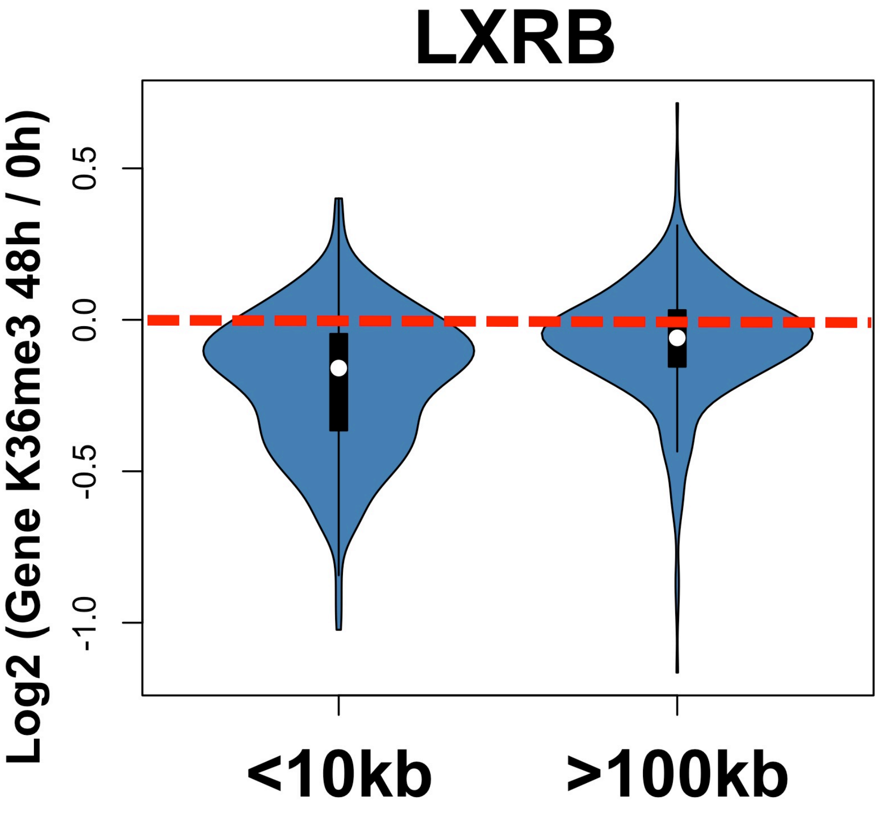
LXRB
Log2 (Gene K36me3 48h / 0h)
0.5
0.0
-0.5
-1.0
<10kb
>100kb
