# Supplementary material for: Distinct gene regulatory programs define the inhibitory effects of liver X receptors and PPARG on cancer cell proliferation
Source: Genome Med. 2016 Jul 11;8:74. doi: 10.1186/s13073-016-0328-6 (PMC4940857; doi:10.1186/s13073-016-0328-6)
Supplement: Additional file 2: Table S1. — (PDF 275 kb) [file 13073_2016_328_MOESM2_ESM.pdf]

TABLE S1

24h GW3965 up-regulated

Gene Set Name	# Genes	Description	# Genes in Overlap (k)	p-value	FDR q-value
KEGG_PPAR_SIGNALING_PATHWAY	69	PPAR signaling pathway	10	3.00E-13	5.58E-11
KEGG_FATTY_ACID_METABOLISM	42	Fatty acid metabolism	5	8.39E-07	6.60E-05
KEGG_ABC_TRANSPORTERS	44	ABC transporters	5	1.06E-06	6.60E-05
KEGG_ADIPOCYTOKINE_SIGNALING_PATHWAY	67	Adipocytokine signaling pathway	4	1.73E-04	8.07E-03
KEGG_INSULIN_SIGNALING_PATHWAY	137	Insulin signaling pathway	5	2.69E-04	1.00E-02
KEGG_PYRUVATE_METABOLISM	40	Pyruvate metabolism	3	6.05E-04	1.87E-02
KEGG_MTOR_SIGNALING_PATHWAY	52	mTOR signaling pathway	3	1.30E-03	3.45E-02
KEGG_GLYCOSPHINGOLIPID_BIOSYNTHESIS_GLOBO_SERIES	14	Glycosphingolipid biosynthesis - globo series	2	1.48E-03	3.45E-02
KEGG_GLYCOLYSIS_GLUCONEOGENESIS	62	Glycolysis / Gluconeogenesis	3	2.17E-03	4.48E-02
KEGG_PATHWAYS_IN_CANCER	328	Pathways in cancer	6	2.48E-03	4.55E-02
KEGG_COMPLEMENT_AND_COAGULATION_CASCADES	69	Complement and coagulation cascades	3	2.94E-03	4.55E-02
KEGG_P53_SIGNALING_PATHWAY	69	p53 signaling pathway	3	2.94E-03	4.55E-02
KEGG_VIRAL_MYOCARDITIS	73	Viral myocarditis	3	3.45E-03	4.89E-02
KEGG_BIOSYNTHESIS_OF_UNSATURATED_FATTY_ACIDS	22	Biosynthesis of unsaturated fatty acids	2	3.68E-03	4.89E-02
**24h GW3965 down-regulated**					
Gene Set Name	# Genes	Description	# Genes in Overlap (k)	p-value	FDR q-value
KEGG_LEUKOCYTE_TRANSENDOTHELIAL_MIGRATION	118	Leukocyte transendothelial migration	4	3.71E-05	6.91E-03
KEGG_VIBRIO_CHOLERAE_INFECTION	56	Vibrio cholerae infection	3	9.63E-05	8.96E-03
KEGG_GLYCOSAMINOGLYCAN_BIOSYNTHESIS_CHONDROITIN_[illegible]	22	Glycosaminoglycan biosynthesis - chondroitin sulfate	2	5.48E-04	3.40E-02
**24h T0901317 up-regulated**					
Gene Set Name	# Genes	Description	# Genes in Overlap (k)	p-value	FDR q-value
KEGG_PPAR_SIGNALING_PATHWAY	69	PPAR signaling pathway	6	5.92E-08	1.10E-05
KEGG_ABC_TRANSPORTERS	44	ABC transporters	4	8.82E-06	8.21E-04
KEGG_PEROXISOME	78	Peroxisome	4	8.58E-05	4.88E-03
KEGG_GLYCINE_SERINE_AND_THREONINE_METABOLISM	31	Glycine, serine and threonine metabolism	3	1.05E-04	4.88E-03
KEGG_PYRUVATE_METABOLISM	40	Pyruvate metabolism	3	2.26E-04	8.41E-03
KEGG_MTOR_SIGNALING_PATHWAY	52	mTOR signaling pathway	3	4.93E-04	1.53E-02
KEGG_INSULIN_SIGNALING_PATHWAY	137	Insulin signaling pathway	4	7.39E-04	1.92E-02
KEGG_GLYCOLYSIS_GLUCONEOGENESIS	62	Glycolysis / Gluconeogenesis	3	8.26E-04	1.92E-02
KEGG_ADIPOCYTOKINE_SIGNALING_PATHWAY	67	Adipocytokine signaling pathway	3	1.04E-03	2.14E-02
KEGG_BIOSYNTHESIS_OF_UNSATURATED_FATTY_ACIDS	22	Biosynthesis of unsaturated fatty acids	2	1.90E-03	3.52E-02
KEGG_NITROGEN_METABOLISM	23	Nitrogen metabolism	2	2.08E-03	3.52E-02
KEGG_PATHWAYS_IN_CANCER	328	Pathways in cancer	5	2.93E-03	4.53E-02
**24h T0901317 down-regulated**					
Gene Set Name	# Genes	Description	# Genes in Overlap (k)	p-value	FDR q-value
KEGG_DILATED_CARDIOMYOPATHY	92	Dilated cardiomyopathy	2	1.10E-04	2.05E-02

**24h Rosiglitazone up-regulated**					
Gene Set Name	# Genes	Description	# Genes in Overlap (k)	p-value	FDR q-value
KEGG_RETINOL_METABOLISM	64	Retinol metabolism	12	4.03E-14	7.49E-12
KEGG_ASCORBATE_AND_ALDARATE_METABOLISM	25	Ascorbate and aldarate metabolism	8	6.79E-12	6.31E-10
KEGG_PORPHYRIN_AND_CHLOROPHYLL_METABOLISM	41	Porphyrin and chlorophyll metabolism	9	1.41E-11	8.76E-10
KEGG_PPAR_SIGNALING_PATHWAY	69	PPAR signaling pathway	10	8.12E-11	3.78E-09
KEGG_DRUG_METABOLISM_OTHER_ENZYMES	51	Drug metabolism - other enzymes	9	1.15E-10	4.28E-09
KEGG_STARCH_AND_SUCROSE_METABOLISM	52	Starch and sucrose metabolism	9	1.38E-10	4.29E-09
KEGG_PENTOSE_AND_GLUCURONATE_INTERCONVERSIONS	28	Pentose and glucuronate interconversions	7	1.02E-09	2.72E-08
KEGG_DRUG_METABOLISM_CYTOCHROME_P450	72	Drug metabolism - cytochrome P450	9	2.82E-09	6.55E-08
KEGG_STEROID_HORMONE_BIOSYNTHESIS	55	Steroid hormone biosynthesis	8	6.33E-09	1.31E-07
KEGG_METABOLISM_OF_XENOBIOTICS_BY_CYTOCHROME_P45	70	Metabolism of xenobiotics by cytochrome P450	8	4.46E-08	8.30E-07
KEGG_BUTANOATE_METABOLISM	34	Butanoate metabolism	6	1.57E-07	2.65E-06
KEGG_CALCIUM_SIGNALING_PATHWAY	178	Calcium signaling pathway	9	6.81E-06	1.06E-04
KEGG_FATTY_ACID_METABOLISM	42	Fatty acid metabolism	5	1.32E-05	1.89E-04
KEGG_GLYCEROPHOSPHOLIPID_METABOLISM	77	Glycerophospholipid metabolism	6	2.13E-05	2.83E-04
KEGG_PATHWAYS_IN_CANCER	328	Pathways in cancer	11	3.21E-05	3.97E-04
KEGG_GNRH_SIGNALING_PATHWAY	101	GnRH signaling pathway	6	9.84E-05	1.14E-03
KEGG_FOCAL_ADHESION	201	Focal adhesion	8	1.20E-04	1.31E-03
KEGG_VASCULAR_SMOOTH_MUSCLE_CONTRACTION	115	Vascular smooth muscle contraction	6	2.01E-04	2.07E-03
KEGG_VALINE_LEUCINE_AND_ISOLEUCINE_DEGRADATION	44	Valine, leucine and isoleucine degradation	4	2.92E-04	2.86E-03
KEGG_TGF_BETA_SIGNALING_PATHWAY	86	TGF-beta signaling pathway	5	4.18E-04	3.88E-03
KEGG_DILATED_CARDIOMYOPATHY	92	Dilated cardiomyopathy	5	5.69E-04	5.04E-03
KEGG_HEDGEHOG_SIGNALING_PATHWAY	56	Hedgehog signaling pathway	4	7.39E-04	6.25E-03
KEGG_MAPK_SIGNALING_PATHWAY	267	MAPK signaling pathway	8	7.97E-04	6.45E-03
KEGG_ARACHIDONIC_ACID_METABOLISM	58	Arachidonic acid metabolism	4	8.44E-04	6.54E-03
KEGG_LINOLEIC_ACID_METABOLISM	29	Linoleic acid metabolism	3	1.20E-03	8.91E-03
KEGG_FRUCTOSE_AND_MANNOSE_METABOLISM	34	Fructose and mannose metabolism	3	1.91E-03	1.37E-02
KEGG_LIMONENE_AND_PINENE_DEGRADATION	10	Limonene and pinene degradation	2	2.27E-03	1.53E-02
KEGG_VEGF_SIGNALING_PATHWAY	76	VEGF signaling pathway	4	2.31E-03	1.53E-02
KEGG_AXON_GUIDANCE	129	Axon guidance	5	2.57E-03	1.65E-02
KEGG_TRYPTOPHAN_METABOLISM	40	Tryptophan metabolism	3	3.05E-03	1.89E-02
KEGG_ECM_RECEPTOR_INTERACTION	84	ECM-receptor interaction	4	3.31E-03	1.99E-02
KEGG_CIRCADIAN_RHYTHM_MAMMAL	13	Circadian rhythm - mammal	2	3.87E-03	2.19E-02
KEGG_AMINO_SUGAR_AND_NUCLEOTIDE_SUGAR_METABOLISN	44	Amino sugar and nucleotide sugar metabolism	3	4.01E-03	2.19E-02
KEGG_VASOPRESSIN_REGULATED_WATER_REABSORPTION	44	Vasopressin-regulated water reabsorption	3	4.01E-03	2.19E-02
KEGG_WNT_SIGNALING_PATHWAY	151	Wnt signaling pathway	5	5.02E-03	2.67E-02
KEGG_GLYCEROLIPID_METABOLISM	49	Glycerolipid metabolism	3	5.43E-03	2.81E-02
KEGG_ALPHA_LINOLENIC_ACID_METABOLISM	19	alpha-Linolenic acid metabolism	2	8.25E-03	4.15E-02
KEGG_ACUTE_MYELOID_LEUKEMIA	60	Acute myeloid leukemia	3	9.51E-03	4.62E-02
KEGG_OOCYTE_MEIOSIS	114	Oocyte meiosis	4	9.68E-03	4.62E-02
KEGG_COLORECTAL_CANCER	62	Colorectal cancer	3	1.04E-02	4.72E-02
KEGG_GLYCOLYSIS_GLUCONEOGENESIS	62	Glycolysis / Gluconeogenesis	3	1.04E-02	4.72E-02
KEGG_BETA_ALANINE_METABOLISM	22	beta-Alanine metabolism	2	1.10E-02	4.87E-02

**24h Rosiglitazone down-regulated**					
Gene Set Name	# Genes	Description	# Genes in Overlap (k)	p-value	FDR q-value
KEGG_TAURINE_AND_HYPOTAURINE_METABOLISM	10	Taurine and hypotaurine metabolism	5	6.44E-08	1.20E-05
KEGG_MAPK_SIGNALING_PATHWAY	267	MAPK signaling pathway	14	6.44E-06	5.99E-04
KEGG_O_GLYCAN_BIOSYNTHESIS	30	O-Glycan biosynthesis	5	2.97E-05	1.49E-03
KEGG_AXON_GUIDANCE	129	Axon guidance	9	3.21E-05	1.49E-03
KEGG_ARACHIDONIC_ACID_METABOLISM	58	Arachidonic acid metabolism	6	7.69E-05	2.86E-03
KEGG_DILATED_CARDIOMYOPATHY	92	Dilated cardiomyopathy	7	1.41E-04	4.36E-03
KEGG_MATURITY_ONSET_DIABETES_OF_THE_YOUNG	25	Maturity onset diabetes of the young	4	2.28E-04	4.97E-03
KEGG_N_GLYCAN_BIOSYNTHESIS	46	N-Glycan biosynthesis	5	2.44E-04	4.97E-03
KEGG_GLYCOSPHINGOLIPID_BIOSYNTHESIS_LACTO_AND_NEO	26	Glycosphingolipid biosynthesis - lacto and neolacto se	4	2.67E-04	4.97E-03
KEGG_SELENOAMINO_ACID_METABOLISM	26	Selenoamino acid metabolism	4	2.67E-04	4.97E-03
KEGG_GLYCOSPHINGOLIPID_BIOSYNTHESIS_GLOBO_SERIES	14	Glycosphingolipid biosynthesis - globo series	3	5.99E-04	9.52E-03
KEGG_VIBRIO_CHOLERAE_INFECTION	56	Vibrio cholerae infection	5	6.14E-04	9.52E-03
KEGG_JAK_STAT_SIGNALING_PATHWAY	155	Jak-STAT signaling pathway	8	6.82E-04	9.75E-03
KEGG_COMPLEMENT_AND_COAGULATION_CASCADES	69	Complement and coagulation cascades	5	1.59E-03	1.99E-02
KEGG_MELANOGENESIS	102	Melanogenesis	6	1.63E-03	1.99E-02
KEGG_ALDOSTERONE_REGULATED_SODIUM_REABSORPTION	42	Aldosterone-regulated sodium reabsorption	4	1.71E-03	1.99E-02
KEGG_VASOPRESSIN_REGULATED_WATER_REABSORPTION	44	Vasopressin-regulated water reabsorption	4	2.04E-03	2.23E-02
KEGG_ARRHYTHMOGENIC_RIGHT_VENTRICULAR_CARDIOMYOF	76	Arrhythmogenic right ventricular cardiomyopathy (AR	5	2.43E-03	2.38E-02
KEGG_PATHWAYS_IN_CANCER	328	Pathways in cancer	11	2.60E-03	2.38E-02
KEGG_NITROGEN_METABOLISM	23	Nitrogen metabolism	3	2.69E-03	2.38E-02
KEGG_PROXIMAL_TUBULE_BICARBONATE_RECLAMATION	23	Proximal tubule bicarbonate reclamation	3	2.69E-03	2.38E-02
KEGG_HYPERTROPHIC_CARDIOMYOPATHY_HCM	85	Hypertrophic cardiomyopathy (HCM)	5	3.95E-03	3.34E-02
KEGG_ARGININE_AND_PROLINE_METABOLISM	54	Arginine and proline metabolism	4	4.32E-03	3.38E-02
KEGG_ERBB_SIGNALING_PATHWAY	87	ErbB signaling pathway	5	4.36E-03	3.38E-02
KEGG_HEMATOPOIETIC_CELL_LINEAGE	88	Hematopoietic cell lineage	5	4.58E-03	3.41E-02
KEGG_GAP_JUNCTION	90	Gap junction	5	5.04E-03	3.60E-02
KEGG_CALCIUM_SIGNALING_PATHWAY	178	Calcium signaling pathway	7	6.56E-03	4.52E-02
KEGG_ALANINE_ASPARTATE_AND_GLUTAMATE_METABOLISM	32	Alanine, aspartate and glutamate metabolism	3	6.93E-03	4.61E-02

48h GW3965 up-regulated					
Gene Set Name	# Genes	Description	# Genes in Overlap (k)	p-value	FDR q-value
KEGG_GLYCEROPHOSPHOLIPID_METABOLISM	77	Glycerophospholipid metabolism	19	2.01E-12	2.54E-10
KEGG_PPAR_SIGNALING_PATHWAY	69	PPAR signaling pathway	18	2.73E-12	2.54E-10
KEGG_LYSOSOME	121	Lysosome	21	1.82E-10	1.13E-08
KEGG_ARACHIDONIC_ACID_METABOLISM	58	Arachidonic acid metabolism	14	2.21E-09	1.03E-07
KEGG_STEROID_BIOSYNTHESIS	17	Steroid biosynthesis	8	1.68E-08	6.24E-07
KEGG_SULFUR_METABOLISM	13	Sulfur metabolism	7	4.15E-08	1.29E-06
KEGG_MAPK_SIGNALING_PATHWAY	267	MAPK signaling pathway	27	1.04E-07	2.77E-06
KEGG_GLYCEROLIPID_METABOLISM	49	Glycerolipid metabolism	11	2.59E-07	6.03E-06
KEGG_GLYCOLYSIS_GLUCONEOGENESIS	62	Glycolysis / Gluconeogenesis	12	4.19E-07	8.65E-06
KEGG_ETHER_LIPID_METABOLISM	33	Ether lipid metabolism	9	5.39E-07	1.00E-05
KEGG_PATHWAYS_IN_CANCER	328	Pathways in cancer	29	6.20E-07	1.05E-05
KEGG_BUTANOATE_METABOLISM	34	Butanoate metabolism	9	7.13E-07	1.11E-05
KEGG_PEROXISOME	78	Peroxisome	13	8.51E-07	1.22E-05
KEGG_FC_GAMMA_R_MEDIATED_PHAGOCYTOSIS	97	Fc gamma R-mediated phagocytosis	14	1.96E-06	2.61E-05
KEGG_VEGF_SIGNALING_PATHWAY	76	VEGF signaling pathway	12	4.02E-06	4.99E-05
KEGG_PENTOSE_PHOSPHATE_PATHWAY	27	Pentose phosphate pathway	7	1.46E-05	1.70E-04
KEGG_ENDOCYTOSIS	183	Endocytosis	18	1.94E-05	2.12E-04
KEGG_SPHINGOLIPID_METABOLISM	40	Sphingolipid metabolism	8	2.80E-05	2.89E-04
KEGG_FATTY_ACID_METABOLISM	42	Fatty acid metabolism	8	4.06E-05	3.98E-04
KEGG_GLYCOSAMINOGLYCAN_BIOSYNTHESIS_CHONDROITIN_S	22	Glycosaminoglycan biosynthesis - chondroitin sulfate	6	4.45E-05	4.14E-04
KEGG_BASAL_CELL_CARCINOMA	55	Basal cell carcinoma	9	4.80E-05	4.25E-04
KEGG_HEDGEHOG_SIGNALING_PATHWAY	56	Hedgehog signaling pathway	9	5.56E-05	4.30E-04
KEGG_ABC_TRANSPORTERS	44	ABC transporters	8	5.77E-05	4.30E-04
KEGG_AMINO_SUGAR_AND_NUCLEOTIDE_SUGAR_METABOLISM	44	Amino sugar and nucleotide sugar metabolism	8	5.77E-05	4.30E-04
KEGG_VALINE_LEUCINE_AND_ISOLEUCINE_DEGRADATION	44	Valine, leucine and isoleucine degradation	8	5.77E-05	4.30E-04
KEGG_GLYCOSPHINGOLIPID_BIOSYNTHESIS_GANGLIO_SERIES	15	Glycosphingolipid biosynthesis - ganglio series	5	6.81E-05	4.69E-04
KEGG_TERPENOID_BACKBONE_BIOSYNTHESIS	15	Terpenoid backbone biosynthesis	5	6.81E-05	4.69E-04
KEGG_FRUCTOSE_AND_MANNOSE_METABOLISM	34	Fructose and mannose metabolism	7	7.32E-05	4.87E-04
KEGG_N_GLYCAN_BIOSYNTHESIS	46	N-Glycan biosynthesis	8	8.04E-05	5.16E-04
KEGG_GALACTOSE_METABOLISM	26	Galactose metabolism	6	1.23E-04	7.65E-04
KEGG_CALCIUM_SIGNALING_PATHWAY	178	Calcium signaling pathway	16	1.60E-04	9.58E-04
KEGG_AXON_GUIDANCE	129	Axon guidance	13	2.10E-04	1.22E-03
KEGG_GNRH_SIGNALING_PATHWAY	101	GnRH signaling pathway	11	3.23E-04	1.72E-03
KEGG_METABOLISM_OF_XENOBIOTICS_BY_CYTOCHROME_P45	70	Metabolism of xenobiotics by cytochrome P450	9	3.23E-04	1.72E-03
KEGG_PANCREATIC_CANCER	70	Pancreatic cancer	9	3.23E-04	1.72E-03
KEGG_INSULIN_SIGNALING_PATHWAY	137	Insulin signaling pathway	13	3.79E-04	1.96E-03
KEGG_GLYCOSAMINOGLYCAN_DEGRADATION	21	Glycosaminoglycan degradation	5	3.95E-04	1.99E-03
KEGG_BIOSYNTHESIS_OF_UNSATURATED_FATTY_ACIDS	22	Biosynthesis of unsaturated fatty acids	5	4.98E-04	2.44E-03
KEGG_PHOSPHATIDYLINOSITOL_SIGNALING_SYSTEM	76	Phosphatidylinositol signaling system	9	5.99E-04	2.86E-03
KEGG_FC_EPSILON_RI_SIGNALING_PATHWAY	79	Fc epsilon RI signaling pathway	9	7.97E-04	3.70E-03
KEGG_MATURITY_ONSET_DIABETES_OF_THE_YOUNG	25	Maturity onset diabetes of the young	5	9.30E-04	4.22E-03
KEGG_WNT_SIGNALING_PATHWAY	151	Wnt signaling pathway	13	9.53E-04	4.22E-03
KEGG_GLYCOSPHINGOLIPID_BIOSYNTHESIS_LACTO_AND_NEO	26	Glycosphingolipid biosynthesis - lacto and neolacto se	5	1.12E-03	4.85E-03
KEGG_LEUKOCYTE_TRANSENDOTHELIAL_MIGRATION	118	Leukocyte transendothelial migration	11	1.20E-03	5.09E-03
KEGG_INOSITOL_PHOSPHATE_METABOLISM	54	Inositol phosphate metabolism	7	1.40E-03	5.61E-03
KEGG_NON_SMALL_CELL_LUNG_CANCER	54	Non-small cell lung cancer	7	1.40E-03	5.61E-03
KEGG_PYRUVATE_METABOLISM	40	Pyruvate metabolism	6	1.42E-03	5.61E-03
KEGG_CYTOKINE_CYTOKINE_RECEPTOR_INTERACTION	267	Cytokine-cytokine receptor interaction	18	2.00E-03	7.59E-03
KEGG_GAP_JUNCTION	90	Gap junction	9	2.02E-03	7.59E-03
KEGG_PHENYLALANINE_METABOLISM	18	Phenylalanine metabolism	4	2.04E-03	7.59E-03
KEGG_DILATED_CARDIOMYOPATHY	92	Dilated cardiomyopathy	9	2.35E-03	8.57E-03
KEGG_COLORECTAL_CANCER	62	Colorectal cancer	7	3.14E-03	1.12E-02
KEGG_BASE_EXCISION_REPAIR	35	Base excision repair	5	4.39E-03	1.51E-02
KEGG_PRION_DISEASES	35	Prion diseases	5	4.39E-03	1.51E-02
KEGG_ADIPOCYTOKINE_SIGNALING_PATHWAY	67	Adipocytokine signaling pathway	7	4.86E-03	1.64E-02
KEGG_TGF_BETA_SIGNALING_PATHWAY	86	TGF-beta signaling pathway	8	5.48E-03	1.76E-02
KEGG_ENDOMETRIAL_CANCER	52	Endometrial cancer	6	5.48E-03	1.76E-02
KEGG_TASTE_TRANSDUCTION	52	Taste transduction	6	5.48E-03	1.76E-02
KEGG_LONG_TERM_DEPRESSION	70	Long-term depression	7	6.18E-03	1.95E-02
KEGG_APOPTOSIS	88	Apoptosis	8	6.28E-03	1.95E-02
KEGG_ARGININE_AND_PROLINE_METABOLISM	54	Arginine and proline metabolism	6	6.60E-03	2.01E-02
KEGG_ANTIGEN_PROCESSING_AND_PRESENTATION	89	Antigen processing and presentation	8	6.72E-03	2.02E-02
KEGG_CHEMOKINE_SIGNALING_PATHWAY	190	Chemokine signaling pathway	13	7.00E-03	2.07E-02
KEGG_STEROID_HORMONE_BIOSYNTHESIS	55	Steroid hormone biosynthesis	6	7.22E-03	2.10E-02
KEGG_REGULATION_OF_ACTIN_CYTOSKELETON	216	Regulation of actin cytoskeleton	14	8.33E-03	2.38E-02
KEGG_B_CELL_RECEPTOR_SIGNALING_PATHWAY	75	B cell receptor signaling pathway	7	8.96E-03	2.52E-02
KEGG_CELL_ADHESION_MOLECULES_CAMS	134	Cell adhesion molecules (CAMs)	10	9.54E-03	2.59E-02
KEGG_BLADDER_CANCER	42	Bladder cancer	5	9.62E-03	2.59E-02
KEGG_TYROSINE_METABOLISM	42	Tyrosine metabolism	5	9.62E-03	2.59E-02
KEGG_VASCULAR_SMOOTH_MUSCLE_CONTRACTION	115	Vascular smooth muscle contraction	9	1.01E-02	2.69E-02
KEGG_GLYCOSAMINOGLYCAN_BIOSYNTHESIS_KERATAN_SULFA	15	Glycosaminoglycan biosynthesis - keratan sulfate	3	1.04E-02	2.73E-02
KEGG_FOCAL_ADHESION	201	Focal adhesion	13	1.09E-02	2.82E-02
KEGG_HISTIDINE_METABOLISM	29	Histidine metabolism	4	1.21E-02	3.01E-02
KEGG_LINOLEIC_ACID_METABOLISM	29	Linoleic acid metabolism	4	1.21E-02	3.01E-02
KEGG_NEUROACTIVE_LIGAND_RECEPTOR_INTERACTION	272	Neuroactive ligand-receptor interaction	16	1.21E-02	3.01E-02
KEGG_OTHER_GLYCAN_DEGRADATION	16	Other glycan degradation	3	1.25E-02	3.03E-02
KEGG_PRIMARY_BILE_ACID_BIOSYNTHESIS	16	Primary bile acid biosynthesis	3	1.25E-02	3.03E-02
KEGG_MELANOGENESIS	102	Melanogenesis	8	1.47E-02	3.50E-02
KEGG_TYPE_II_DIABETES_MELLITUS	47	Type II diabetes mellitus	5	1.53E-02	3.60E-02
KEGG_ECM_RECEPTOR_INTERACTION	84	ECM-receptor interaction	7	1.61E-02	3.70E-02
KEGG_SMALL_CELL_LUNG_CANCER	84	Small cell lung cancer	7	1.61E-02	3.70E-02
KEGG_HYPERTROPHIC_CARDIOMYOPATHY_HCM	85	Hypertrophic cardiomyopathy (HCM)	7	1.71E-02	3.87E-02
KEGG_PROPANOATE_METABOLISM	33	Propanoate metabolism	4	1.89E-02	4.23E-02
KEGG_GLUTATHIONE_METABOLISM	50	Glutathione metabolism	5	1.96E-02	4.33E-02
KEGG_T_CELL_RECEPTOR_SIGNALING_PATHWAY	108	T cell receptor signaling pathway	8	2.01E-02	4.38E-02
KEGG_ALPHA_LINOLENIC_ACID_METABOLISM	19	alpha-Linolenic acid metabolism	3	2.03E-02	4.38E-02
KEGG_COMPLEMENT_AND_COAGULATION_CASCADES	69	Complement and coagulation cascades	6	2.07E-02	4.38E-02
KEGG_P53_SIGNALING_PATHWAY	69	p53 signaling pathway	6	2.07E-02	4.38E-02
KEGG_PROSTATE_CANCER	89	Prostate cancer	7	2.15E-02	4.48E-02
KEGG_STARCH_AND_SUCROSE_METABOLISM	52	Starch and sucrose metabolism	5	2.28E-02	4.71E-02
KEGG_MELANOMA	71	Melanoma	6	2.35E-02	4.81E-02

48h GW3965 down-regulated					
Gene Set Name	# Genes	Description	# Genes in Overlap (k)	p-value	FDR q-value
KEGG_MAPK_SIGNALING_PATHWAY	267	MAPK signaling pathway	39	4.69E-17	8.73E-15
KEGG_PATHWAYS_IN_CANCER	328	Pathways in cancer	35	2.13E-11	1.98E-09
KEGG_P53_SIGNALING_PATHWAY	69	p53 signaling pathway	16	6.71E-11	4.16E-09
KEGG_BLADDER_CANCER	42	Bladder cancer	12	1.20E-09	5.56E-08
KEGG_CELL_CYCLE	128	Cell cycle	19	3.69E-09	1.37E-07
KEGG_TGF_BETA_SIGNALING_PATHWAY	86	TGF-beta signaling pathway	14	1.26E-07	3.91E-06
KEGG_AMINOACYL_TRNA_BIOSYNTHESIS	41	Aminoacyl-tRNA biosynthesis	9	1.68E-06	4.47E-05
KEGG_NEUROTROPHIN_SIGNALING_PATHWAY	126	Neurotrophin signaling pathway	14	1.35E-05	3.14E-04
KEGG_RENAL_CELL_CARCINOMA	70	Renal cell carcinoma	10	2.59E-05	4.93E-04
KEGG_CYTOKINE_CYTOKINE_RECEPTOR_INTERACTION	267	Cytokine-cytokine receptor interaction	21	2.65E-05	4.93E-04
KEGG_CHRONIC_MYELOID_LEUKEMIA	73	Chronic myeloid leukemia	10	3.76E-05	6.36E-04
KEGG_PROSTATE_CANCER	89	Prostate cancer	11	4.15E-05	6.43E-04
KEGG_NOD_LIKE_RECEPTOR_SIGNALING_PATHWAY	62	NOD-like receptor signaling pathway	9	5.74E-05	8.21E-04
KEGG_WNT_SIGNALING_PATHWAY	151	Wnt signaling pathway	14	1.01E-04	1.34E-03
KEGG_COMPLEMENT_AND_COAGULATION_CASCADES	69	Complement and coagulation cascades	9	1.35E-04	1.67E-03
KEGG_MELANOMA	71	Melanoma	9	1.68E-04	1.86E-03
KEGG_ERBB_SIGNALING_PATHWAY	87	ErbB signaling pathway	10	1.70E-04	1.86E-03
KEGG_COLORECTAL_CANCER	62	Colorectal cancer	8	3.40E-04	3.51E-03
KEGG_CIRCADIAN_RHYTHM_MAMMAL	13	Circadian rhythm - mammal	4	3.65E-04	3.57E-03
KEGG_REGULATION_OF_ACTIN_CYTOSKELETON	216	Regulation of actin cytoskeleton	16	4.52E-04	4.12E-03
KEGG_BASAL_TRANSCRIPTION_FACTORS	36	Basal transcription factors	6	4.65E-04	4.12E-03
KEGG_NATURAL_KILLER_CELL_MEDIATED_CYTOTOXICITY	137	Natural killer cell mediated cytotoxicity	12	5.20E-04	4.40E-03
KEGG_FOCAL_ADHESION	201	Focal adhesion	15	6.22E-04	4.96E-03
KEGG_EPITHELIAL_CELL_SIGNALING_IN_HELICOBACTER_PYLC	68	Epithelial cell signaling in Helicobacter pylori infection	8	6.40E-04	4.96E-03
KEGG_GLYCOSPHINGOLIPID_BIOSYNTHESIS_LACTO_AND_NEO	26	Glycosphingolipid biosynthesis - lacto and neolacto se	5	7.06E-04	5.25E-03
KEGG_PANCREATIC_CANCER	70	Pancreatic cancer	8	7.78E-04	5.57E-03
KEGG_HUNTINGTONS_DISEASE	185	Huntington's disease	14	8.12E-04	5.59E-03
KEGG_RIG_I_LIKE_RECEPTOR_SIGNALING_PATHWAY	71	RIG-I-like receptor signaling pathway	8	8.56E-04	5.68E-03
KEGG_AXON_GUIDANCE	129	Axon guidance	11	1.09E-03	7.01E-03
KEGG_ADHERENS_JUNCTION	75	Adherens junction	8	1.23E-03	7.62E-03
KEGG_LYSINE_DEGRADATION	44	Lysine degradation	6	1.39E-03	8.36E-03
KEGG_JAK_STAT_SIGNALING_PATHWAY	155	Jak-STAT signaling pathway	12	1.54E-03	8.92E-03
KEGG_ENDOCYTOSIS	183	Endocytosis	13	2.16E-03	1.22E-02
KEGG_TOLL_LIKE_RECEPTOR_SIGNALING_PATHWAY	102	Toll-like receptor signaling pathway	9	2.39E-03	1.31E-02
KEGG_SMALL_CELL_LUNG_CANCER	84	Small cell lung cancer	8	2.55E-03	1.35E-02
KEGG_SPLICEOSOME	128	Spliceosome	10	3.44E-03	1.73E-02
KEGG_T_CELL_RECEPTOR_SIGNALING_PATHWAY	108	T cell receptor signaling pathway	9	3.52E-03	1.73E-02
KEGG_LONG_TERM_POTENTIATION	70	Long-term potentiation	7	3.54E-03	1.73E-02
KEGG_SNARE_INTERACTIONS_IN_VESICULAR_TRANSPORT	38	SNARE interactions in vesicular transport	5	4.08E-03	1.93E-02
KEGG_LEISHMANIA_INFECTION	72	Leishmania infection	7	4.15E-03	1.93E-02
KEGG_B_CELL_RECEPTOR_SIGNALING_PATHWAY	75	B cell receptor signaling pathway	7	5.20E-03	2.36E-02
KEGG_RNA_DEGRADATION	59	RNA degradation	6	6.23E-03	2.76E-02
KEGG_ACUTE_MYELOID_LEUKEMIA	60	Acute myeloid leukemia	6	6.76E-03	2.92E-02
KEGG_THYROID_CANCER	29	Thyroid cancer	4	8.48E-03	3.58E-02
KEGG_GLIOMA	65	Glioma	6	9.92E-03	4.10E-02
KEGG_ALANINE_ASPARTATE_AND_GLUTAMATE_METABOLISM	32	Alanine, aspartate and glutamate metabolism	4	1.20E-02	4.86E-02

48h T0901317 up-regulated					
Gene Set Name	# Genes	Description	# Genes in Overlap (k)	p-value	FDR q-value
KEGG_METABOLISM_OF_XENOBIOTICS_BY_CYTOCHROME_P45	70	Metabolism of xenobiotics by cytochrome P450	18	2.52E-13	4.70E-11
KEGG_STEROID_BIOSYNTHESIS	17	Steroid biosynthesis	10	2.84E-12	2.64E-10
KEGG_PPAR_SIGNALING_PATHWAY	69	PPAR signaling pathway	16	2.96E-11	1.84E-09
KEGG_PORPHYRIN_AND_CHLOROPHYLL_METABOLISM	41	Porphyrin and chlorophyll metabolism	12	4.68E-10	2.18E-08
KEGG_DRUG_METABOLISM_CYTOCHROME_P450	72	Drug metabolism - cytochrome P450	15	6.18E-10	2.30E-08
KEGG_ARACHIDONIC_ACID_METABOLISM	58	Arachidonic acid metabolism	13	3.31E-09	1.03E-07
KEGG_GLYCOLYSIS_GLUCONEOGENESIS	62	Glycolysis / Gluconeogenesis	13	7.89E-09	1.93E-07
KEGG_ASCORBATE_AND_ALDARATE_METABOLISM	25	Ascorbate and aldarate metabolism	9	9.11E-09	1.93E-07
KEGG_STARCH_AND_SUCROSE_METABOLISM	52	Starch and sucrose metabolism	12	9.33E-09	1.93E-07
KEGG_BUTANOATE_METABOLISM	34	Butanoate metabolism	10	1.27E-08	2.36E-07
KEGG_STEROID_HORMONE_BIOSYNTHESIS	55	Steroid hormone biosynthesis	12	1.84E-08	3.12E-07
KEGG_PENTOSE_AND_GLUCURONATE_INTERCONVERSIONS	28	Pentose and glucuronate interconversions	9	2.87E-08	4.44E-07
KEGG_FATTY_ACID_METABOLISM	42	Fatty acid metabolism	10	1.17E-07	1.68E-06
KEGG_PEROXISOME	78	Peroxisome	13	1.41E-07	1.88E-06
KEGG_LYSOSOME	121	Lysosome	16	1.52E-07	1.88E-06
KEGG_RETINOL_METABOLISM	64	Retinol metabolism	11	9.35E-07	1.08E-05
KEGG_MAPK_SIGNALING_PATHWAY	267	MAPK signaling pathway	23	9.83E-07	1.08E-05
KEGG_TERPENOID_BACKBONE_BIOSYNTHESIS	15	Terpenoid backbone biosynthesis	6	1.44E-06	1.48E-05
KEGG_ABC_TRANSPORTERS	44	ABC transporters	9	2.01E-06	1.87E-05
KEGG_VALINE_LEUCINE_AND_ISOLEUCINE_DEGRADATION	44	Valine, leucine and isoleucine degradation	9	2.01E-06	1.87E-05
KEGG_DRUG_METABOLISM_OTHER_ENZYMES	51	Drug metabolism - other enzymes	9	7.29E-06	6.45E-05
KEGG_FC_GAMMA_R_MEDIATED_PHAGOCYTOSIS	97	Fc gamma R-mediated phagocytosis	12	1.07E-05	9.08E-05
KEGG_INSULIN_SIGNALING_PATHWAY	137	Insulin signaling pathway	14	1.90E-05	1.54E-04
KEGG_PATHWAYS_IN_CANCER	328	Pathways in cancer	23	2.91E-05	2.25E-04
KEGG_ENDOCYTOSIS	183	Endocytosis	16	3.51E-05	2.61E-04
KEGG_GLYCEROPHOSPHOLIPID_METABOLISM	77	Glycerophospholipid metabolism	10	3.77E-05	2.70E-04
KEGG_GLUTATHIONE_METABOLISM	50	Glutathione metabolism	8	4.92E-05	3.39E-04
KEGG_ADIPOCYTOKINE_SIGNALING_PATHWAY	67	Adipocytokine signaling pathway	9	7.00E-05	4.65E-04
KEGG_ARGININE_AND_PROLINE_METABOLISM	54	Arginine and proline metabolism	8	8.68E-05	5.57E-04
KEGG_TYROSINE_METABOLISM	42	Tyrosine metabolism	7	1.12E-04	6.92E-04
KEGG_ANTIGEN_PROCESSING_AND_PRESENTATION	89	Antigen processing and presentation	10	1.31E-04	7.87E-04
KEGG_ETHER_LIPID_METABOLISM	33	Ether lipid metabolism	6	2.11E-04	1.19E-03
KEGG_PROPANOATE_METABOLISM	33	Propanoate metabolism	6	2.11E-04	1.19E-03
KEGG_BASE_EXCISION_REPAIR	35	Base excision repair	6	2.95E-04	1.59E-03
KEGG_NITROGEN_METABOLISM	23	Nitrogen metabolism	5	3.00E-04	1.59E-03
KEGG_CALCIUM_SIGNALING_PATHWAY	178	Calcium signaling pathway	14	3.21E-04	1.66E-03
KEGG_GNRH_SIGNALING_PATHWAY	101	GnRH signaling pathway	10	3.72E-04	1.87E-03
KEGG_MELANOGENESIS	102	Melanogenesis	10	4.03E-04	1.97E-03
KEGG_GLYCOSPHINGOLIPID_BIOSYNTHESIS_GANGLIO_SERIES	15	Glycosphingolipid biosynthesis - ganglio series	4	5.40E-04	2.58E-03
KEGG_PYRUVATE_METABOLISM	40	Pyruvate metabolism	6	6.24E-04	2.90E-03
KEGG_PENTOSE_PHOSPHATE_PATHWAY	27	Pentose phosphate pathway	5	6.59E-04	2.99E-03
KEGG_VIBRIO_CHOLERAE_INFECTION	56	Vibrio cholerae infection	7	6.95E-04	3.02E-03
KEGG_AXON_GUIDANCE	129	Axon guidance	11	6.97E-04	3.02E-03
KEGG_WNT_SIGNALING_PATHWAY	151	Wnt signaling pathway	12	7.63E-04	3.23E-03
KEGG_DILATED_CARDIOMYOPATHY	92	Dilated cardiomyopathy	9	7.88E-04	3.26E-03
KEGG_ADHERENS_JUNCTION	75	Adherens junction	8	8.62E-04	3.48E-03
KEGG_PHOSPHATIDYLINOSITOL_SIGNALING_SYSTEM	76	Phosphatidylinositol signaling system	8	9.41E-04	3.65E-03
KEGG_VEGF_SIGNALING_PATHWAY	76	VEGF signaling pathway	8	9.41E-04	3.65E-03
KEGG_VASCULAR_SMOOTH_MUSCLE_CONTRACTION	115	Vascular smooth muscle contraction	10	1.03E-03	3.90E-03
KEGG_AMINO_SUGAR_AND_NUCLEOTIDE_SUGAR_METABOLISM	44	Amino sugar and nucleotide sugar metabolism	6	1.05E-03	3.90E-03
KEGG_PHENYLALANINE_METABOLISM	18	Phenylalanine metabolism	4	1.14E-03	4.15E-03
KEGG_GLYCEROLIPID_METABOLISM	49	Glycerolipid metabolism	6	1.86E-03	6.64E-03
KEGG_HYPERTROPHIC_CARDIOMYOPATHY_HCM	85	Hypertrophic cardiomyopathy (HCM)	8	1.95E-03	6.77E-03
KEGG_LIMONENE_AND_PINENE_DEGRADATION	10	Limonene and pinene degradation	3	1.97E-03	6.77E-03
KEGG_CYTOKINE_CYTOKINE_RECEPTOR_INTERACTION	267	Cytokine-cytokine receptor interaction	16	2.34E-03	7.92E-03
KEGG_BIOSYNTHESIS_OF_UNSATURATED_FATTY_ACIDS	22	Biosynthesis of unsaturated fatty acids	4	2.50E-03	8.29E-03
KEGG_PANCREATIC_CANCER	70	Pancreatic cancer	7	2.61E-03	8.50E-03
KEGG_NON_SMALL_CELL_LUNG_CANCER	54	Non-small cell lung cancer	6	3.06E-03	9.82E-03
KEGG_CELL_ADHESION_MOLECULES_CAMS	134	Cell adhesion molecules (CAMs)	10	3.23E-03	1.02E-02
KEGG_BASAL_CELL_CARCINOMA	55	Basal cell carcinoma	6	3.36E-03	1.04E-02
KEGG_SPHINGOLIPID_METABOLISM	40	Sphingolipid metabolism	5	4.04E-03	1.21E-02
KEGG_TRYPTOPHAN_METABOLISM	40	Tryptophan metabolism	5	4.04E-03	1.21E-02
KEGG_SULFUR_METABOLISM	13	Sulfur metabolism	3	4.41E-03	1.30E-02
KEGG_GLYCOSPHINGOLIPID_BIOSYNTHESIS_LACTO_AND_NEO	26	Glycosphingolipid biosynthesis - lacto and neolacto se	4	4.69E-03	1.36E-02
KEGG_PATHOGENIC_ESCHERICHIA_COLI_INFECTION	59	Pathogenic Escherichia coli infection	6	4.78E-03	1.37E-02
KEGG_FC_EPSILON_RI_SIGNALING_PATHWAY	79	Fc epsilon RI signaling pathway	7	5.13E-03	1.45E-02
KEGG_HISTIDINE_METABOLISM	29	Histidine metabolism	4	7.00E-03	1.94E-02
KEGG_GLIOMA	65	Glioma	6	7.66E-03	2.10E-02
KEGG_O_GLYCAN_BIOSYNTHESIS	30	O-Glycan biosynthesis	4	7.91E-03	2.13E-02
KEGG_TGF_BETA_SIGNALING_PATHWAY	86	TGF-beta signaling pathway	7	8.11E-03	2.13E-02
KEGG_OTHER_GLYCAN_DEGRADATION	16	Other glycan degradation	3	8.14E-03	2.13E-02
KEGG_GLYCINE_SERINE_AND_THREONINE_METABOLISM	31	Glycine, serine and threonine metabolism	4	8.89E-03	2.30E-02
KEGG_P53_SIGNALING_PATHWAY	69	p53 signaling pathway	6	1.02E-02	2.59E-02
KEGG_GAP_JUNCTION	90	Gap junction	7	1.03E-02	2.59E-02
KEGG_NATURAL_KILLER_CELL_MEDIATED_CYTOTOXICITY	137	Natural killer cell mediated cytotoxicity	9	1.13E-02	2.81E-02
KEGG_FRUCTOSE_AND_MANNOSE_METABOLISM	34	Fructose and mannose metabolism	4	1.23E-02	3.01E-02
KEGG_REGULATION_OF_ACTIN_CYTOSKELETON	216	Regulation of actin cytoskeleton	12	1.36E-02	3.29E-02
KEGG_INOSITOL_PHOSPHATE_METABOLISM	54	Inositol phosphate metabolism	5	1.43E-02	3.41E-02
KEGG_ALZHEIMERS_DISEASE	169	Alzheimer's disease	10	1.56E-02	3.66E-02
KEGG_ARRHYTHMOGENIC_RIGHT_VENTRICULAR_CARDIOMYOF	76	Arrhythmogenic right ventricular cardiomyopathy (AR	6	1.59E-02	3.69E-02
KEGG_HEDGEHOG_SIGNALING_PATHWAY	56	Hedgehog signaling pathway	5	1.66E-02	3.80E-02
KEGG_BETA_ALANINE_METABOLISM	22	beta-Alanine metabolism	3	1.99E-02	4.51E-02
KEGG_MISMATCH_REPAIR	23	Mismatch repair	3	2.24E-02	4.97E-02
KEGG_PROXIMAL_TUBULE_BICARBONATE_RECLAMATION	23	Proximal tubule bicarbonate reclamation	3	2.24E-02	4.97E-02

48h T0901317 down-regulated					
Gene Set Name	# Genes	Description	# Genes in Overlap (k)	p-value	FDR q-value
KEGG_MAPK_SIGNALING_PATHWAY	267	MAPK signaling pathway	36	1.77E-15	3.30E-13
KEGG_PATHWAYS_IN_CANCER	328	Pathways in cancer	37	2.08E-13	1.94E-11
KEGG_P53_SIGNALING_PATHWAY	69	p53 signaling pathway	14	3.32E-09	2.06E-07
KEGG_CIRCADIAN_RHYTHM_MAMMAL	13	Circadian rhythm - mammal	6	5.15E-07	2.40E-05
KEGG_AMINOACYL_TRNA_BIOSYNTHESIS	41	Aminoacyl-tRNA biosynthesis	9	1.07E-06	3.96E-05
KEGG_BLADDER_CANCER	42	Bladder cancer	9	1.32E-06	4.10E-05
KEGG_CELL_CYCLE	128	Cell cycle	15	1.76E-06	4.69E-05
KEGG_TGF_BETA_SIGNALING_PATHWAY	86	TGF-beta signaling pathway	12	3.01E-06	6.08E-05
KEGG_WNT_SIGNALING_PATHWAY	151	Wnt signaling pathway	16	3.09E-06	6.08E-05
KEGG_CYTOKINE_CYTOKINE_RECEPTOR_INTERACTION	267	Cytokine-cytokine receptor interaction	22	3.48E-06	6.08E-05
KEGG_CHRONIC_MYELOID_LEUKEMIA	73	Chronic myeloid leukemia	11	3.60E-06	6.08E-05
KEGG_ENDOCYTOSIS	183	Endocytosis	16	3.51E-05	5.35E-04
KEGG_NOD_LIKE_RECEPTOR_SIGNALING_PATHWAY	62	NOD-like receptor signaling pathway	9	3.74E-05	5.35E-04
KEGG_JAK_STAT_SIGNALING_PATHWAY	155	Jak-STAT signaling pathway	14	7.48E-05	9.77E-04
KEGG_EPITHELIAL_CELL_SIGNALING_IN_HELICOBACTER_PYLC	68	Epithelial cell signaling in Helicobacter pylori infection	9	7.88E-05	9.77E-04
KEGG_FOCAL_ADHESION	201	Focal adhesion	16	1.08E-04	1.18E-03
KEGG_ERBB_SIGNALING_PATHWAY	87	ErbB signaling pathway	10	1.08E-04	1.18E-03
KEGG_PROSTATE_CANCER	89	Prostate cancer	10	1.31E-04	1.35E-03
KEGG_NEUROTROPHIN_SIGNALING_PATHWAY	126	Neurotrophin signaling pathway	12	1.45E-04	1.42E-03
KEGG_ALANINE_ASPARTATE_AND_GLUTAMATE_METABOLISM	32	Alanine, aspartate and glutamate metabolism	6	1.76E-04	1.64E-03
KEGG_ACUTE_MYELOID_LEUKEMIA	60	Acute myeloid leukemia	8	1.86E-04	1.64E-03
KEGG_NOTCH_SIGNALING_PATHWAY	47	Notch signaling pathway	7	2.32E-04	1.96E-03
KEGG_SMALL_CELL_LUNG_CANCER	84	Small cell lung cancer	9	4.03E-04	3.26E-03
KEGG_RENAL_CELL_CARCINOMA	70	Renal cell carcinoma	8	5.42E-04	4.20E-03
KEGG_AXON_GUIDANCE	129	Axon guidance	11	6.97E-04	5.19E-03
KEGG_NATURAL_KILLER_CELL_MEDIATED_CYTOTOXICITY	137	Natural killer cell mediated cytotoxicity	11	1.15E-03	8.20E-03
KEGG_GLYCINE_SERINE_AND_THREONINE_METABOLISM	31	Glycine, serine and threonine metabolism	5	1.27E-03	8.53E-03
KEGG_COLORECTAL_CANCER	62	Colorectal cancer	7	1.28E-03	8.53E-03
KEGG_CHEMOKINE_SIGNALING_PATHWAY	190	Chemokine signaling pathway	13	1.86E-03	1.19E-02
KEGG_T_CELL_RECEPTOR_SIGNALING_PATHWAY	108	T cell receptor signaling pathway	9	2.44E-03	1.51E-02
KEGG_LEISHMANIA_INFECTION	72	Leishmania infection	7	3.06E-03	1.84E-02
KEGG_ADHERENS_JUNCTION	75	Adherens junction	7	3.85E-03	2.17E-02
KEGG_B_CELL_RECEPTOR_SIGNALING_PATHWAY	75	B cell receptor signaling pathway	7	3.85E-03	2.17E-02
KEGG_MATURITY_ONSET_DIABETES_OF_THE_YOUNG	25	Maturity onset diabetes of the young	4	4.05E-03	2.20E-02
KEGG_PHOSPHATIDYLINOSITOL_SIGNALING_SYSTEM	76	Phosphatidylinositol signaling system	7	4.14E-03	2.20E-02
KEGG_SPLICEOSOME	128	Spliceosome	9	7.44E-03	3.84E-02
KEGG_GLIOMA	65	Glioma	6	7.66E-03	3.85E-02
KEGG_APOPTOSIS	88	Apoptosis	7	9.16E-03	4.49E-02

48h Rosiglitazone up-regulated

Gene Set Name	# Genes	Description	# Genes in Overlap (k)	p-value	FDR q-value
KEGG_RETINOL_METABOLISM	64	Retinol metabolism	15	1.05E-13	1.95E-11
KEGG_PENTOSE_AND_GLUCURONATE_INTERCONVERSIONS	28	Pentose and glucuronate interconversions	11	3.44E-13	3.20E-11
KEGG_DRUG_METABOLISM_OTHER_ENZYMES	51	Drug metabolism - other enzymes	13	1.44E-12	7.92E-11
KEGG_PORPHYRIN_AND_CHLOROPHYLL_METABOLISM	41	Porphyrin and chlorophyll metabolism	12	1.70E-12	7.92E-11
KEGG_ASCORBATE_AND_ALDARATE_METABOLISM	25	Ascorbate and aldarate metabolism	10	3.35E-12	1.25E-10
KEGG_MAPK_SIGNALING_PATHWAY	267	MAPK signaling pathway	24	2.12E-11	6.58E-10
KEGG_STARCH_AND_SUCROSE_METABOLISM	52	Starch and sucrose metabolism	12	3.78E-11	1.00E-09
KEGG_DRUG_METABOLISM_CYTOCHROME_P450	72	Drug metabolism - cytochrome P450	13	1.57E-10	3.64E-09
KEGG_STEROID_HORMONE_BIOSYNTHESIS	55	Steroid hormone biosynthesis	11	1.29E-09	2.66E-08
KEGG_METABOLISM_OF_XENOBIOTICS_BY_CYTOCHROME_P45	70	Metabolism of xenobiotics by cytochrome P450	12	1.49E-09	2.77E-08
KEGG_PATHWAYS_IN_CANCER	328	Pathways in cancer	23	7.01E-09	1.19E-07
KEGG_PPAR_SIGNALING_PATHWAY	69	PPAR signaling pathway	11	1.59E-08	2.47E-07
KEGG_PHOSPHATIDYLINOSITOL_SIGNALING_SYSTEM	76	Phosphatidylinositol signaling system	10	4.63E-07	6.62E-06
KEGG_GLYCEROPHOSPHOLIPID_METABOLISM	77	Glycerophospholipid metabolism	10	5.24E-07	6.96E-06
KEGG_FOCAL_ADHESION	201	Focal adhesion	15	1.32E-06	1.64E-05
KEGG_GALACTOSE_METABOLISM	26	Galactose metabolism	6	3.25E-06	3.78E-05
KEGG_WNT_SIGNALING_PATHWAY	151	Wnt signaling pathway	12	7.88E-06	8.62E-05
KEGG_CALCIUM_SIGNALING_PATHWAY	178	Calcium signaling pathway	13	8.38E-06	8.66E-05
KEGG_AXON_GUIDANCE	129	Axon guidance	11	9.59E-06	9.39E-05
KEGG_FRUCTOSE_AND_MANNOSE_METABOLISM	34	Fructose and mannose metabolism	6	1.70E-05	1.58E-04
KEGG_GLYCOSPHINGOLIPID_BIOSYNTHESIS_LACTO_AND_NEO	26	Glycosphingolipid biosynthesis - lacto and neolacto se	5	5.69E-05	5.04E-04
KEGG_TGF_BETA_SIGNALING_PATHWAY	86	TGF-beta signaling pathway	8	8.47E-05	7.16E-04
KEGG_P53_SIGNALING_PATHWAY	69	p53 signaling pathway	7	1.36E-04	1.10E-03
KEGG_GLYCEROLIPID_METABOLISM	49	Glycerolipid metabolism	6	1.44E-04	1.11E-03
KEGG_PHENYLALANINE_METABOLISM	18	Phenylalanine metabolism	4	1.80E-04	1.34E-03
KEGG_BUTANOATE_METABOLISM	34	Butanoate metabolism	5	2.16E-04	1.55E-03
KEGG_INOSITOL_PHOSPHATE_METABOLISM	54	Inositol phosphate metabolism	6	2.47E-04	1.70E-03
KEGG_MELANOGENESIS	102	Melanogenesis	8	2.79E-04	1.85E-03
KEGG_BETA_ALANINE_METABOLISM	22	beta-Alanine metabolism	4	4.09E-04	2.63E-03
KEGG_ACUTE_MYELOID_LEUKEMIA	60	Acute myeloid leukemia	6	4.42E-04	2.74E-03
KEGG_COLORECTAL_CANCER	62	Colorectal cancer	6	5.28E-04	3.17E-03
KEGG_FATTY_ACID_METABOLISM	42	Fatty acid metabolism	5	5.93E-04	3.45E-03
KEGG_ADIPOCYTOKINE_SIGNALING_PATHWAY	67	Adipocytokine signaling pathway	6	8.00E-04	4.51E-03
KEGG_PENTOSE_PHOSPHATE_PATHWAY	27	Pentose phosphate pathway	4	9.21E-04	5.04E-03
KEGG_NOTCH_SIGNALING_PATHWAY	47	Notch signaling pathway	5	1.00E-03	5.17E-03
KEGG_TYPE_II_DIABETES_MELLITUS	47	Type II diabetes mellitus	5	1.00E-03	5.17E-03
KEGG_SULFUR_METABOLISM	13	Sulfur metabolism	3	1.10E-03	5.53E-03
KEGG_LEISHMANIA_INFECTION	72	Leishmania infection	6	1.17E-03	5.72E-03
KEGG_LINOLEIC_ACID_METABOLISM	29	Linoleic acid metabolism	4	1.21E-03	5.79E-03
KEGG_GLUTATHIONE_METABOLISM	50	Glutathione metabolism	5	1.33E-03	6.17E-03
KEGG_GNRH_SIGNALING_PATHWAY	101	GnRH signaling pathway	7	1.38E-03	6.27E-03
KEGG_TOLL_LIKE_RECEPTOR_SIGNALING_PATHWAY	102	Toll-like receptor signaling pathway	7	1.46E-03	6.48E-03
KEGG_PEROXISOME	78	Peroxisome	6	1.77E-03	7.66E-03
KEGG_BASAL_CELL_CARCINOMA	55	Basal cell carcinoma	5	2.04E-03	8.62E-03
KEGG_ARACHIDONIC_ACID_METABOLISM	58	Arachidonic acid metabolism	5	2.58E-03	1.02E-02
KEGG_ECM_RECEPTOR_INTERACTION	84	ECM-receptor interaction	6	2.58E-03	1.02E-02
KEGG_SMALL_CELL_LUNG_CANCER	84	Small cell lung cancer	6	2.58E-03	1.02E-02
KEGG_VASCULAR_SMOOTH_MUSCLE_CONTRACTION	115	Vascular smooth muscle contraction	7	2.90E-03	1.12E-02
KEGG_ERBB_SIGNALING_PATHWAY	87	ErbB signaling pathway	6	3.08E-03	1.17E-02
KEGG_GLYCOLYSIS_GLUCONEOGENESIS	62	Glycolysis / Gluconeogenesis	5	3.45E-03	1.26E-02
KEGG_ALPHA_LINOLENIC_ACID_METABOLISM	19	alpha-Linolenic acid metabolism	3	3.46E-03	1.26E-02
KEGG_NEUROTROPHIN_SIGNALING_PATHWAY	126	Neurotrophin signaling pathway	7	4.79E-03	1.64E-02
KEGG_CYTOKINE_CYTOKINE_RECEPTOR_INTERACTION	267	Cytokine-cytokine receptor interaction	11	4.82E-03	1.64E-02
KEGG_ALDOSTERONE_REGULATED_SODIUM_REABSORPTION	42	Aldosterone-regulated sodium reabsorption	4	4.84E-03	1.64E-02
KEGG_TYROSINE_METABOLISM	42	Tyrosine metabolism	4	4.84E-03	1.64E-02
KEGG_NEUROACTIVE_LIGAND_RECEPTOR_INTERACTION	272	Neuroactive ligand-receptor interaction	11	5.53E-03	1.84E-02
KEGG_AMINO_SUGAR_AND_NUCLEOTIDE_SUGAR_METABOLISM	44	Amino sugar and nucleotide sugar metabolism	4	5.72E-03	1.86E-02
KEGG_RENAL_CELL_CARCINOMA	70	Renal cell carcinoma	5	5.81E-03	1.86E-02
KEGG_CHRONIC_MYELOID_LEUKEMIA	73	Chronic myeloid leukemia	5	6.93E-03	2.18E-02
KEGG_INSULIN_SIGNALING_PATHWAY	137	Insulin signaling pathway	7	7.50E-03	2.32E-02
KEGG_FC_EPSILON_RI_SIGNALING_PATHWAY	79	Fc epsilon RI signaling pathway	5	9.60E-03	2.93E-02
KEGG_LIMONENE_AND_PINENE_DEGRADATION	10	Limonene and pinene degradation	2	1.10E-02	3.30E-02
KEGG_THYROID_CANCER	29	Thyroid cancer	3	1.16E-02	3.41E-02
KEGG_ARGININE_AND_PROLINE_METABOLISM	54	Arginine and proline metabolism	4	1.17E-02	3.41E-02
KEGG_APOPTOSIS	88	Apoptosis	5	1.48E-02	4.18E-02
KEGG_HEMATOPOIETIC_CELL_LINEAGE	88	Hematopoietic cell lineage	5	1.48E-02	4.18E-02
KEGG_PATHOGENIC_ESCHERICHIA_COLI_INFECTION	59	Pathogenic Escherichia coli infection	4	1.58E-02	4.40E-02
KEGG_GAP_JUNCTION	90	Gap junction	5	1.62E-02	4.43E-02
KEGG_ETHER_LIPID_METABOLISM	33	Ether lipid metabolism	3	1.65E-02	4.44E-02
KEGG_DILATED_CARDIOMYOPATHY	92	Dilated cardiomyopathy	5	1.77E-02	4.69E-02
KEGG_CIRCADIAN_RHYTHM_MAMMAL	13	Circadian rhythm - mammal	2	1.85E-02	4.84E-02
KEGG_CELL_CYCLE	128	Cell cycle	6	1.90E-02	4.90E-02

48h Rosiglitazone down-regulated					
Gene Set Name	# Genes	Description	# Genes in Overlap (k)	p-value	FDR q-value
KEGG_PATHWAYS_IN_CANCER	328	Pathways in cancer	29	1.74E-08	3.23E-06
KEGG_CYTOKINE_CYTOKINE_RECEPTOR_INTERACTION	267	Cytokine-cytokine receptor interaction	23	7.98E-07	4.94E-05
KEGG_MAPK_SIGNALING_PATHWAY	267	MAPK signaling pathway	23	7.98E-07	4.94E-05
KEGG_AXON_GUIDANCE	129	Axon guidance	15	1.68E-06	7.79E-05
KEGG_MATURITY_ONSET_DIABETES_OF_THE_YOUNG	25	Maturity onset diabetes of the young	7	2.72E-06	8.75E-05
KEGG_TAURINE_AND_HYPOTAURINE_METABOLISM	10	Taurine and hypotaurine metabolism	5	2.82E-06	8.75E-05
KEGG_JAK_STAT_SIGNALING_PATHWAY	155	Jak-STAT signaling pathway	16	3.72E-06	9.88E-05
KEGG_O_GLYCAN_BIOSYNTHESIS	30	O-Glycan biosynthesis	7	1.03E-05	2.39E-04
KEGG_WNT_SIGNALING_PATHWAY	151	Wnt signaling pathway	15	1.18E-05	2.39E-04
KEGG_SMALL_CELL_LUNG_CANCER	84	Small cell lung cancer	11	1.28E-05	2.39E-04
KEGG_LEUKOCYTE_TRANSENDOTHELIAL_MIGRATION	118	Leukocyte transendothelial migration	13	1.47E-05	2.49E-04
KEGG_ALANINE_ASPARTATE_AND_GLUTAMATE_METABOLISM	32	Alanine, aspartate and glutamate metabolism	7	1.62E-05	2.51E-04
KEGG_CALCIUM_SIGNALING_PATHWAY	178	Calcium signaling pathway	16	2.16E-05	3.09E-04
KEGG_NICOTINATE_AND_NICOTINAMIDE_METABOLISM	24	Nicotinate and nicotinamide metabolism	6	2.93E-05	3.90E-04
KEGG_SELENOAMINO_ACID_METABOLISM	26	Selenoamino acid metabolism	6	4.80E-05	5.95E-04
KEGG_MELANOGENESIS	102	Melanogenesis	11	8.01E-05	9.31E-04
KEGG_LYSOSOME	121	Lysosome	12	8.77E-05	9.59E-04
KEGG_PHOSPHATIDYLINOSITOL_SIGNALING_SYSTEM	76	Phosphatidylinositol signaling system	9	1.72E-04	1.78E-03
KEGG_COMPLEMENT_AND_COAGULATION_CASCADES	69	Complement and coagulation cascades	8	4.53E-04	4.21E-03
KEGG_P53_SIGNALING_PATHWAY	69	p53 signaling pathway	8	4.53E-04	4.21E-03
KEGG_INOSITOL_PHOSPHATE_METABOLISM	54	Inositol phosphate metabolism	7	5.16E-04	4.57E-03
KEGG_CHEMOKINE_SIGNALING_PATHWAY	190	Chemokine signaling pathway	14	5.47E-04	4.63E-03
KEGG_VIBRIO_CHOLERAE_INFECTION	56	Vibrio cholerae infection	7	6.46E-04	5.08E-03
KEGG_REGULATION_OF_ACTIN_CYTOSKELETON	216	Regulation of actin cytoskeleton	15	6.56E-04	5.08E-03
KEGG_ARACHIDONIC_ACID_METABOLISM	58	Arachidonic acid metabolism	7	8.01E-04	5.96E-03
KEGG_VEGF_SIGNALING_PATHWAY	76	VEGF signaling pathway	8	8.69E-04	6.04E-03
KEGG_HISTIDINE_METABOLISM	29	Histidine metabolism	5	8.77E-04	6.04E-03
KEGG_VASOPRESSIN_REGULATED_WATER_REABSORPTION	44	Vasopressin-regulated water reabsorption	6	9.84E-04	6.53E-03
KEGG_FC_GAMMA_R_MEDIATED_PHAGOCYTOSIS	97	Fc gamma R-mediated phagocytosis	9	1.06E-03	6.78E-03
KEGG_ETHER_LIPID_METABOLISM	33	Ether lipid metabolism	5	1.61E-03	9.97E-03
KEGG_NEUROTROPHIN_SIGNALING_PATHWAY	126	Neurotrophin signaling pathway	10	1.88E-03	1.13E-02
KEGG_ERBB_SIGNALING_PATHWAY	87	ErbB signaling pathway	8	2.09E-03	1.22E-02
KEGG_DILATED_CARDIOMYOPATHY	92	Dilated cardiomyopathy	8	2.97E-03	1.68E-02
KEGG_CHRONIC_MYELOID_LEUKEMIA	73	Chronic myeloid leukemia	7	3.09E-03	1.68E-02
KEGG_BASAL_CELL_CARCINOMA	55	Basal cell carcinoma	6	3.16E-03	1.68E-02
KEGG_PURINE_METABOLISM	159	Purine metabolism	11	3.38E-03	1.74E-02
KEGG_SULFUR_METABOLISM	13	Sulfur metabolism	3	4.26E-03	2.14E-02
KEGG_PYRIMIDINE_METABOLISM	98	Pyrimidine metabolism	8	4.38E-03	2.14E-02
KEGG_GLYCOSPHINGOLIPID_BIOSYNTHESIS_LACTO_AND_NEO	26	Glycosphingolipid biosynthesis - lacto and neolacto se	4	4.49E-03	2.14E-02
KEGG_ALDOSTERONE_REGULATED_SODIUM_REABSORPTION	42	Aldosterone-regulated sodium reabsorption	5	4.74E-03	2.21E-02
KEGG_COLORECTAL_CANCER	62	Colorectal cancer	6	5.75E-03	2.61E-02
KEGG_GLYCOSAMINOGLYCAN_BIOSYNTHESIS_KERATAN_SULFA	15	Glycosaminoglycan biosynthesis - keratan sulfate	3	6.52E-03	2.89E-02
KEGG_N_GLYCAN_BIOSYNTHESIS	46	N-Glycan biosynthesis	5	7.01E-03	2.99E-02
KEGG_HYPERTROPHIC_CARDIOMYOPATHY_HCM	85	Hypertrophic cardiomyopathy (HCM)	7	7.15E-03	2.99E-02
KEGG_GLIOMA	65	Glioma	6	7.23E-03	2.99E-02
KEGG_NOTCH_SIGNALING_PATHWAY	47	Notch signaling pathway	5	7.68E-03	3.11E-02
KEGG_APOPTOSIS	88	Apoptosis	7	8.60E-03	3.40E-02
KEGG_PROSTATE_CANCER	89	Prostate cancer	7	9.13E-03	3.49E-02
KEGG_CELL_ADHESION_MOLECULES_CAMS	134	Cell adhesion molecules (CAMs)	9	9.19E-03	3.49E-02
KEGG_RENIN_ANGIOTENSIN_SYSTEM	17	Renin-angiotensin system	3	9.37E-03	3.49E-02
KEGG_GLUTATHIONE_METABOLISM	50	Glutathione metabolism	5	9.95E-03	3.63E-02
KEGG_PANCREATIC_CANCER	70	Pancreatic cancer	6	1.03E-02	3.68E-02
KEGG_NATURAL_KILLER_CELL_MEDIATED_CYTOTOXICITY	137	Natural killer cell mediated cytotoxicity	9	1.05E-02	3.70E-02
KEGG_LEISHMANIA_INFECTION	72	Leishmania infection	6	1.17E-02	3.99E-02
KEGG_BUTANOATE_METABOLISM	34	Butanoate metabolism	4	1.18E-02	3.99E-02
KEGG_ARGININE_AND_PROLINE_METABOLISM	54	Arginine and proline metabolism	5	1.36E-02	4.45E-02
KEGG_NON_SMALL_CELL_LUNG_CANCER	54	Non-small cell lung cancer	5	1.36E-02	4.45E-02
KEGG_B_CELL_RECEPTOR_SIGNALING_PATHWAY	75	B cell receptor signaling pathway	6	1.42E-02	4.54E-02
