# Supplementary material for: Distinct gene regulatory programs define the inhibitory effects of liver X receptors and PPARG on cancer cell proliferation
Source: Genome Med. 2016 Jul 11;8:74. doi: 10.1186/s13073-016-0328-6 (PMC4940857; doi:10.1186/s13073-016-0328-6)
Supplement: Additional file 3: Table S2. — (PDF 71 kb) [file 13073_2016_328_MOESM3_ESM.pdf]

TABLE S2

Rosiglitazone-GW3965 48 hr. up-regulated Pathways

Category	Term	Count	%	PValue	Genes	List Total	Pop Hits	Pop Total	Fold Enrichment	Bonferroni	Benjamini	FDR
INTERPRO	IPR000892:Ribosomal protein S26e	2	2.0408	0.0083	RPS26P35, RPS26P11	70	2	16659	237.9857143	0.786482171	0.786482171	9.770194776
PIR_SUPERFAMILY	PIRSF002153:ribosomal protein S26	2	2.0408	0.0094	RPS26P35, RPS26P11	36	2	7396	205.4444444	0.315801607	0.315801607	8.314242368
GOTERM_MF_FAT	GO:0030246~carbohydrate binding	6	6.1224	0.0144	CLEC1A, KLRK1, FSTL1, PLA2R1, SOD3, HMMR	54	354	12983	4.075015694	0.938403675	0.938403675	16.53406313
GOTERM_CC_FAT	GO:0043232~intracellular non-membrane-bounded organelle	21	21.429	0.0181	HAUS4, DLGAP1, MRPS23, NUP160, RPS26P11, FERMT3, FMR1, MYH7, RAD9A, DPYSL2, PTPN20B, NCAPD3, DCAF12, RPS26P35, HJURP, KRTAP4-7, PQBP1, PIWIL4, LMOD2, NFIB, MRPL44	63	2596	12782	1.641242938	0.933962926	0.933962926	19.55094888
GOTERM_CC_FAT	GO:0043228~non-membrane-bounded organelle	21	21.429	0.0181	HAUS4, DLGAP1, MRPS23, NUP160, RPS26P11, FERMT3, FMR1, MYH7, RAD9A, DPYSL2, PTPN20B, NCAPD3, DCAF12, RPS26P35, HJURP, KRTAP4-7, PQBP1, PIWIL4, LMOD2, NFIB, MRPL44	63	2596	12782	1.641242938	0.933962926	0.933962926	19.55094888
GOTERM_CC_FAT	GO:0005840~ribosome	5	5.102	0.0203	RPS26P35, MRPS23, RPS26P11, FMR1, MRPL44	63	215	12782	4.718346253	0.953078244	0.783385697	21.72194393
GOTERM_BP_FAT	GO:0008210~estrogen metabolic process	2	2.0408	0.0406	UGT1A7, HSD17B8	57	10	13528	47.46666667	0.999999997	0.999999997	44.7072844
SP_PIR_KEYWORDS	ribosomal protein	4	4.0816	0.041	RPS26P35, MRPS23, RPS26P11, MRPL44	79	188	19235	5.180447078	0.999584721	0.999584721	40.45704136
BIOCARTA	beta-arrestins in GPCR Desensitization	2	2.0408	0.0411	ARRB1, C22ORF40	6	12	1437	39.91666667	0.510215979	0.510215979	26.21483369
GOTERM_BP_FAT	GO:0006260~DNA replication	4	4.0816	0.0439	CCDC111, RAD9A, TK2, NFIB	57	190	13528	4.996491228	0.999999999	0.999974619	47.3551741
INTERPRO	IPR018378:C-type lectin, conserved site	3	3.0612	0.0454	CLEC1A, KLRK1, PLA2R1	70	82	16659	8.706794425	0.999823861	0.986728261	43.76219994
INTERPRO	IPR001304:C-type lectin	3	3.0612	0.0454	CLEC1A, KLRK1, PLA2R1	70	82	16659	8.706794425	0.999823861	0.986728261	43.76219994
SMART	SM00034:CLECT	3	3.0612	0.0481	CLEC1A, KLRK1, PLA2R1	40	82	9079	8.303963415	0.939942364	0.939942364	38.68099943
INTERPRO	IPR016186:C-type lectin-like	3	3.0612	0.059	CLEC1A, KLRK1, PLA2R1	70	95	16659	7.515338346	0.999987794	0.976975717	52.92005966
INTERPRO	IPR018029:C2 membrane targeting protein	3	3.0612	0.0715	WWP1, PIK3C2B, SYT9	70	106	16659	6.735444744	0.999998977	0.96819934	60.0850407
SP_PIR_KEYWORDS	cytoplasmic vesicle	4	4.0816	0.0725	ARRB1, ESD, SYT9, RAB13	79	238	19235	4.09211786	0.999999163	0.999085219	60.61458396
SP_PIR_KEYWORDS	methylation	4	4.0816	0.0754	FMR1, MYH7, PIWIL4, RAB13	79	242	19235	4.024479548	0.999999531	0.992229346	62.10298317
GOTERM_BP_FAT	GO:0005976~polysaccharide metabolic process	3	3.0612	0.0772	GLT25D2, ITIH4, DSE	57	111	13528	6.414414414	1	0.999996673	68.2476095
SP_PIR_KEYWORDS	polymorphism	54	55.102	0.0936	ESD, SYT9, C2ORF70, ZNF347, DSE, DUPD1, DCAF12, TSKS, GLT25D2, WWP1, PQBP1, ITIH4, PIWIL4, MAPKBP1, ZCCHC8, SQRDL, LYRM2, ZCCHC11, FMR1, RAD9A, MYH7, SEL1L3, NCAPD3, CLEC1A, ZDHHC14, SERPINB7, MRPL44, SLC7A6OS, NUP160, KLRK1, MFSD1, OR10G2, HMMR, UGT1A7, SSX2B, HJURP, DYRK3, PLA2R1, HSD17B8, DLGAP1, OR8G5, C17ORF80, SLC6A16, DPYSL2, COL16A1, TSGA10IP, SOD3, CAPN1, CCDC111, NRAP, PDE7A, TREML2, C22ORF40, MUC16	79	11550	19235	1.138352786	0.999999989	0.989652852	70.40194614
GOTERM_MF_FAT	GO:0004857~enzyme inhibitor activity	4	4.0816	0.0977	ABCE1, ARRB1, SERPINB7, ITIH4	54	270	12983	3.561865569	0.999999997	0.999948439	72.20704523

Rosiglitazone-GW3965 48 hr. down-regulated Pathways

Category	Term	Count	%	PValue	Genes	List Total	Pop Hits	Pop Total	Fold Enrichment	Bonferroni	Benjamini	FDR
GOTERM_CC_FAT	GO:0005886~plasma membrane	16	21.622	0.0146	JUB, OR51D1, FLOT1, OR52E2, NKAIN1, PCDHB13, HCFC2, OR2V1, SHANK3, SCFD1, KCNJ8, RRAS2, GLP2R, CD6, PHLDA3, SEMA4A	31	3777	12782	1.746667008	0.773332387	0.773332387	15.07645371
UP_SEQ_FEATURE	compositionally biased region:Poly-Arg	4	5.4054	0.0184	ASCL2, NBPF11, SYNPO2, EPS8L1	62	175	19113	7.046267281	0.988469608	0.988469608	21.33825438
GOTERM_BP_FAT	GO:0001701~in utero embryonic development	4	5.4054	0.0194	ASCL2, XRCC2, KLF1, FOXD3	45	176	13528	6.832323232	0.999663198	0.999663198	23.9776484
UP_SEQ_FEATURE	domain:PDZ	3	4.0541	0.0482	PDZD8, SYNPO2, SHANK3	62	110	19113	8.407478006	0.999992927	0.997340491	47.15483211
SP_PIR_KEYWORDS	nucleus	20	27.027	0.0686	GPS1, CPSF3L, JUB, TBL3, TSPY1, XRCC2, YY2, GINS4, GTF2IRD2, ZNF648, SYNPO2, HCFC2, PADI4, ASCL2, GABPB1, POLDIP3, BRDT, ZNF587, KLF1, FOXD3	62	4283	19235	1.448713217	0.999895956	0.999895956	56.25299119
UP_SEQ_FEATURE	sequence variant	45	60.811	0.0809	PRR18, C1ORF174, TSPY1, XRCC2, OR51D1, FAM3D, ADPGK, PCDHB13, HCFC2, LY9, MUDENG, GABPB1, C6ORF97, NKPD1, LRRC56, ELOVL4, NDUFS8, LONRF3, GLP2R, CD6, PHLDA3, UNC45B, CDK13, TBL3, YY2, FLOT1, NBPF11, GTF2IRD2, OR52E2, ZNF648, SYNPO2, PADI4, PIH1D2, SHANK3, C5ORF32, SCFD1, PDZD8, DUSP1, KCNJ8, RRAS2, BRDT, CPD, KLF1, SEMA4A, EPS8L1	62	11992	19113	1.156799425	0.999999998	0.998823324	66.32024654
INTERPRO	IPR001478:PDZ/DHR/GLGF	3	4.0541	0.0841	PDZD8, SYNPO2, SHANK3	55	149	16659	6.09847468	0.999986987	0.999986987	63.96446271
UP_SEQ_FEATURE	compositionally biased region:Pro-rich	7	9.4595	0.0849	C5ORF32, PRR18, PDZD8, SYNPO2, KLF1, EPS8L1, SHANK3	62	960	19113	2.247832661	0.999999999	0.995120814	68.17958423
SMART	SM00228:PDZ	3	4.0541	0.0913	PDZD8, SYNPO2, SHANK3	32	149	9079	5.712458054	0.957618409	0.957618409	56.6601131
GOTERM_BP_FAT	GO:0043009~chordate embryonic development	4	5.4054	0.0923	ASCL2, XRCC2, KLF1, FOXD3	45	331	13528	3.632896945	1	0.999999997	74.20644573
GOTERM_BP_FAT	GO:0009792~embryonic development ending in birth or egg ha	4	5.4054	0.0942	ASCL2, XRCC2, KLF1, FOXD3	45	334	13528	3.600266134	1	0.999998576	74.95895146
SP_PIR_KEYWORDS	polymorphism	43	58.108	0.0999	PRR18, C1ORF174, XRCC2, OR51D1, TSPY1, FAM3D, ADPGK, PCDHB13, HCFC2, LY9, MUDENG, GABPB1, C6ORF97, NKPD1, LRRC56, ELOVL4, LONRF3, GLP2R, CD6, PHLDA3, UNC45B, CDK13, TBL3, YY2, FLOT1, NBPF11, GTF2IRD2, OR52E2, ZNF648, SYNPO2, PADI4, PIH1D2, SHANK3, C5ORF32, SCFD1, PDZD8, DUSP1, KCNJ8, BRDT, CPD, KLF1, SEMA4A, EPS8L1	62	11550	19235	1.155013266	0.999998724	0.998870294	70.57991039

Rosiglitazone-TO910317 48 hr. up-regulated Pathways

Category	Term	Count	%	PValue	Genes	List Total	Pop Hits	Pop Total	Fold Enrichment	Bonferroni	Benjamini	FDR
UP_SEQ_FEATURE	region of interest:Mediates transcriptional activation	2	0.5305	0.0063	YY2, NPAT	41	3	19113	310.7804878	0.579949075	0.579949075	7.130395066
GOTERM_BP_FAT	GO:0046676~negative regulation of insulin secretion	2	0.5305	0.0272	FAM3D, KCNQ1	32	12	13528	70.45833333	0.999966382	0.999966382	31.64044072
GOTERM_BP_FAT	GO:0002792~negative regulation of peptide secretion	2	0.5305	0.0272	FAM3D, KCNQ1	32	12	13528	70.45833333	0.999966382	0.999966382	31.64044072
GOTERM_BP_FAT	GO:0051051~negative regulation of transport	3	0.7958	0.0381	PRKCA, FAM3D, KCNQ1	32	135	13528	9.394444444	0.999999503	0.999294981	41.49242395
BIOCARTA	h_chemicalPathway:Apoptotic Signaling in Response to DNA Da	2	0.5305	0.0453	PRKCA, NPAT	4	22	1437	32.65909091	0.918005502	0.918005502	36.47547485
GOTERM_BP_FAT	GO:0046888~negative regulation of hormone secretion	2	0.5305	0.058	FAM3D, KCNQ1	32	26	13528	32.51923077	1	0.999414513	56.15910946
GOTERM_CC_FAT	GO:0015030~Cajal body	2	0.5305	0.0584	OIP5, NPAT	25	32	12782	31.955	0.99140791	0.99140791	47.19424142
GOTERM_BP_FAT	GO:0017148~negative regulation of translation	2	0.5305	0.0751	PRKCA, EIF2B3	32	34	13528	24.86764706	1	0.999325876	65.99447373
GOTERM_BP_FAT	GO:0051046~regulation of secretion	3	0.7958	0.0778	PRKCA, FAM3D, KCNQ1	32	202	13528	6.278465347	1	0.997659854	67.32323282
GOTERM_BP_FAT	GO:0050796~regulation of insulin secretion	2	0.5305	0.0878	FAM3D, KCNQ1	32	40	13528	21.1375	1	0.996747753	71.89658358
SP_PIR_KEYWORDS	activator	4	1.061	0.0931	YY2, NPAT, KLF1, FOXD3	41	520	19235	3.608818011	0.999988121	0.999988121	67.22107839
KEGG_PATHWAY	hsa05110:Vibrio cholerae infection	2	0.5305	0.0949	PRKCA, KCNQ1	10	56	5085	18.16071429	0.977410349	0.977410349	59.40069086

Rosiglitazone-TO910317 48 hr. down-regulated Pathways

Category	Term	Count	%	PValue	Genes	List Total	Pop Hits	Pop Total	Fold Enrichment	Bonferroni	Benjamini	FDR
SP_PIR_KEYWORDS	Lectin	4	0.7207	0.0054	CLEC1A, GALNT6, CLEC2A, PLA2R1	44	159	19235	10.99771298	0.52680824	0.52680824	6.14655106
SP_PIR_KEYWORDS	polymorphism	35	6.3063	0.0067	OSTF1, CLCN2, TSPY1, RAP1GAP, GALNT6, CYC1, UVRAG, C2ORF70, OR10G2, MUDENG, TSKS, ITGAX, HJURP, SLC2A2, WWP1, CLEC2A, ERAP1, QKI, GLP2R, PLA2R1, CD6, MAPKBP1, SQRDL, OR52E2, C17ORF65, SEL1L3, COL16A1, SHANK3, CLEC1A, JOSD1, DIO2, BNIP2, MYH14, PRHOXNB, CRYZL1	44	11550	19235	1.324724518	0.605926735	0.372247449	7.591112033
GOTERM_MF_FAT	GO:0005529~sugar binding	4	0.7207	0.012	CLEC1A, GALNT6, CLEC2A, PLA2R1	33	195	12983	8.07024087	0.765567865	0.765567865	12.95673021
SMART	SM00034:CLECT	3	0.5405	0.0166	CLEC1A, CLEC2A, PLA2R1	23	82	9079	14.4416755	0.373444999	0.373444999	13.03651265
UP_SEQ_FEATURE	sequence variant	35	6.3063	0.0169	OSTF1, CLCN2, TSPY1, RAP1GAP, GALNT6, CYC1, UVRAG, C2ORF70, OR10G2, MUDENG, TSKS, ITGAX, HJURP, SLC2A2, WWP1, CLEC2A, ERAP1, QKI, GLP2R, PLA2R1, CD6, MAPKBP1, SQRDL, OR52E2, C17ORF65, SEL1L3, COL16A1, SHANK3, CLEC1A, JOSD1, DIO2, BNIP2, MYH14, PRHOXNB, CRYZL1	44	11992	19113	1.267805431	0.960792535	0.960792535	19.09286645
INTERPRO	IPR018378:C-type lectin, conserved site	3	0.5405	0.0173	CLEC1A, CLEC2A, PLA2R1	42	82	16659	14.51132404	0.843312301	0.843312301	17.81596468
INTERPRO	IPR001304:C-type lectin	3	0.5405	0.0173	CLEC1A, CLEC2A, PLA2R1	42	82	16659	14.51132404	0.843312301	0.843312301	17.81596468
INTERPRO	IPR016186:C-type lectin-like	3	0.5405	0.0228	CLEC1A, CLEC2A, PLA2R1	42	95	16659	12.52556391	0.91365467	0.70615424	22.84010018
GOTERM_MF_FAT	GO:0017124~SH3 domain binding	3	0.5405	0.0232	UVRAG, QKI, SHANK3	33	96	12983	12.29450758	0.940489663	0.756052593	23.65576292
SP_PIR_KEYWORDS	oxidoreductase	5	0.9009	0.0363	SQRDL, DHFR, DIO2, CYC1, CRYZL1	44	562	19235	3.889315755	0.994101349	0.819316881	35.284482
GOTERM_BP_FAT	GO:0006417~regulation of translation	3	0.5405	0.0463	EIF4B, DIO2, QKI	35	137	13528	8.463816475	0.99999975	0.99999975	47.23793628
GOTERM_MF_FAT	GO:0019904~protein domain specific binding	4	0.7207	0.0472	UVRAG, QKI, LPAR2, SHANK3	33	331	12983	4.75437151	0.996996101	0.855712589	42.62630723
SP_PIR_KEYWORDS	nadp	3	0.5405	0.0475	SQRDL, DHFR, CRYZL1	44	156	19235	8.406905594	0.998849838	0.815842363	43.66005277
GOTERM_MF_FAT	GO:0030246~carbohydrate binding	4	0.7207	0.0557	CLEC1A, GALNT6, CLEC2A, PLA2R1	33	354	12983	4.445471666	0.99896741	0.820740576	48.19766884
UP_SEQ_FEATURE	domain:Ricin B-type lectin	2	0.3604	0.0569	GALNT6, PLA2R1	44	26	19113	33.41433566	0.999985389	0.996177524	51.72706998
SMART	SM00458:RICIN	2	0.3604	0.0635	GALNT6, PLA2R1	23	27	9079	29.23993559	0.840669963	0.600838332	42.23453187
UP_SEQ_FEATURE	glycosylation site:N-linked (GlcNAc...)	15	2.7027	0.0639	GALNT6, OR52E2, SEL1L3, LPAR2, OR10G2, COL16A1, CLEC1A, ITGAX, SLC2A2, CLEC2A, ERAP1, TCTN1, GLP2R, CD6, PLA2R1	44	4129	19113	1.57805654	0.999996413	0.984692477	55.9645087
INTERPRO	IPR000772:Ricin B lectin	2	0.3604	0.0644	GALNT6, PLA2R1	42	27	16659	29.38095238	0.999139467	0.904883494	52.62834388
UP_SEQ_FEATURE	topological domain:Cytoplasmic	13	2.3423	0.0645	CLEC1A, CLCN2, ITGAX, GALNT6, SLC2A2, CLEC2A, OR52E2, ERAP1, LPAR2, GLP2R, PLA2R1, CD6, OR10G2	44	3374	19113	1.673687827	0.999996836	0.957825949	56.32472963
SP_PIR_KEYWORDS	Signal-anchor	4	0.7207	0.0676	CLEC1A, GALNT6, CLEC2A, ERAP1	44	421	19235	4.153530555	0.999940059	0.856931049	56.14255812
GOTERM_BP_FAT	GO:0055114~oxidation reduction	5	0.9009	0.0747	SQRDL, DHFR, DIO2, CYC1, CRYZL1	35	639	13528	3.024368433	1	0.999996106	64.92783784
SP_PIR_KEYWORDS	glycoprotein	15	2.7027	0.0834	GALNT6, OR52E2, SEL1L3, LPAR2, OR10G2, COL16A1, CLEC1A, ITGAX, SLC2A2, CLEC2A, ERAP1, TCTN1, GLP2R, CD6, PLA2R1	44	4318	19235	1.518616573	0.999994506	0.867163633	64.18554397
GOTERM_MF_FAT	GO:0050661~NADP or NADPH binding	2	0.3604	0.0851	DHFR, CRYZL1	33	36	12983	21.85690236	0.999976892	0.881763165	63.98421122
GOTERM_BP_FAT	GO:0010608~posttranscriptional regulation of gene expression	3	0.5405	0.0982	EIF4B, DIO2, QKI	35	211	13528	5.495463778	1	0.999984215	75.21807697
